# Supplementary material for: Integrin β4 promotes DNA damage-related drug resistance in triple-negative breast cancer via TNFAIP2/IQGAP1/RAC1
Source: eLife. 2023 Oct 3;12:RP88483. doi: 10.7554/eLife.88483 (PMC10547475; doi:10.7554/eLife.88483)
Supplement: Figure 1—source data 1. [file elife-88483-fig1-data1.pptx]

## Slide 1
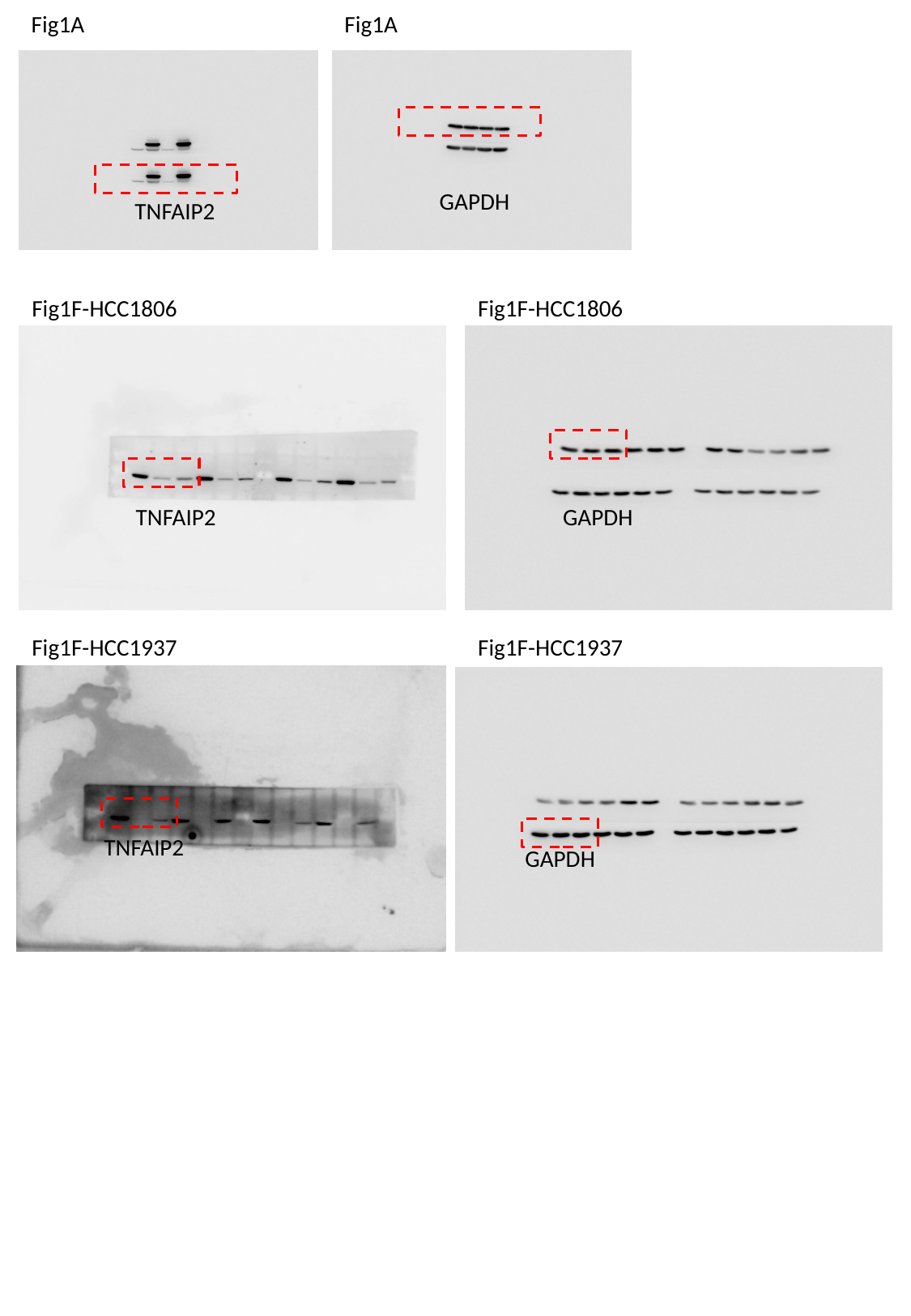

Fig1A
Fig1A
GAPDH
TNFAIP2
Fig1F-HCC1806
Fig1F-HCC1806
TNFAIP2
GAPDH
Fig1F-HCC1937
Fig1F-HCC1937
TNFAIP2
GAPDH

## Slide 2
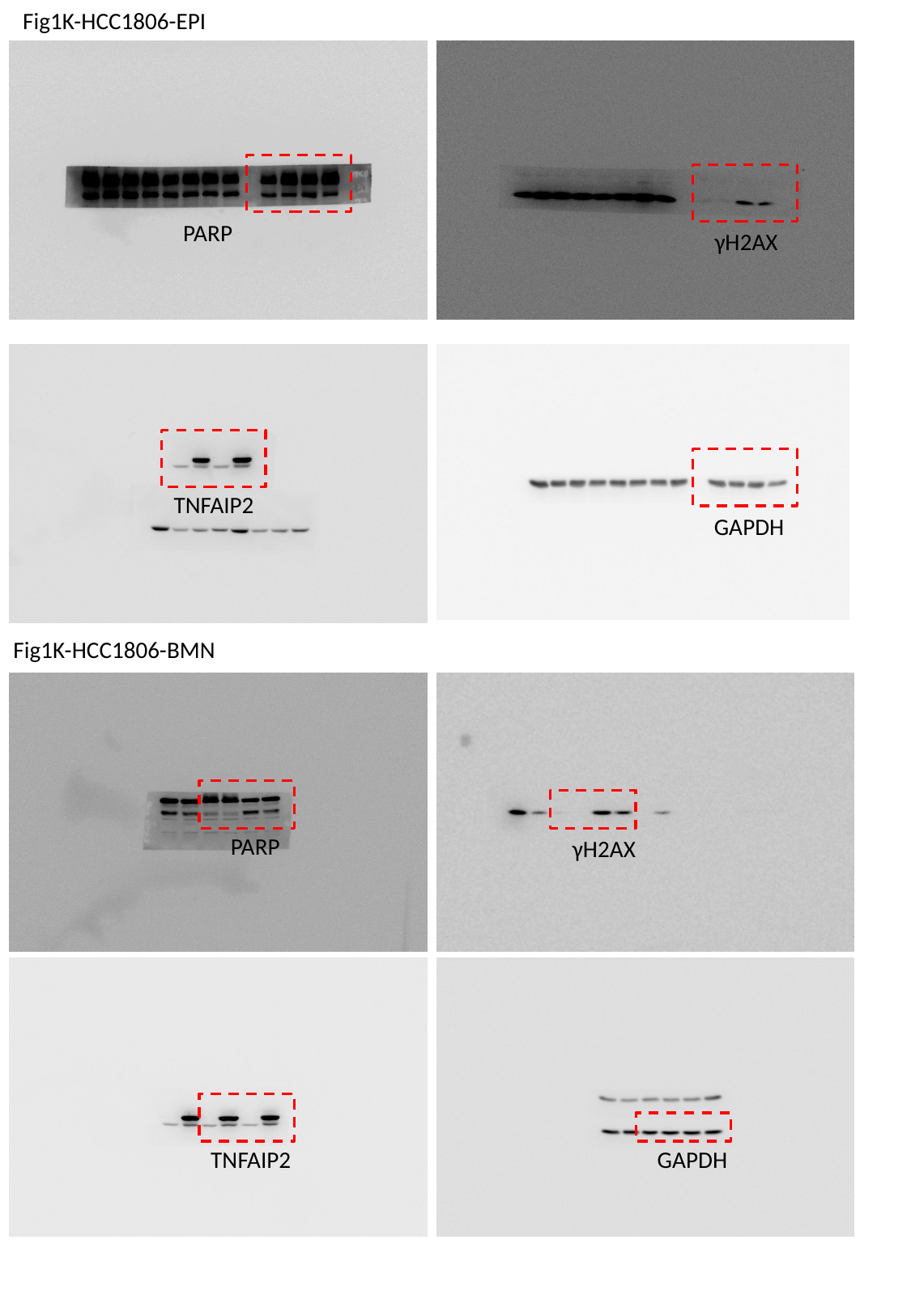

Fig1K-HCC1806-EPI
PARP
γH2AX
TNFAIP2
GAPDH
Fig1K-HCC1806-BMN
PARP
γH2AX
TNFAIP2
GAPDH

## Slide 3
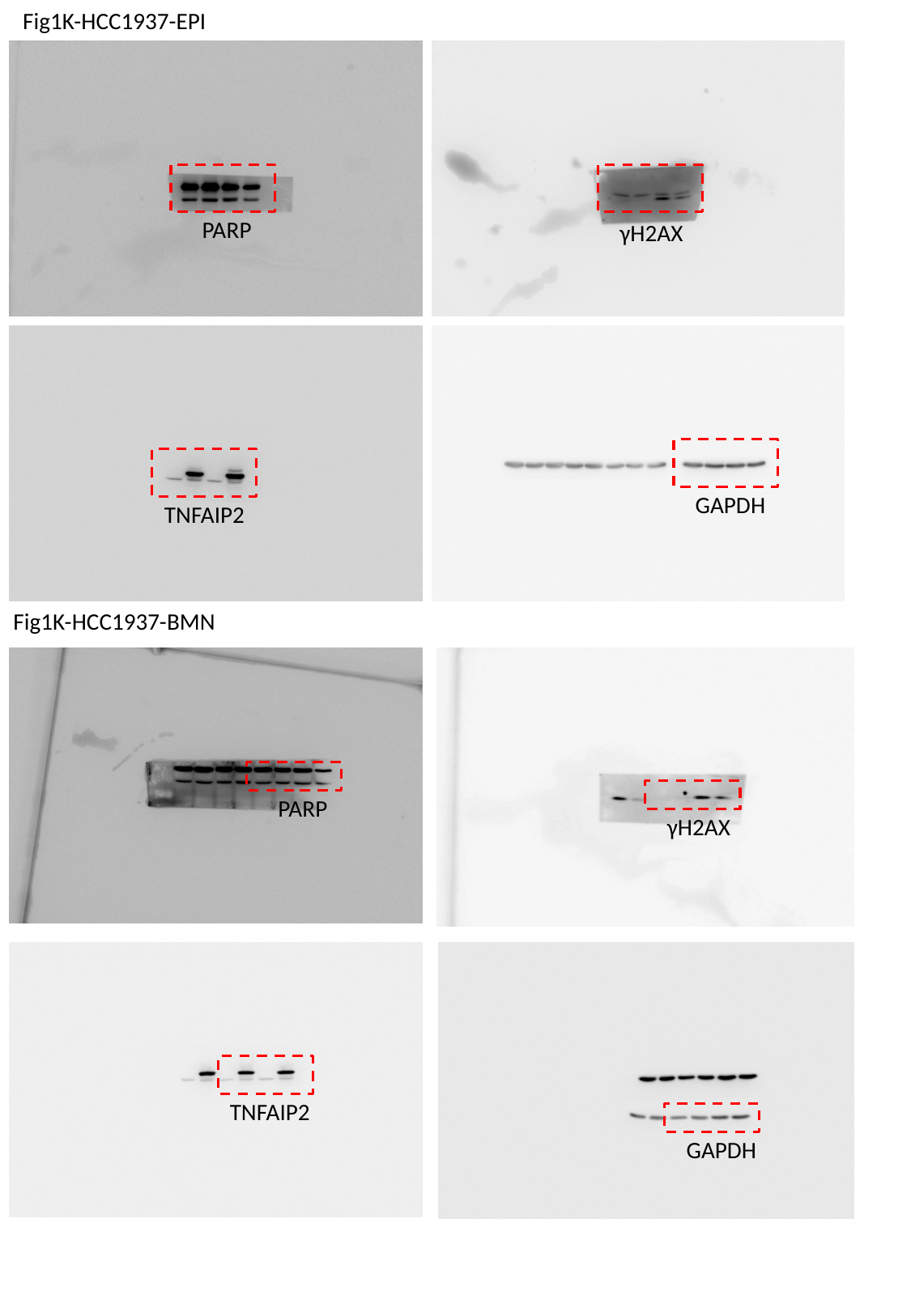

Fig1K-HCC1937-EPI
PARP
γH2AX
GAPDH
TNFAIP2
Fig1K-HCC1937-BMN
PARP
γH2AX
TNFAIP2
GAPDH

## Slide 4
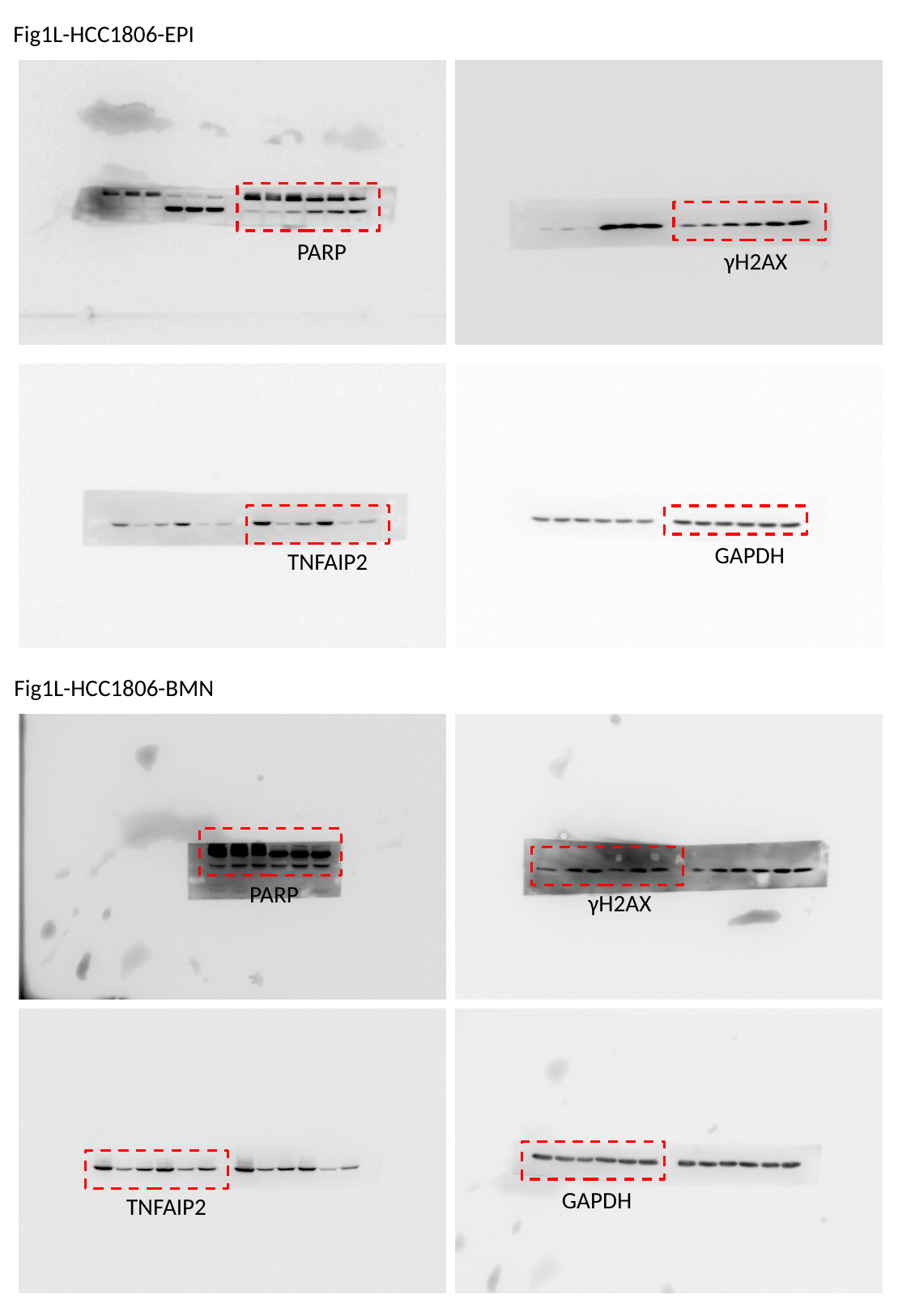

Fig1L-HCC1806-EPI
PARP
γH2AX
GAPDH
TNFAIP2
Fig1L-HCC1806-BMN
PARP
γH2AX
GAPDH
TNFAIP2

## Slide 5
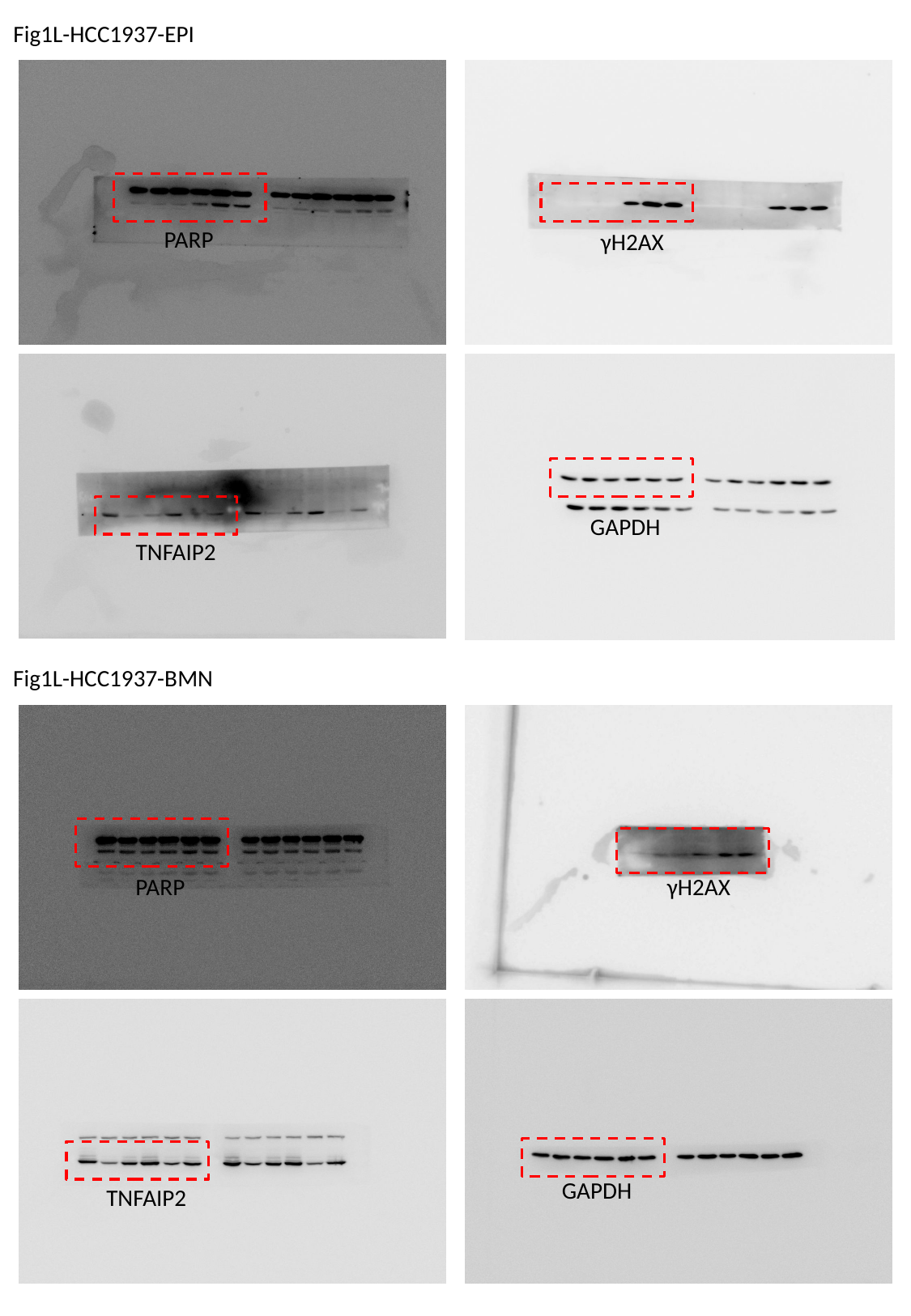

Fig1L-HCC1937-EPI
PARP
γH2AX
GAPDH
TNFAIP2
Fig1L-HCC1937-BMN
PARP
γH2AX
GAPDH
TNFAIP2
