# Supplementary material for: Integrin β4 promotes DNA damage-related drug resistance in triple-negative breast cancer via TNFAIP2/IQGAP1/RAC1
Source: eLife. 2023 Oct 3;12:RP88483. doi: 10.7554/eLife.88483 (PMC10547475; doi:10.7554/eLife.88483)
Supplement: Figure 1—figure supplement 1—source data 1. [file elife-88483-fig1-figsupp1-data1.pptx]

## Slide 1
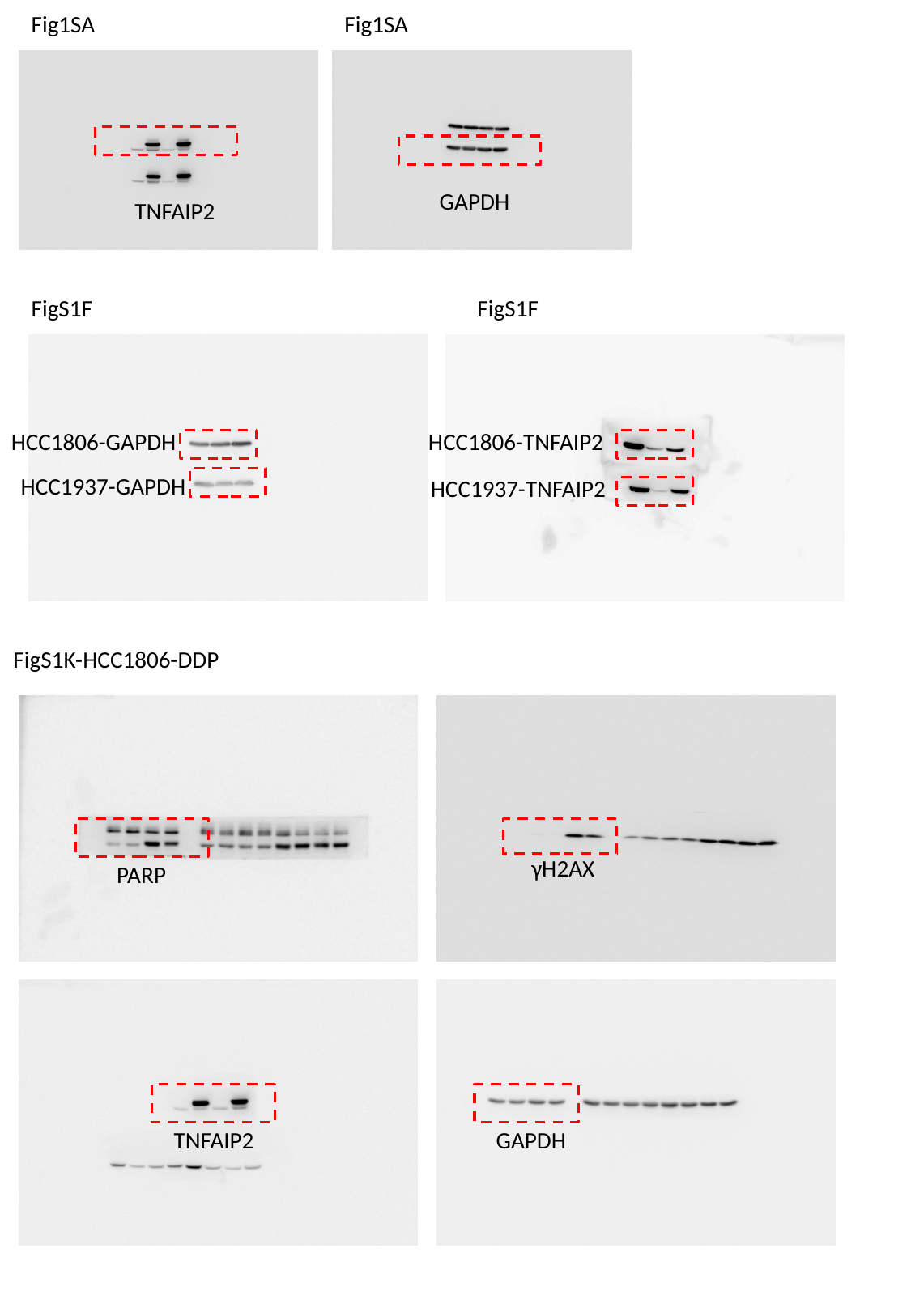

Fig1SA
Fig1SA
GAPDH
TNFAIP2
FigS1F
FigS1F
HCC1806-GAPDH
HCC1806-TNFAIP2
HCC1937-GAPDH
HCC1937-TNFAIP2
FigS1K-HCC1806-DDP
γH2AX
PARP
TNFAIP2
GAPDH

## Slide 2
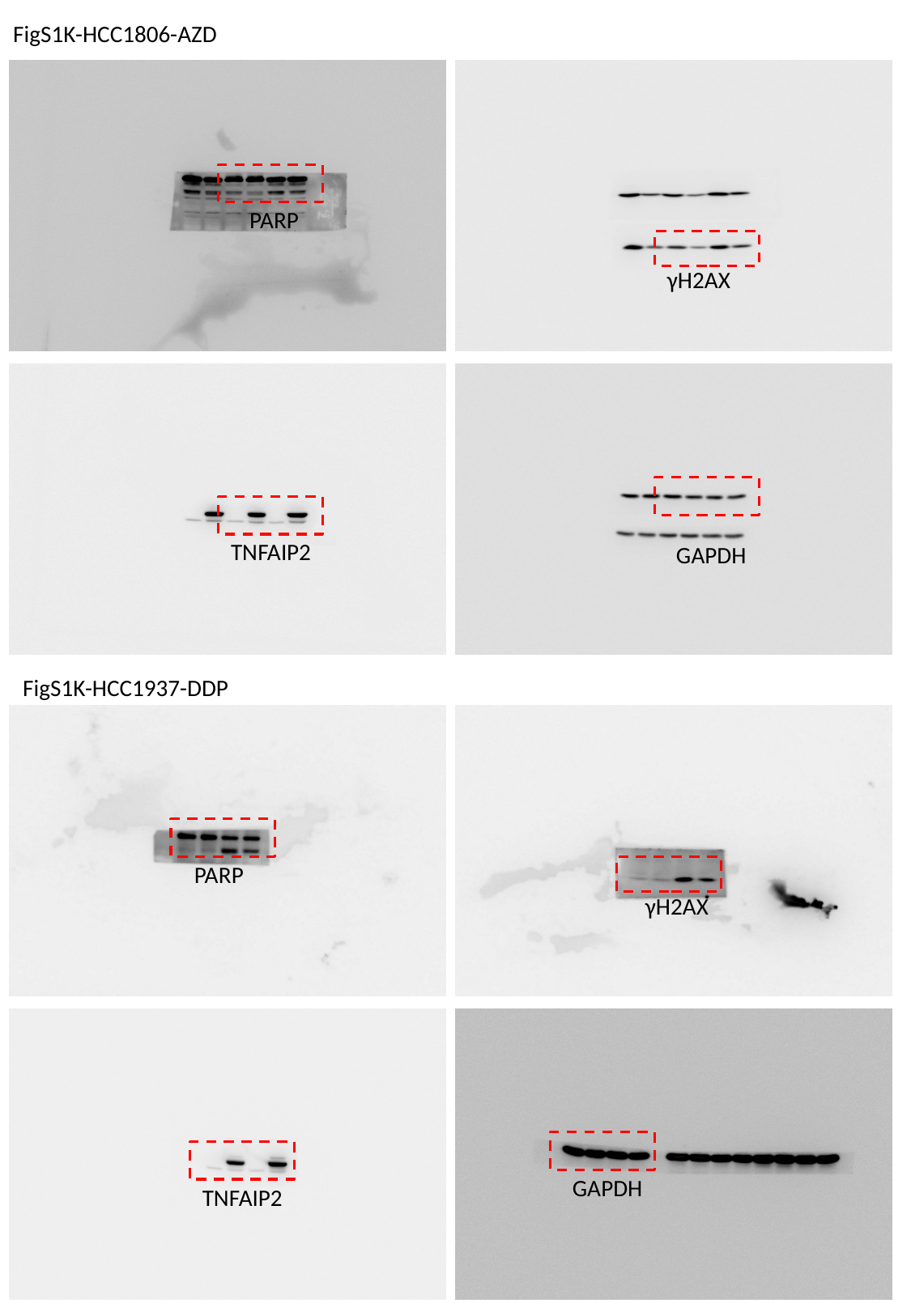

FigS1K-HCC1806-AZD
PARP
γH2AX
TNFAIP2
GAPDH
FigS1K-HCC1937-DDP
PARP
γH2AX
GAPDH
TNFAIP2

## Slide 3
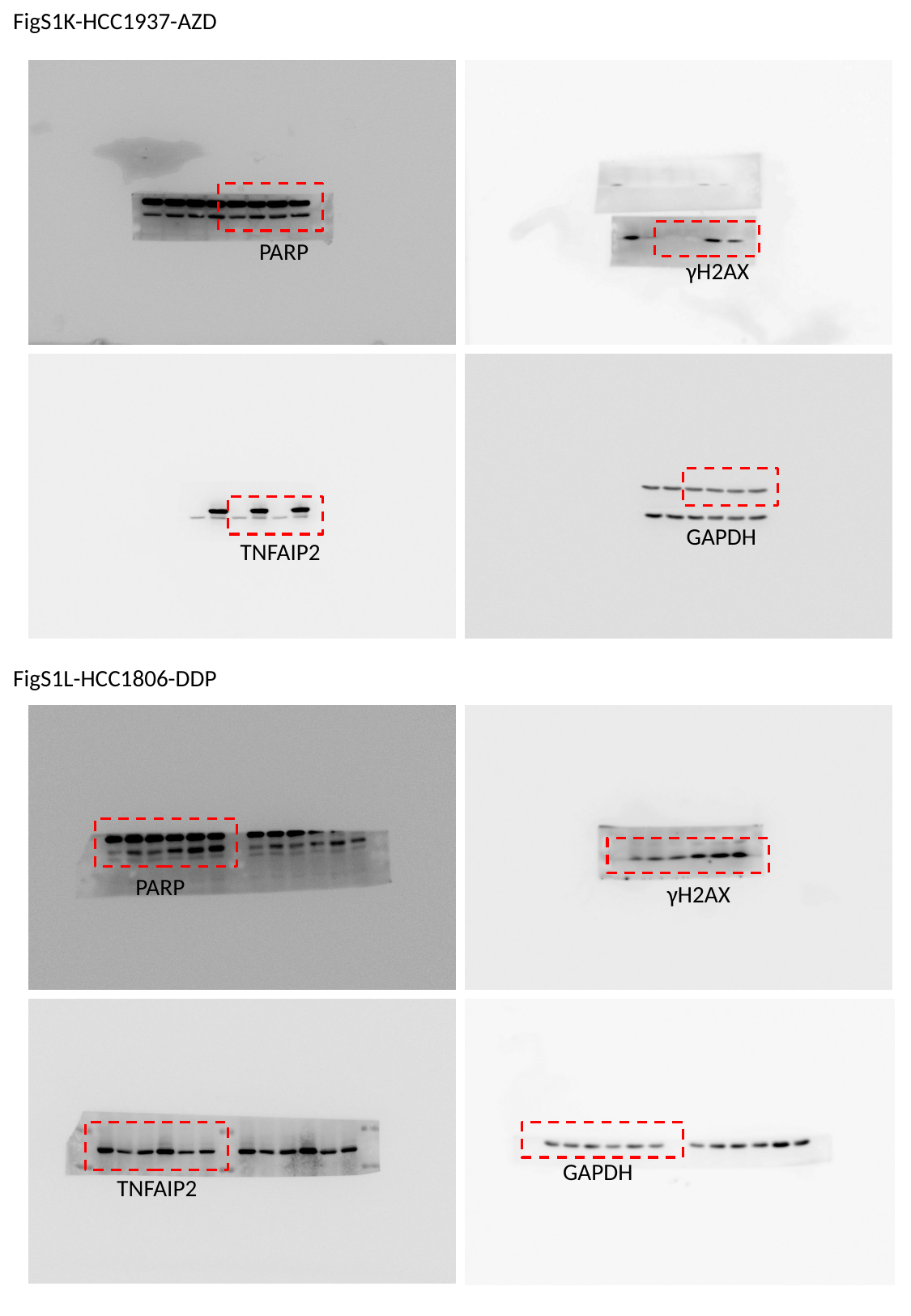

FigS1K-HCC1937-AZD
PARP
γH2AX
GAPDH
TNFAIP2
FigS1L-HCC1806-DDP
PARP
γH2AX
GAPDH
TNFAIP2

## Slide 4
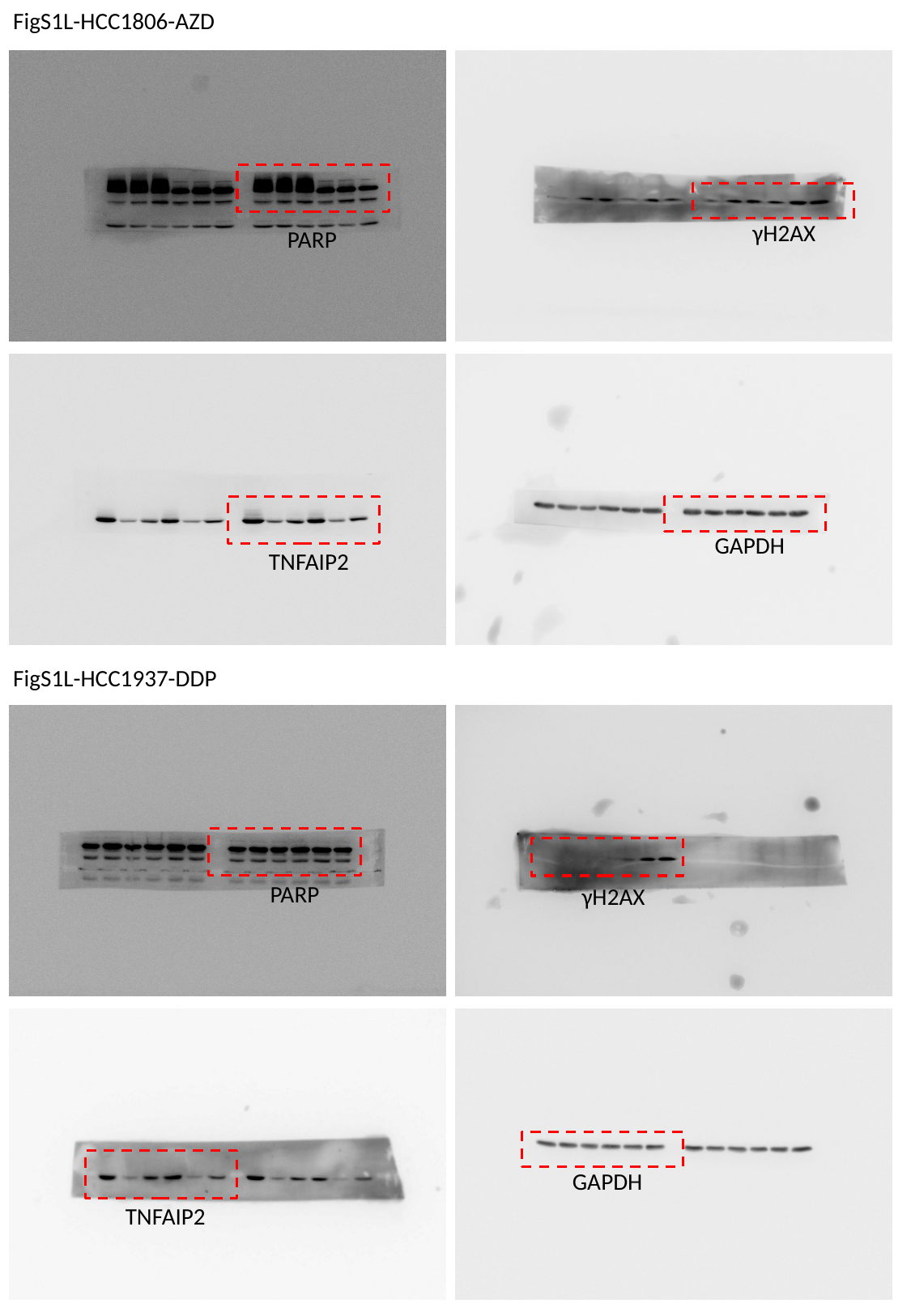

FigS1L-HCC1806-AZD
γH2AX
PARP
GAPDH
TNFAIP2
FigS1L-HCC1937-DDP
PARP
γH2AX
GAPDH
TNFAIP2

## Slide 5
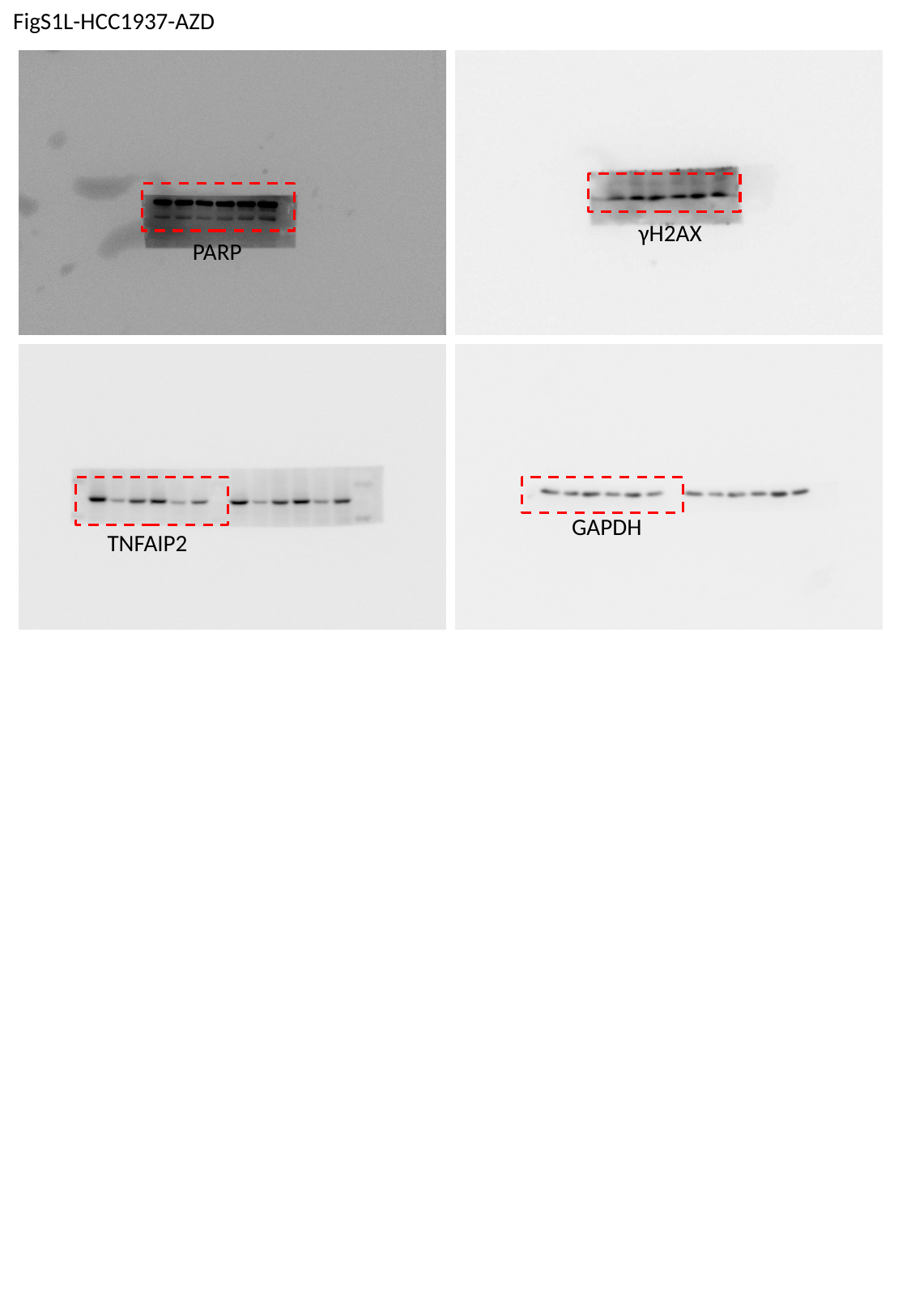

FigS1L-HCC1937-AZD
γH2AX
PARP
GAPDH
TNFAIP2
