# Supplementary figures and images for: Integrin β4 promotes DNA damage-related drug resistance in triple-negative breast cancer via TNFAIP2/IQGAP1/RAC1
Source: eLife. 2023 Oct 3;12:RP88483. doi: 10.7554/eLife.88483 (PMC10547475; doi:10.7554/eLife.88483)

## Slide 1
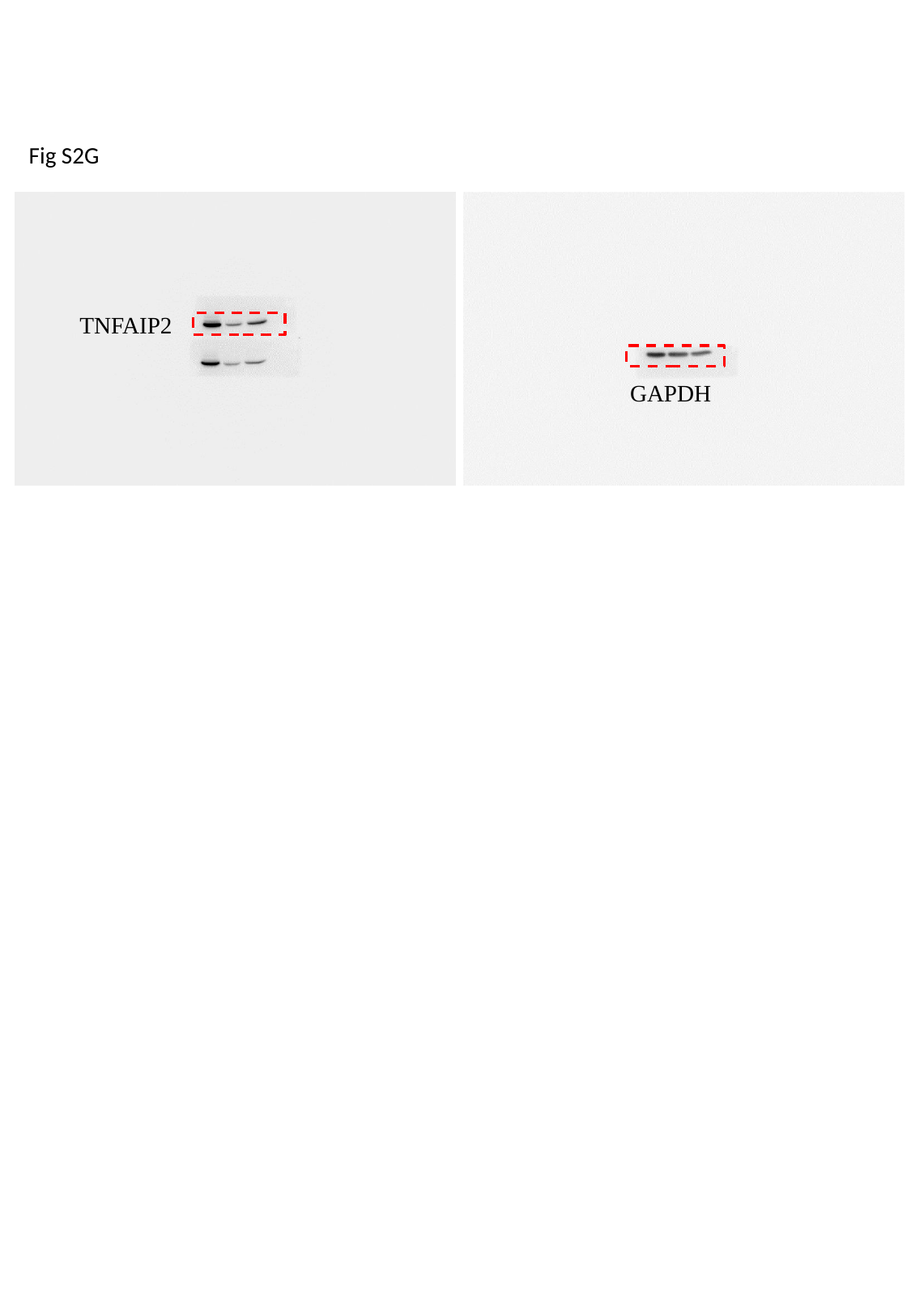

Fig S2G
TNFAIP2
GAPDH

Supplement: Figure 2—figure supplement 2—source data 1. [file elife-88483-fig2-figsupp2-data1.pptx]
