# Supplementary material for: Integrin β4 promotes DNA damage-related drug resistance in triple-negative breast cancer via TNFAIP2/IQGAP1/RAC1
Source: eLife. 2023 Oct 3;12:RP88483. doi: 10.7554/eLife.88483 (PMC10547475; doi:10.7554/eLife.88483)
Supplement: Figure 3—source data 1. [file elife-88483-fig3-data1.pptx]

## Slide 1
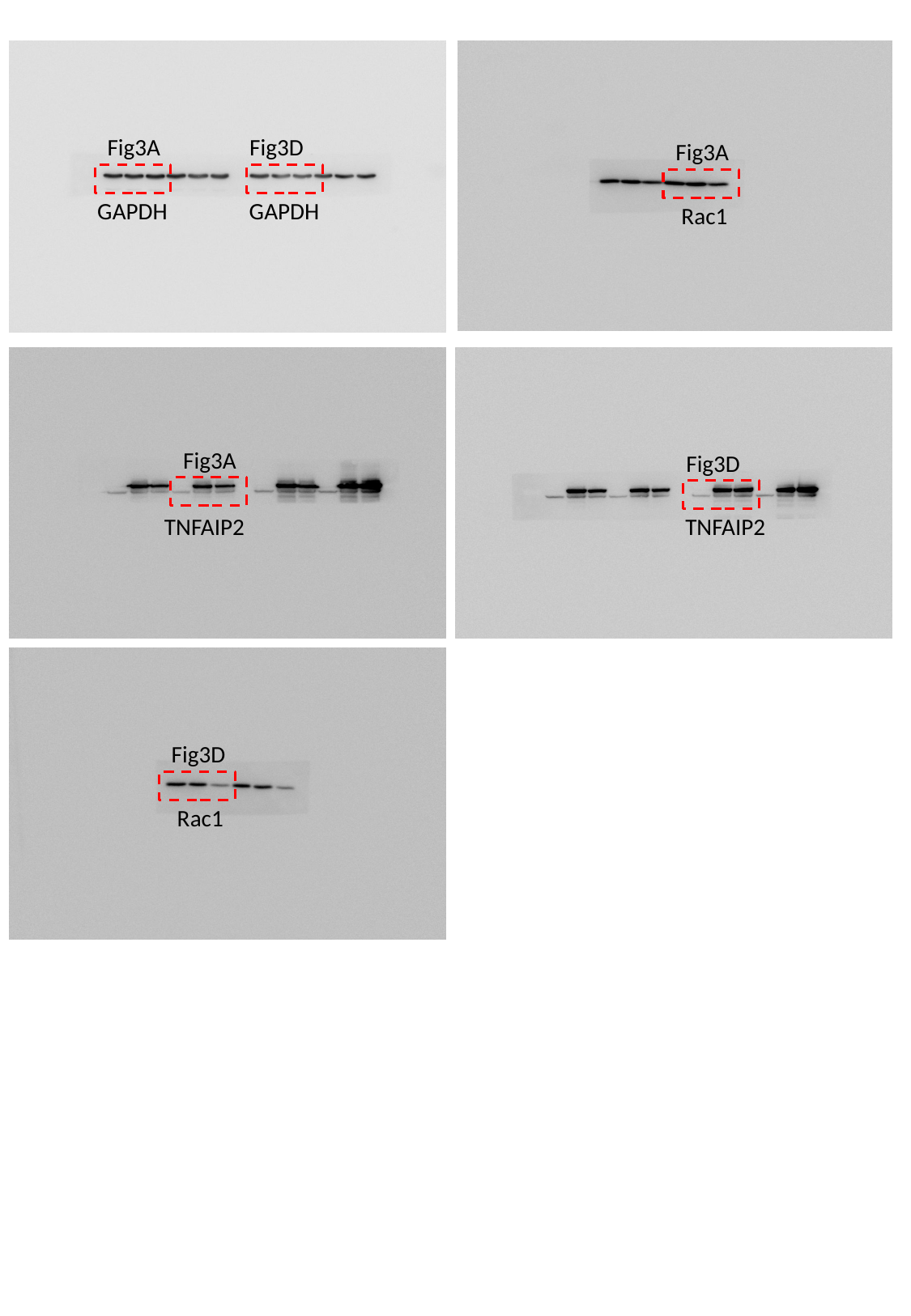

Fig3A
Fig3D
Fig3A
GAPDH
GAPDH
Rac1
Fig3A
Fig3D
TNFAIP2
TNFAIP2
Fig3D
Rac1

## Slide 2
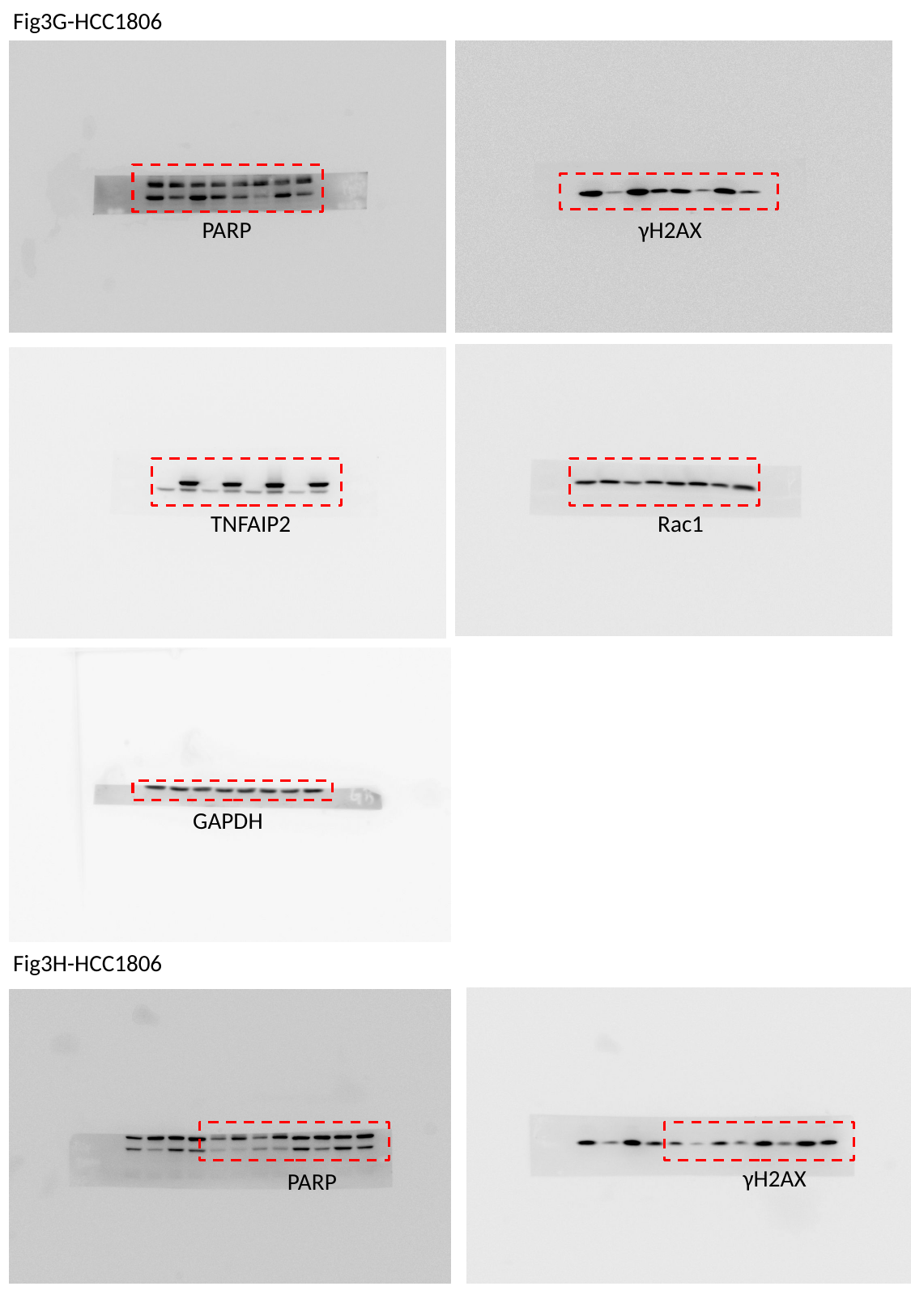

Fig3G-HCC1806
PARP
γH2AX
TNFAIP2
Rac1
GAPDH
Fig3H-HCC1806
γH2AX
PARP

## Slide 3
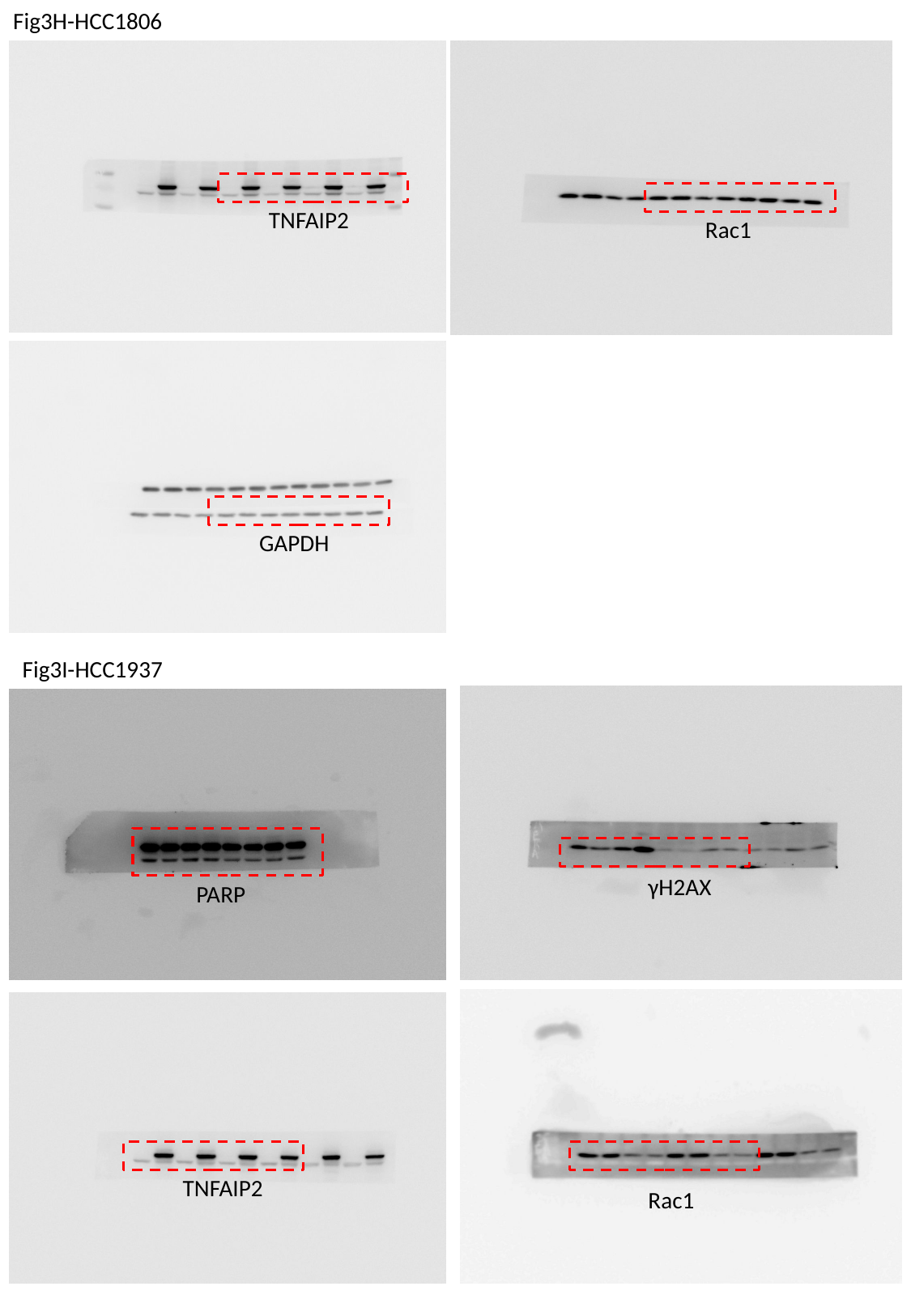

Fig3H-HCC1806
TNFAIP2
Rac1
GAPDH
Fig3I-HCC1937
γH2AX
PARP
TNFAIP2
Rac1

## Slide 4
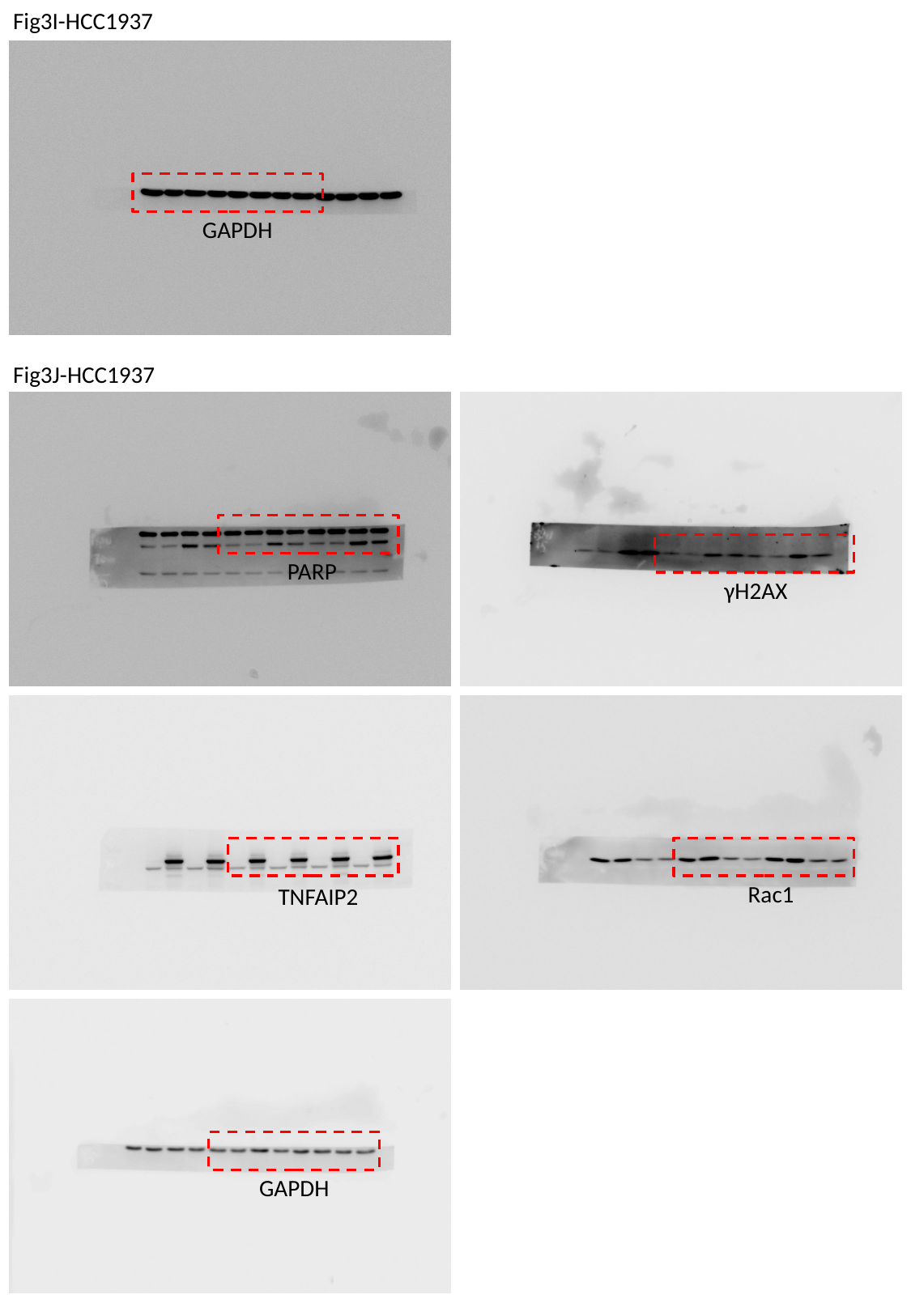

Fig3I-HCC1937
GAPDH
Fig3J-HCC1937
PARP
γH2AX
Rac1
TNFAIP2
GAPDH
