# Supplementary material for: Integrin β4 promotes DNA damage-related drug resistance in triple-negative breast cancer via TNFAIP2/IQGAP1/RAC1
Source: eLife. 2023 Oct 3;12:RP88483. doi: 10.7554/eLife.88483 (PMC10547475; doi:10.7554/eLife.88483)
Supplement: Figure 3—figure supplement 1—source data 1. [file elife-88483-fig3-figsupp1-data1.pptx]

## Slide 1
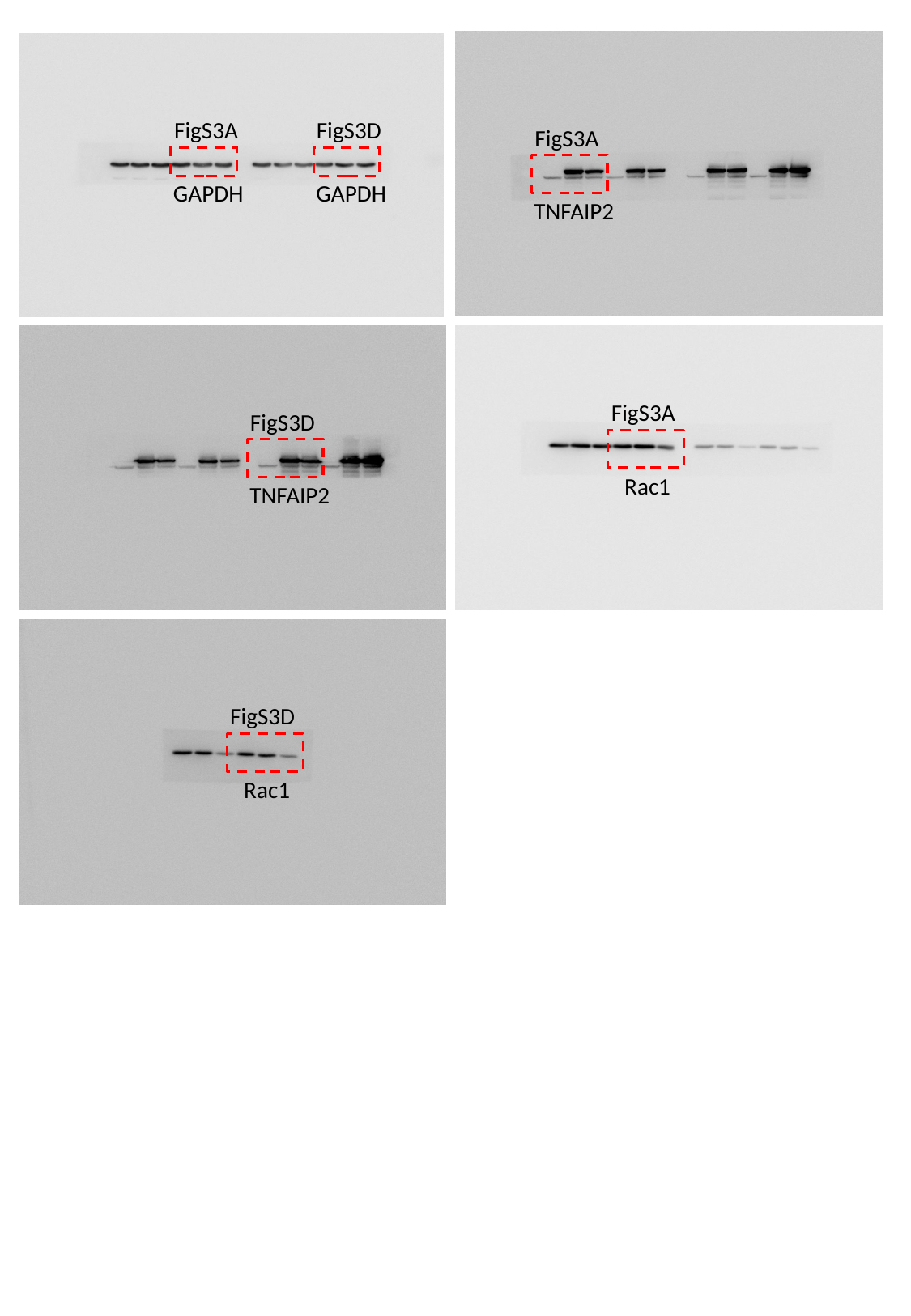

FigS3A
FigS3D
FigS3A
GAPDH
GAPDH
TNFAIP2
FigS3A
FigS3D
Rac1
TNFAIP2
FigS3D
Rac1

## Slide 2
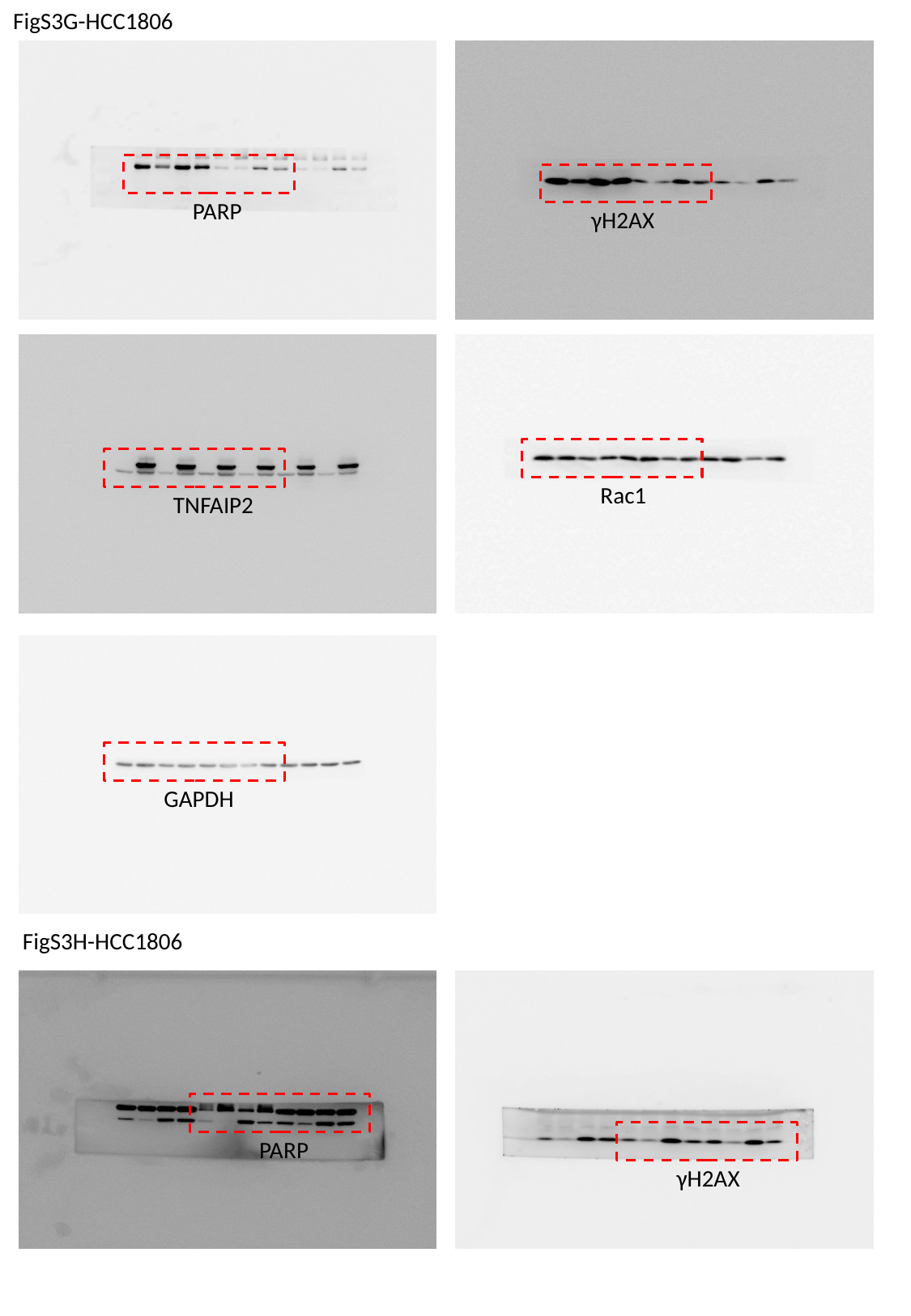

FigS3G-HCC1806
PARP
γH2AX
Rac1
TNFAIP2
GAPDH
FigS3H-HCC1806
PARP
γH2AX

## Slide 3
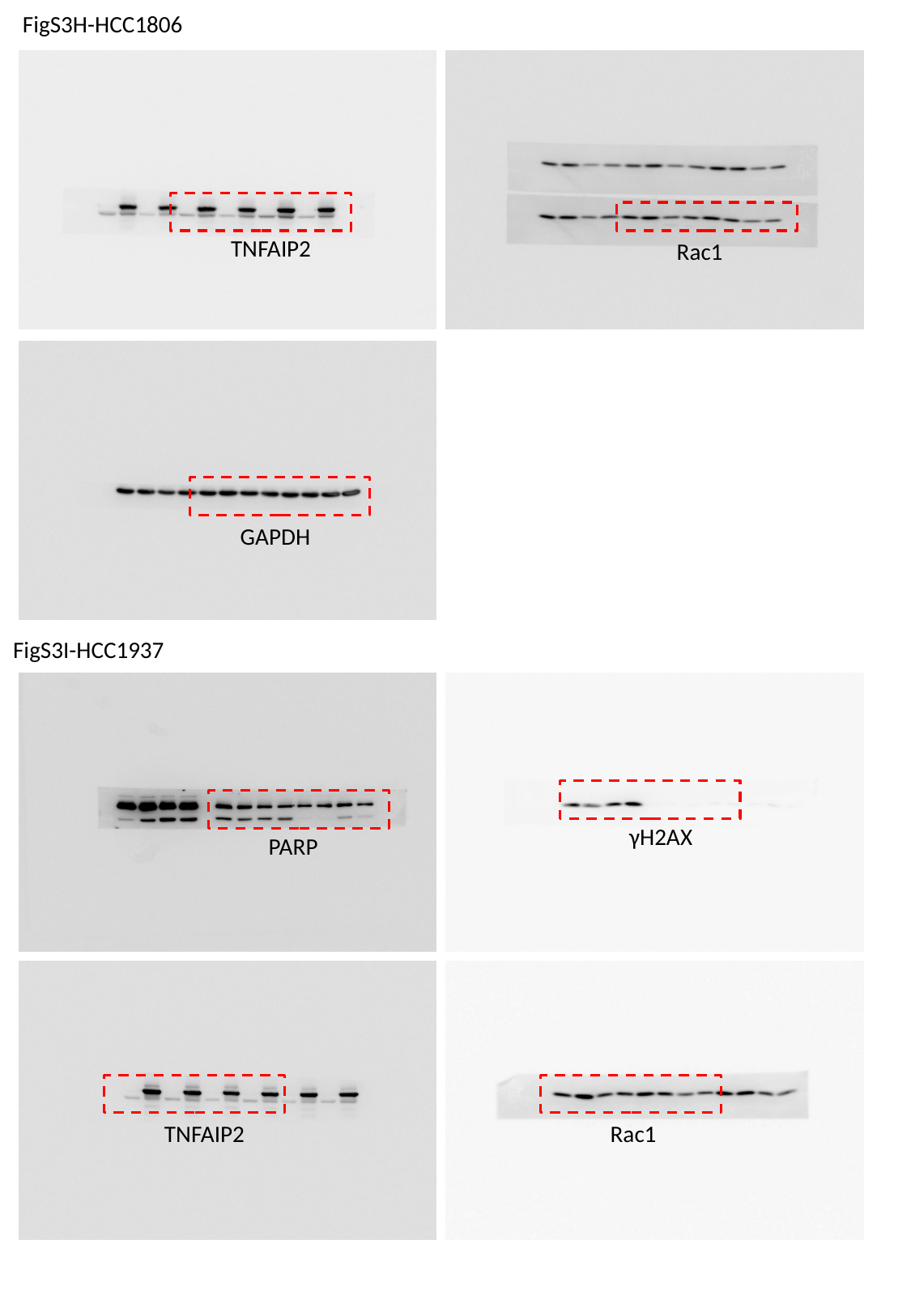

FigS3H-HCC1806
TNFAIP2
Rac1
GAPDH
FigS3I-HCC1937
γH2AX
PARP
TNFAIP2
Rac1

## Slide 4
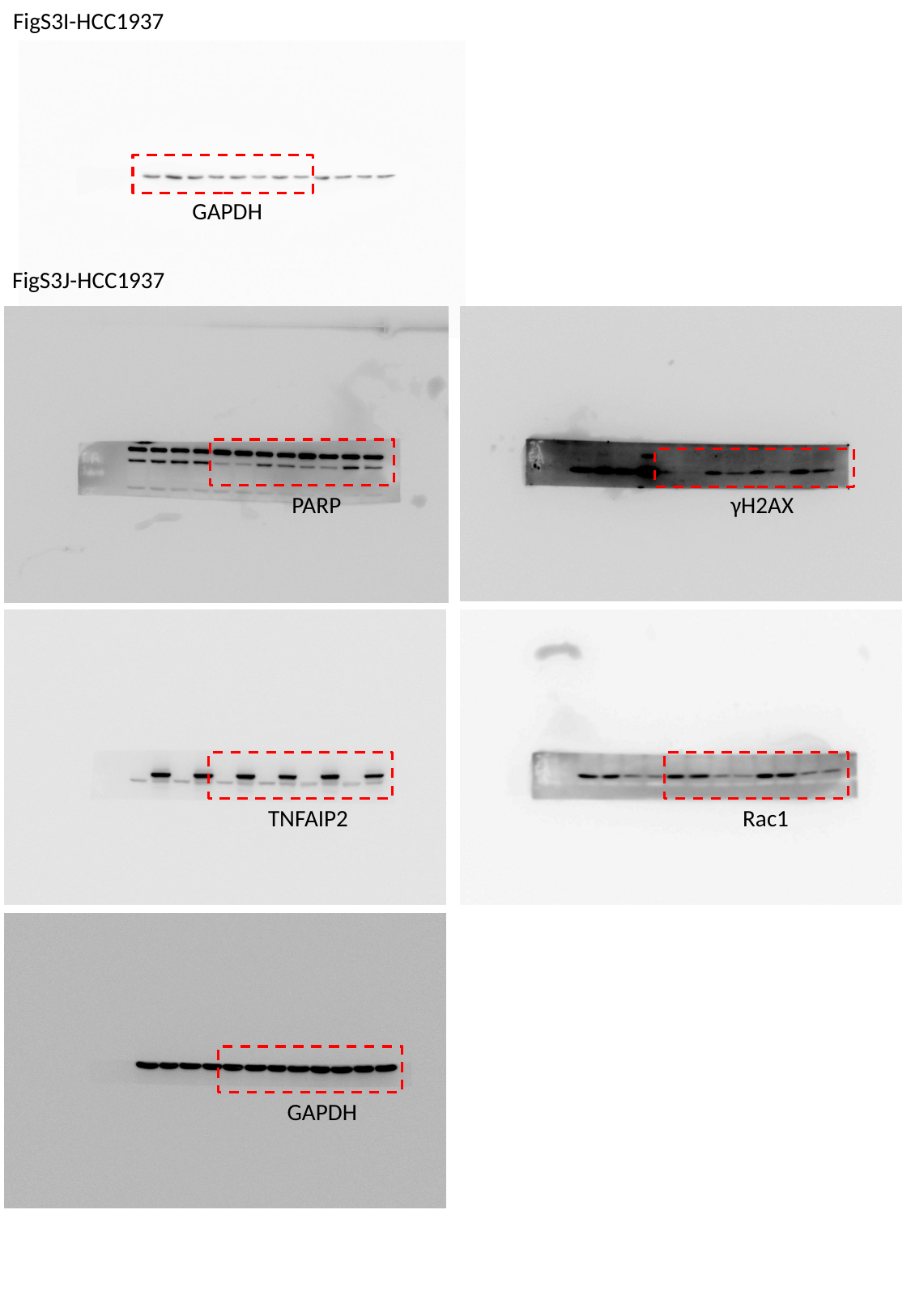

FigS3I-HCC1937
GAPDH
FigS3J-HCC1937
PARP
γH2AX
TNFAIP2
Rac1
GAPDH
