# Supplementary material for: Integrin β4 promotes DNA damage-related drug resistance in triple-negative breast cancer via TNFAIP2/IQGAP1/RAC1
Source: eLife. 2023 Oct 3;12:RP88483. doi: 10.7554/eLife.88483 (PMC10547475; doi:10.7554/eLife.88483)
Supplement: Figure 4—source data 1. [file elife-88483-fig4-data1.pptx]

## Slide 1
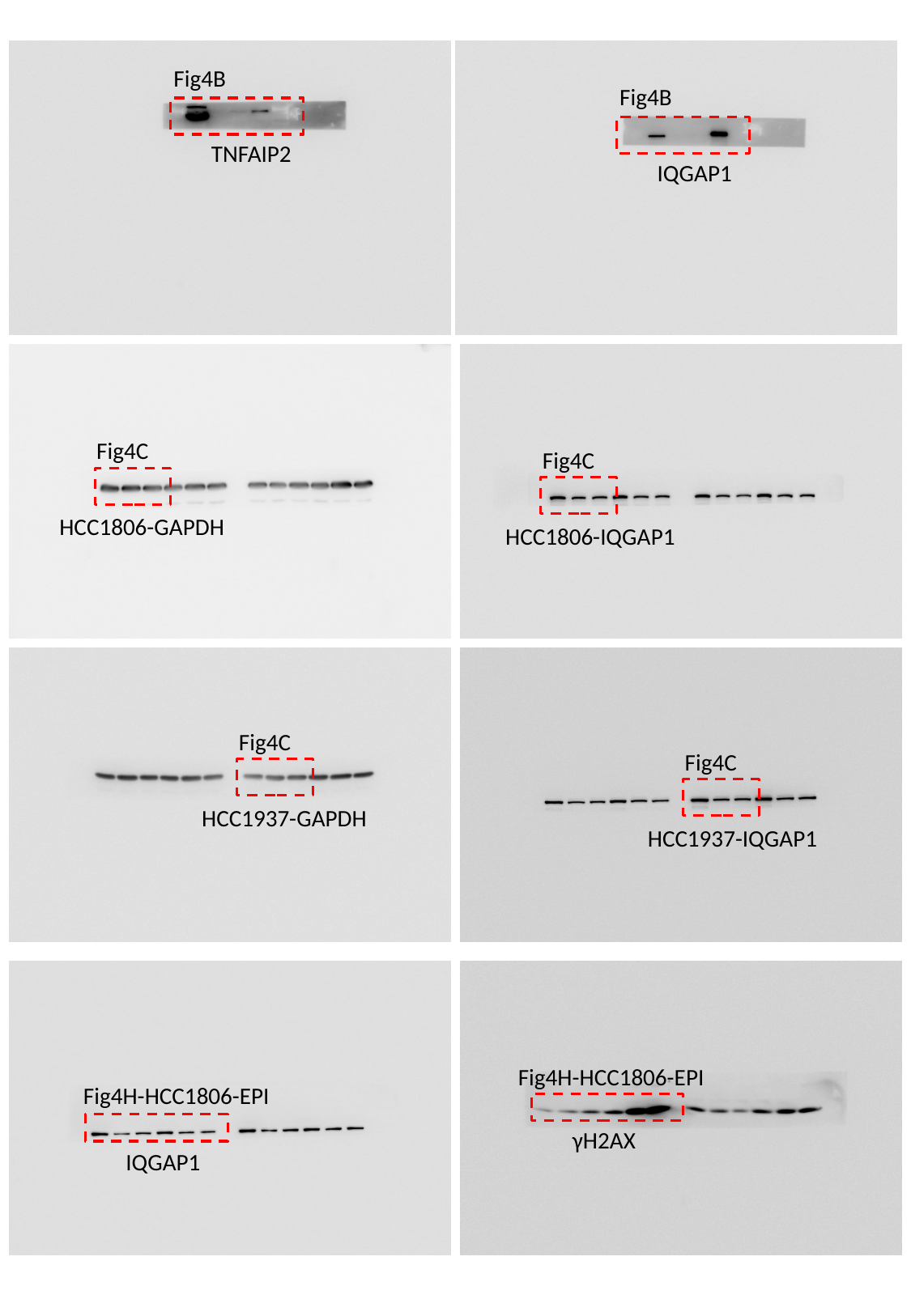

Fig4B
Fig4B
TNFAIP2
IQGAP1
Fig4C
Fig4C
HCC1806-GAPDH
HCC1806-IQGAP1
Fig4C
Fig4C
HCC1937-GAPDH
HCC1937-IQGAP1
Fig4H-HCC1806-EPI
Fig4H-HCC1806-EPI
γH2AX
IQGAP1

## Slide 2
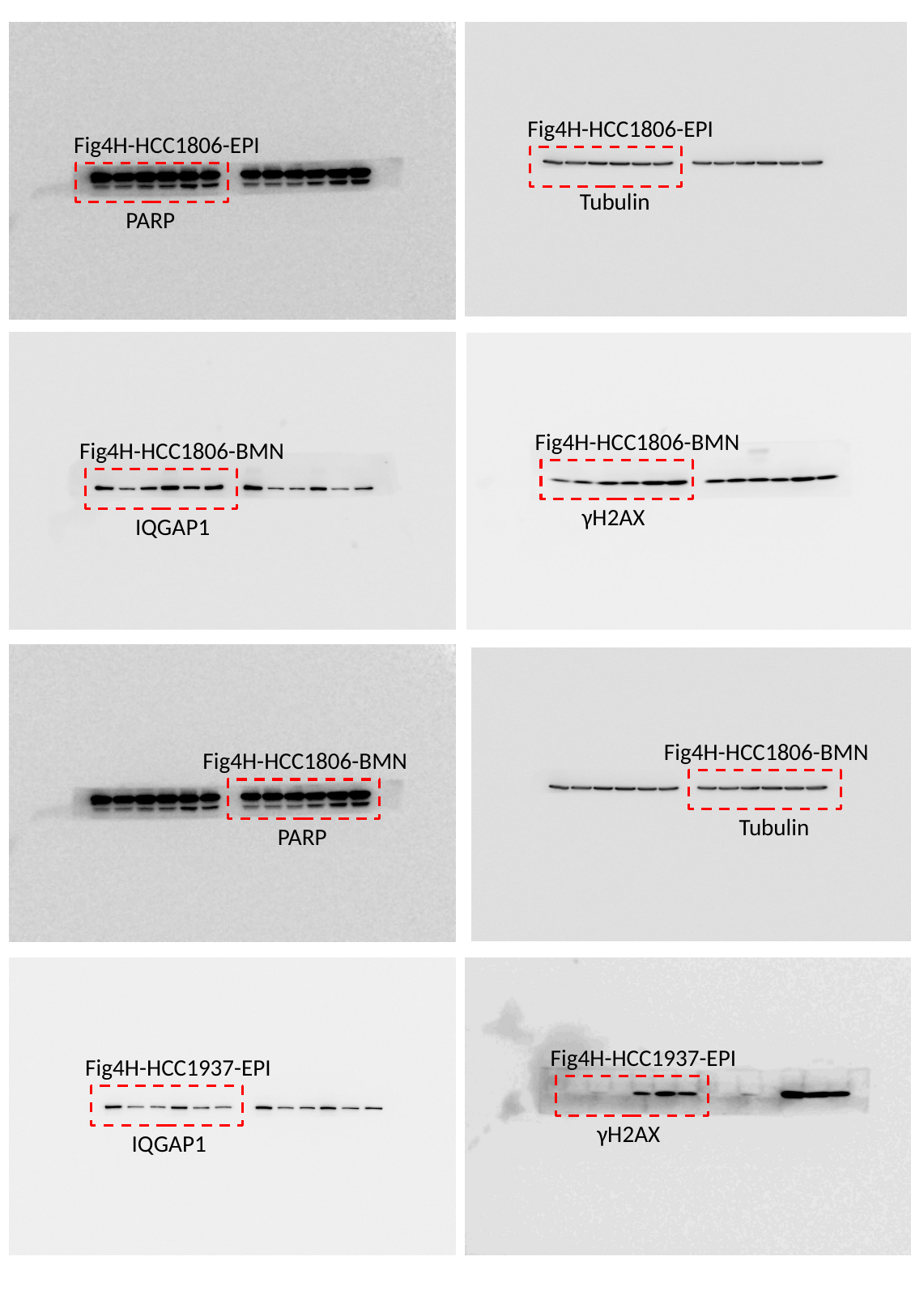

Fig4H-HCC1806-EPI
Fig4H-HCC1806-EPI
Tubulin
PARP
Fig4H-HCC1806-BMN
Fig4H-HCC1806-BMN
γH2AX
IQGAP1
Fig4H-HCC1806-BMN
Fig4H-HCC1806-BMN
Tubulin
PARP
Fig4H-HCC1937-EPI
Fig4H-HCC1937-EPI
γH2AX
IQGAP1

## Slide 3
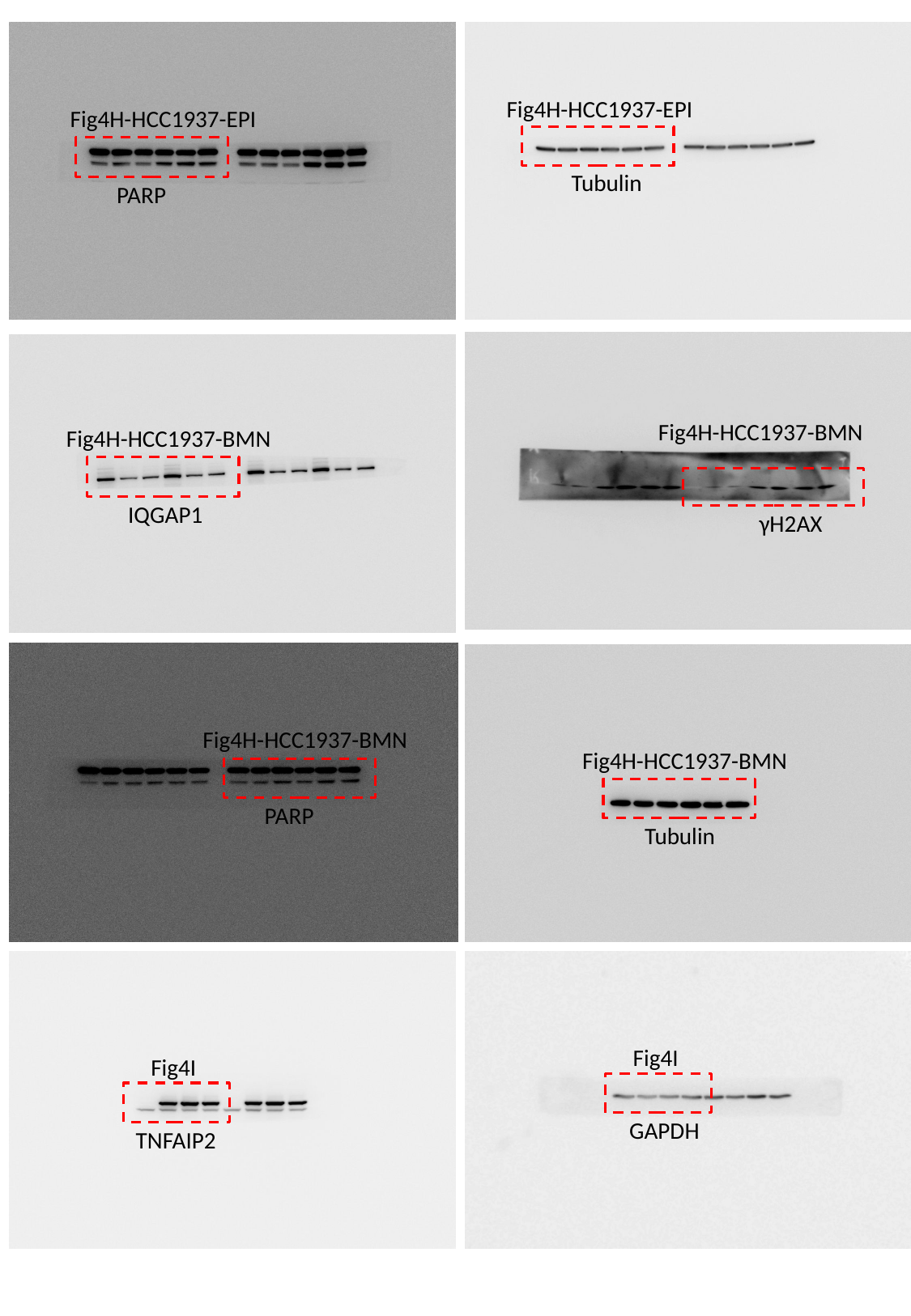

Fig4H-HCC1937-EPI
Fig4H-HCC1937-EPI
Tubulin
PARP
Fig4H-HCC1937-BMN
Fig4H-HCC1937-BMN
IQGAP1
γH2AX
Fig4H-HCC1937-BMN
Fig4H-HCC1937-BMN
PARP
Tubulin
Fig4I
Fig4I
GAPDH
TNFAIP2

## Slide 4
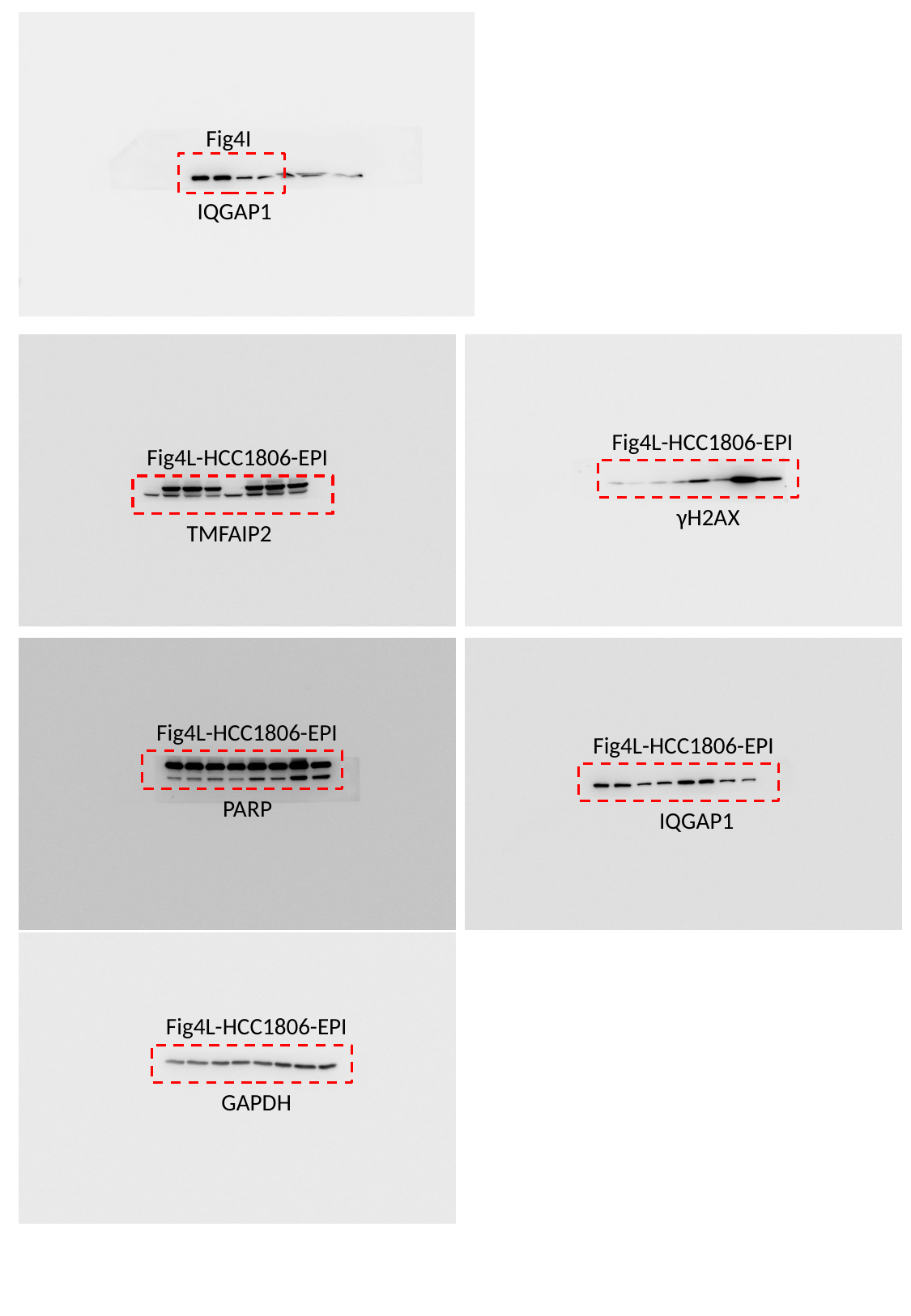

Fig4I
IQGAP1
Fig4L-HCC1806-EPI
Fig4L-HCC1806-EPI
γH2AX
TMFAIP2
Fig4L-HCC1806-EPI
Fig4L-HCC1806-EPI
PARP
IQGAP1
Fig4L-HCC1806-EPI
GAPDH

## Slide 5
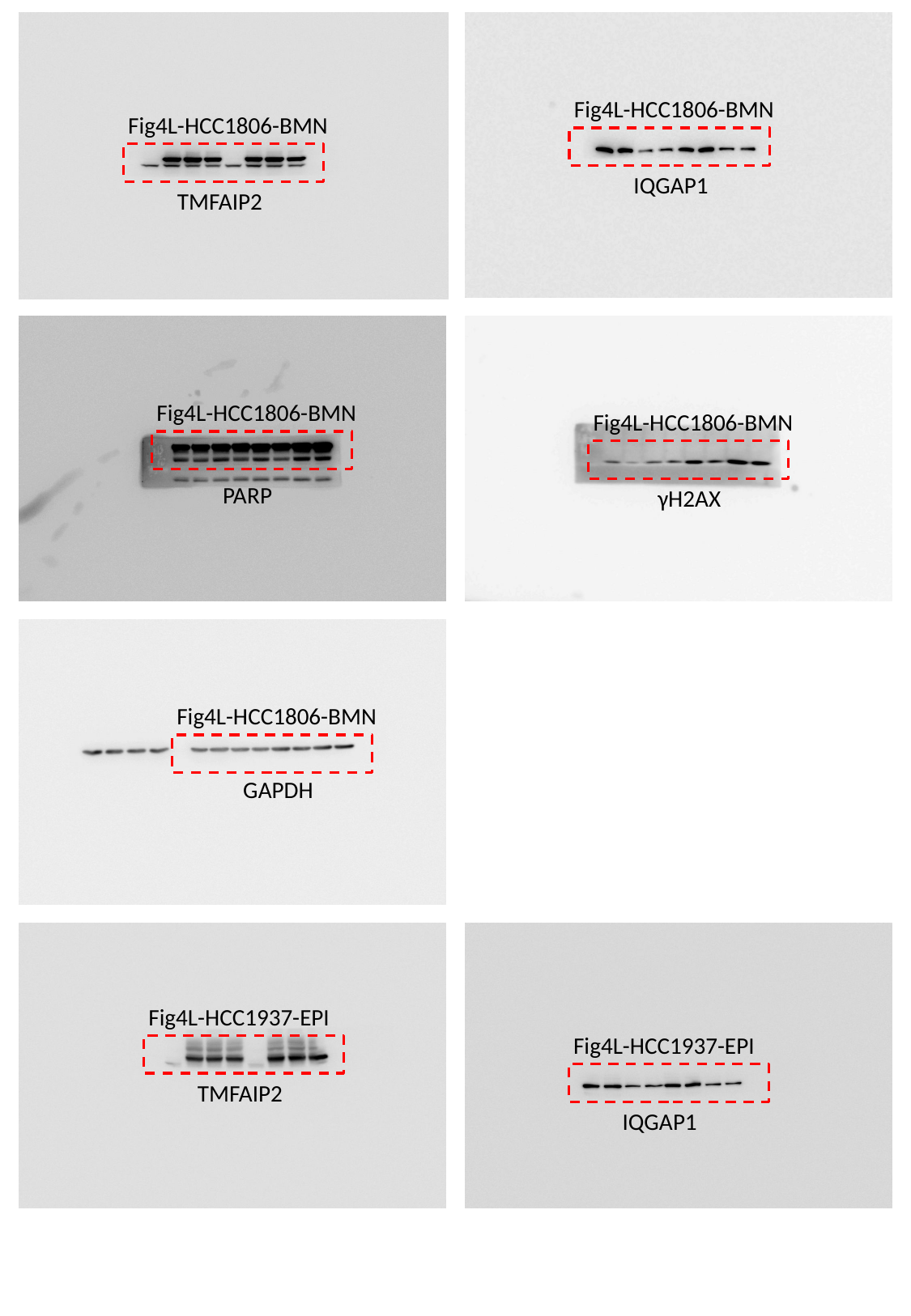

Fig4L-HCC1806-BMN
Fig4L-HCC1806-BMN
IQGAP1
TMFAIP2
Fig4L-HCC1806-BMN
Fig4L-HCC1806-BMN
PARP
γH2AX
Fig4L-HCC1806-BMN
GAPDH
Fig4L-HCC1937-EPI
Fig4L-HCC1937-EPI
TMFAIP2
IQGAP1

## Slide 6
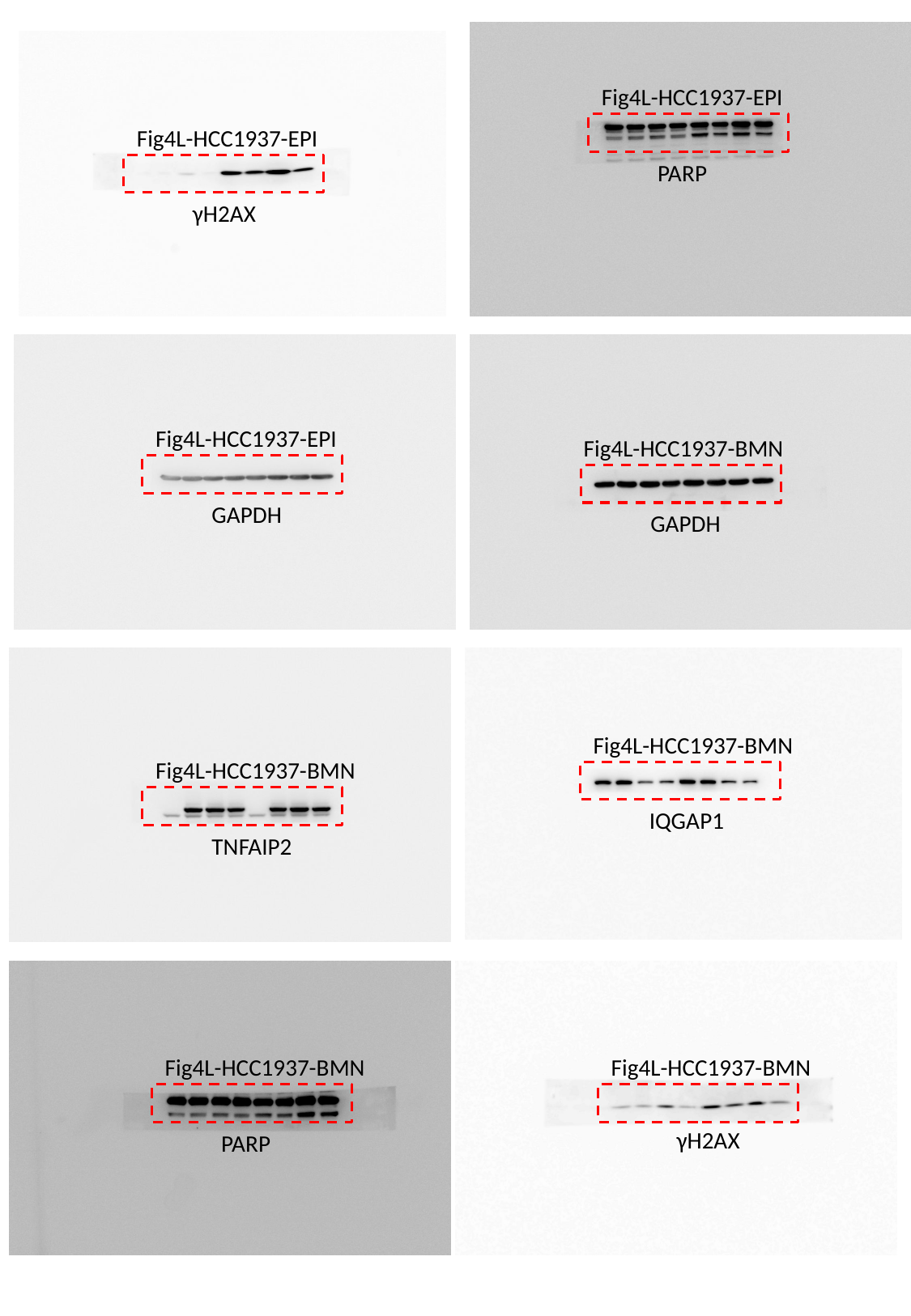

Fig4L-HCC1937-EPI
Fig4L-HCC1937-EPI
PARP
γH2AX
Fig4L-HCC1937-EPI
Fig4L-HCC1937-BMN
GAPDH
GAPDH
Fig4L-HCC1937-BMN
Fig4L-HCC1937-BMN
IQGAP1
TNFAIP2
Fig4L-HCC1937-BMN
Fig4L-HCC1937-BMN
γH2AX
PARP

## Slide 7
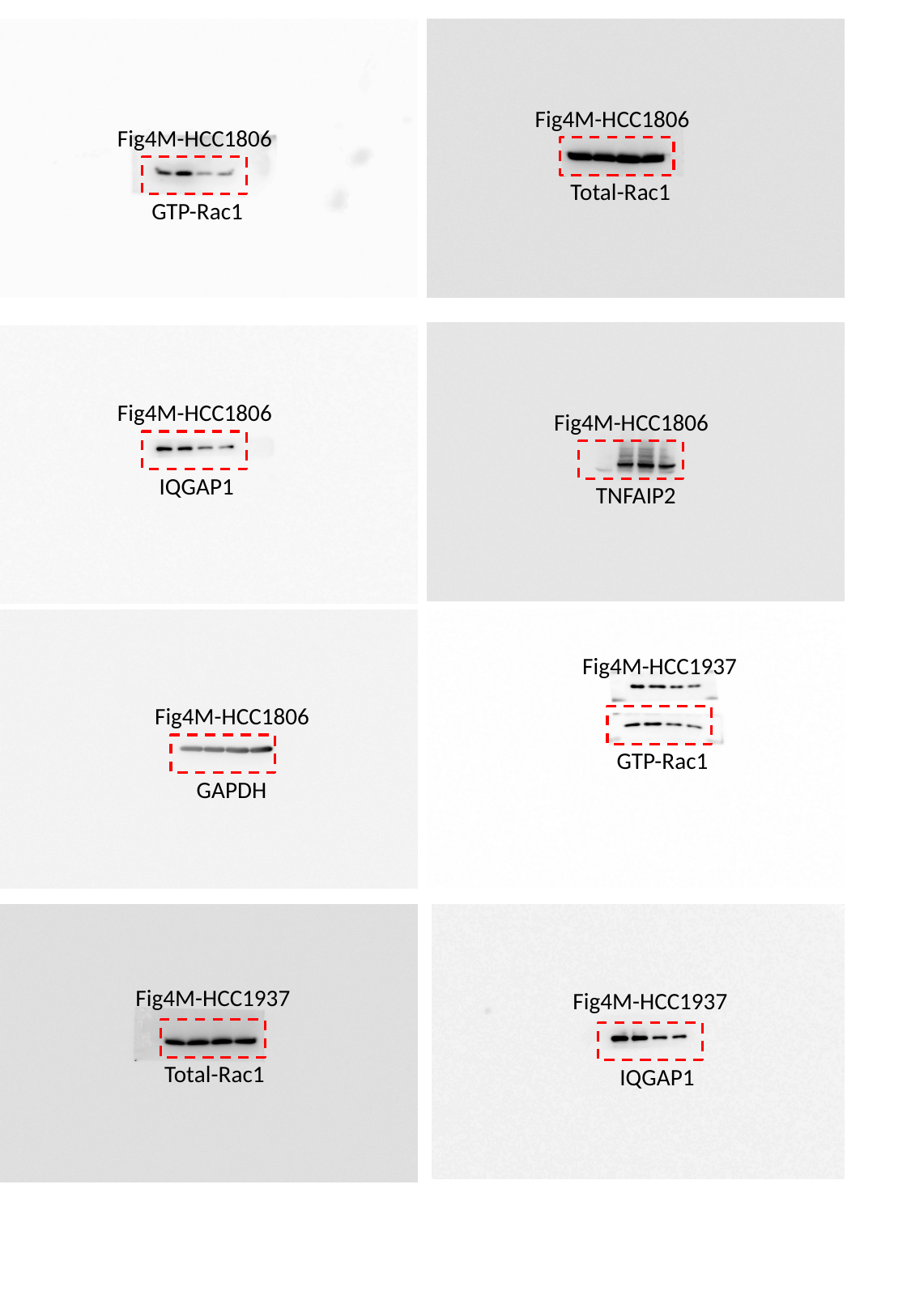

Fig4M-HCC1806
Fig4M-HCC1806
Total-Rac1
GTP-Rac1
Fig4M-HCC1806
Fig4M-HCC1806
IQGAP1
TNFAIP2
Fig4M-HCC1937
Fig4M-HCC1806
GTP-Rac1
GAPDH
Fig4M-HCC1937
Fig4M-HCC1937
Total-Rac1
IQGAP1

## Slide 8
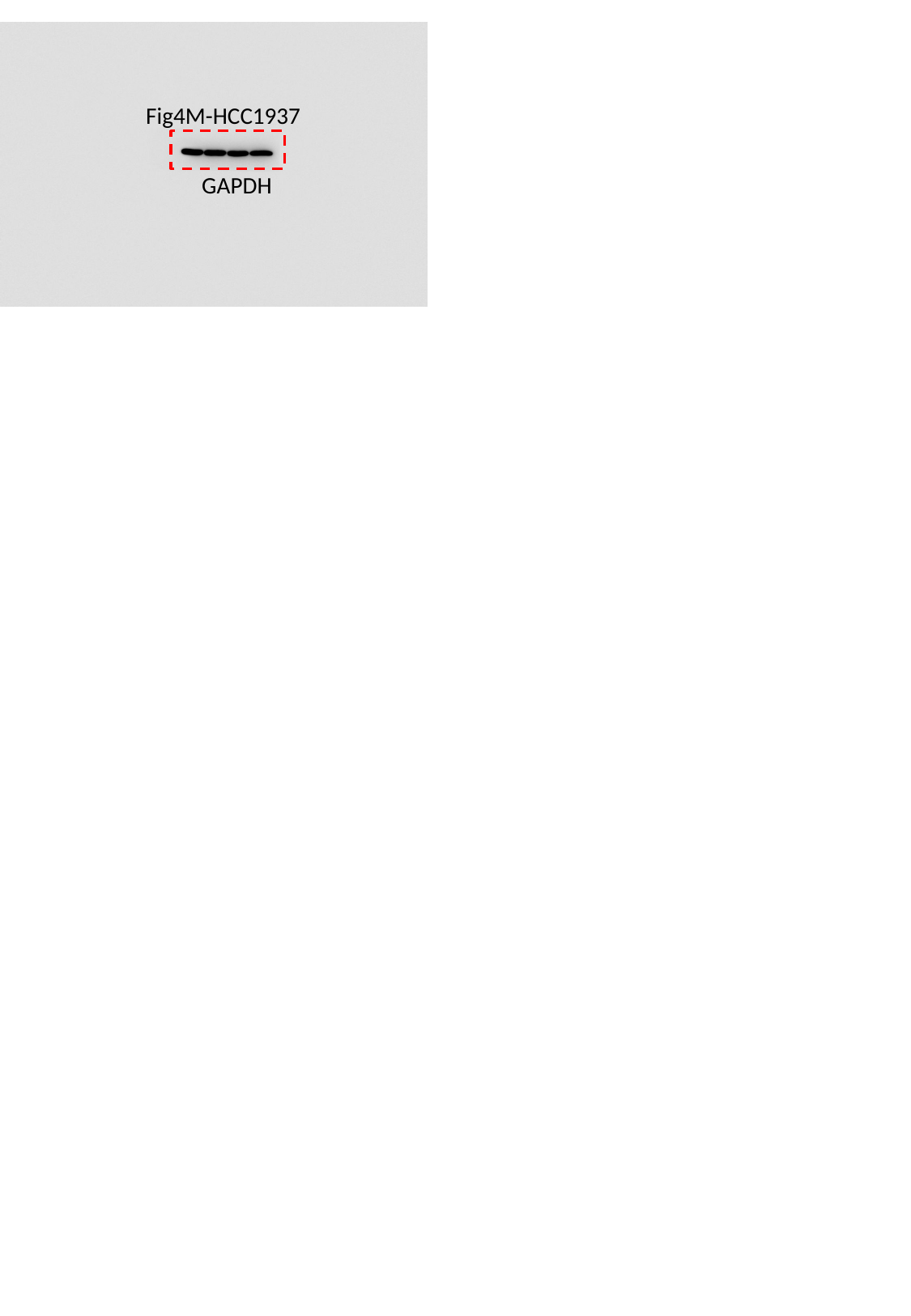

Fig4M-HCC1937
GAPDH
