# Supplementary material for: Integrin β4 promotes DNA damage-related drug resistance in triple-negative breast cancer via TNFAIP2/IQGAP1/RAC1
Source: eLife. 2023 Oct 3;12:RP88483. doi: 10.7554/eLife.88483 (PMC10547475; doi:10.7554/eLife.88483)
Supplement: Figure 4—figure supplement 1—source data 1. [file elife-88483-fig4-figsupp1-data1.pptx]

## Slide 1
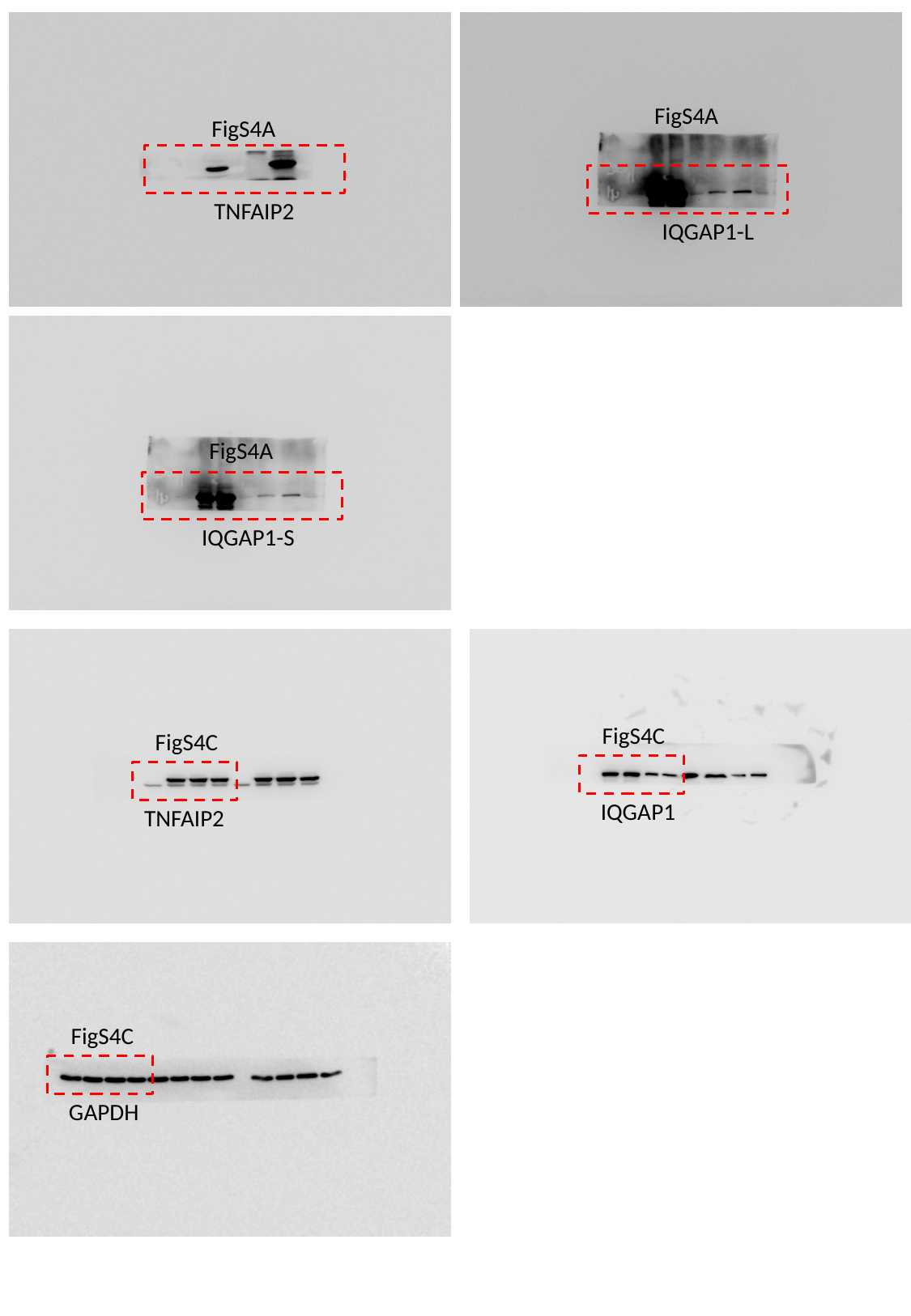

FigS4A
FigS4A
TNFAIP2
IQGAP1-L
FigS4A
IQGAP1-S
FigS4C
FigS4C
IQGAP1
TNFAIP2
FigS4C
GAPDH

## Slide 2
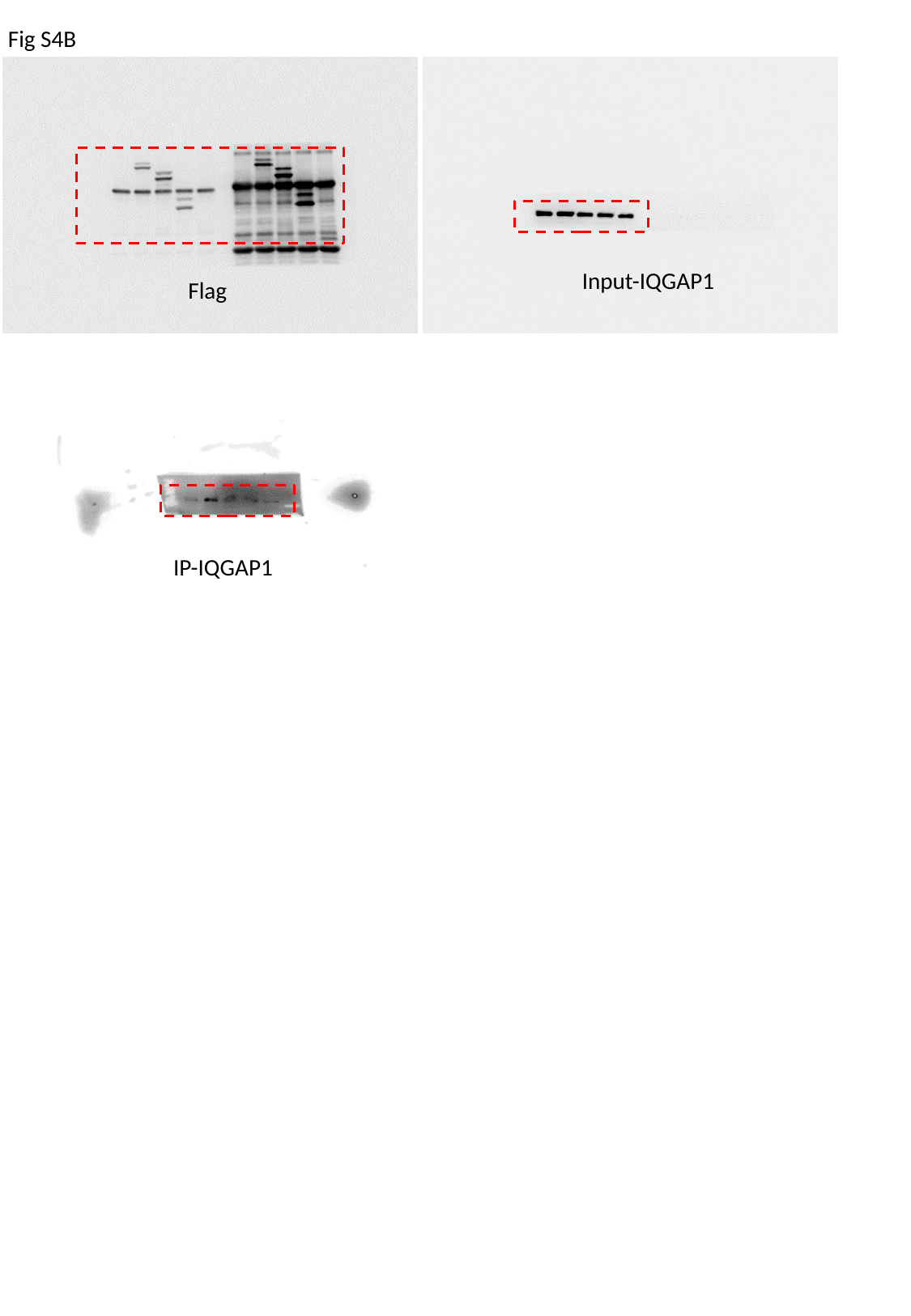

Fig S4B
Input-IQGAP1
Flag
IP-IQGAP1
