# Supplementary material for: Integrin β4 promotes DNA damage-related drug resistance in triple-negative breast cancer via TNFAIP2/IQGAP1/RAC1
Source: eLife. 2023 Oct 3;12:RP88483. doi: 10.7554/eLife.88483 (PMC10547475; doi:10.7554/eLife.88483)
Supplement: Figure 5—source data 1. [file elife-88483-fig5-data1.pptx]

## Slide 1
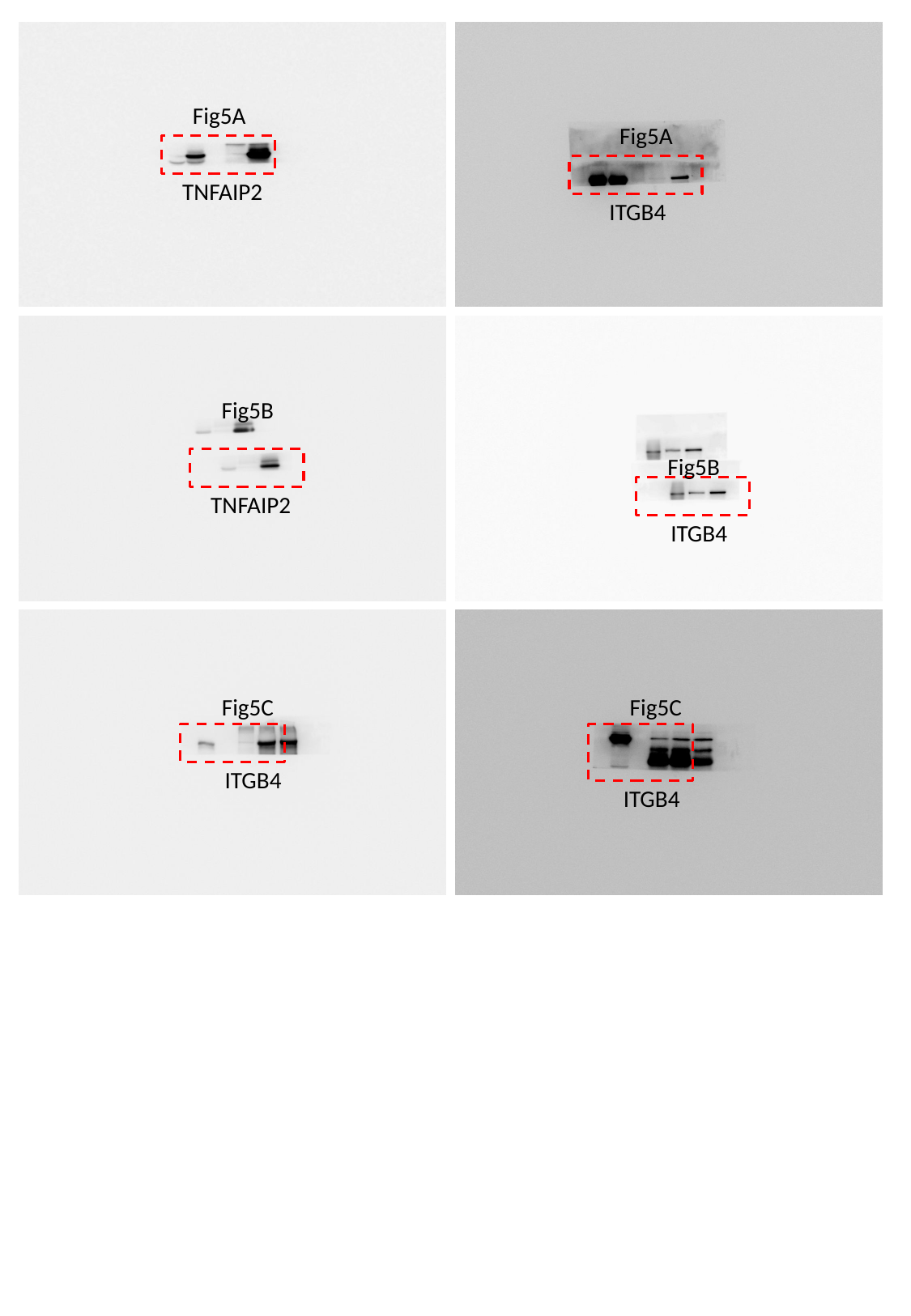

Fig5A
Fig5A
TNFAIP2
ITGB4
Fig5B
Fig5B
TNFAIP2
ITGB4
Fig5C
Fig5C
ITGB4
ITGB4

## Slide 2
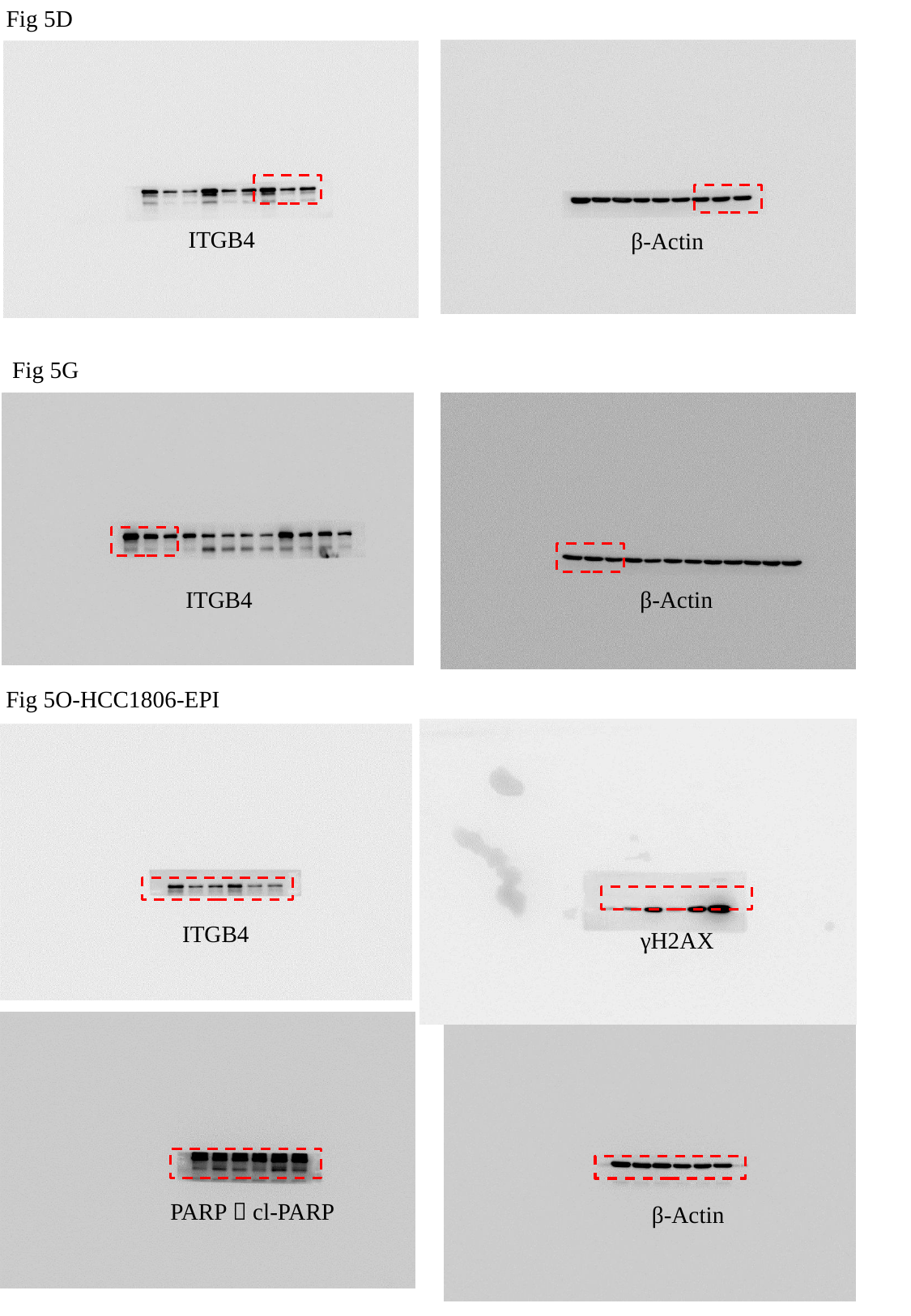

Fig 5D
ITGB4
β-Actin
Fig 5G
ITGB4
β-Actin
Fig 5O-HCC1806-EPI
ITGB4
γH2AX
PARP，cl-PARP
β-Actin

## Slide 3
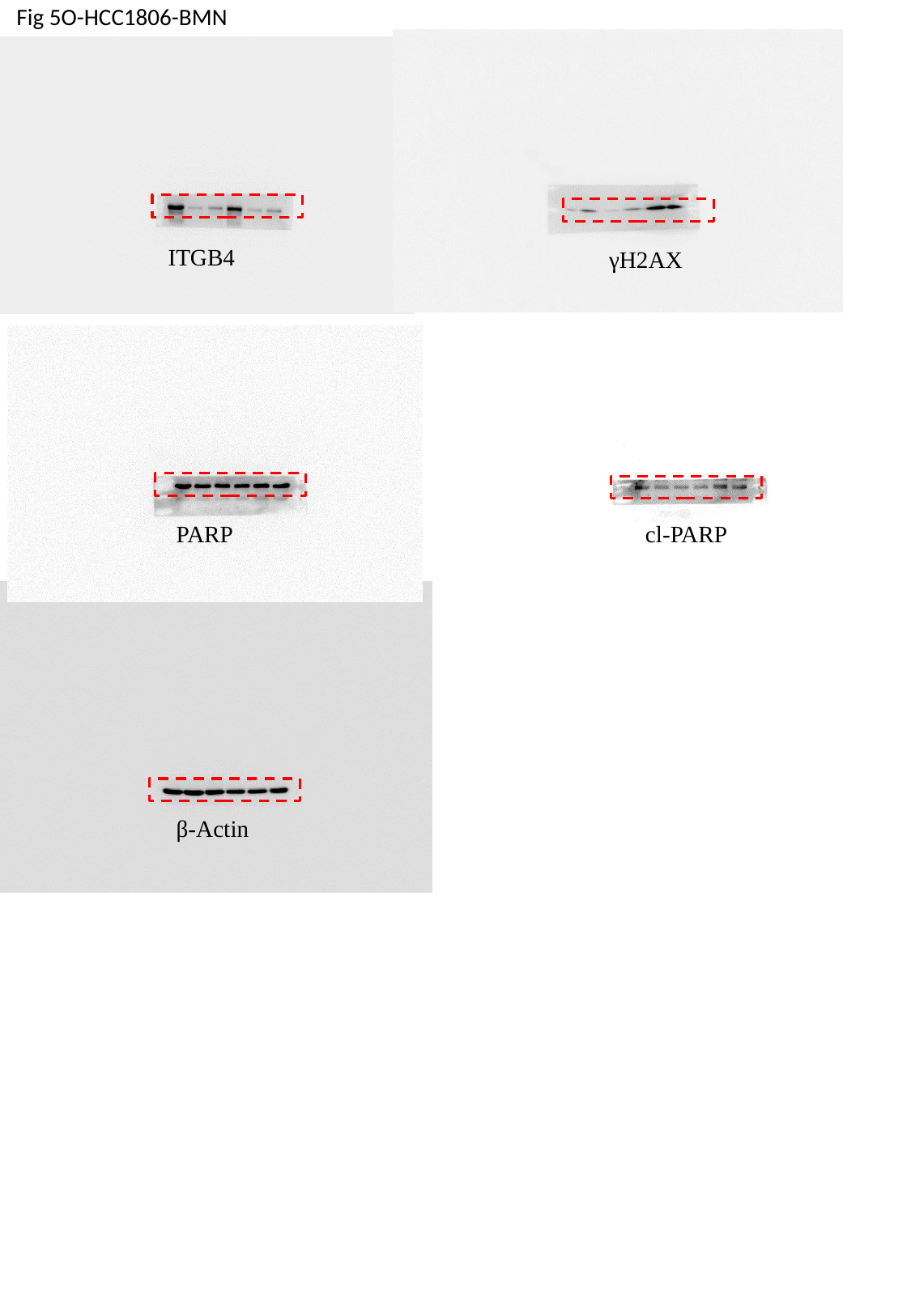

Fig 5O-HCC1806-BMN
ITGB4
γH2AX
PARP
cl-PARP
β-Actin

## Slide 4
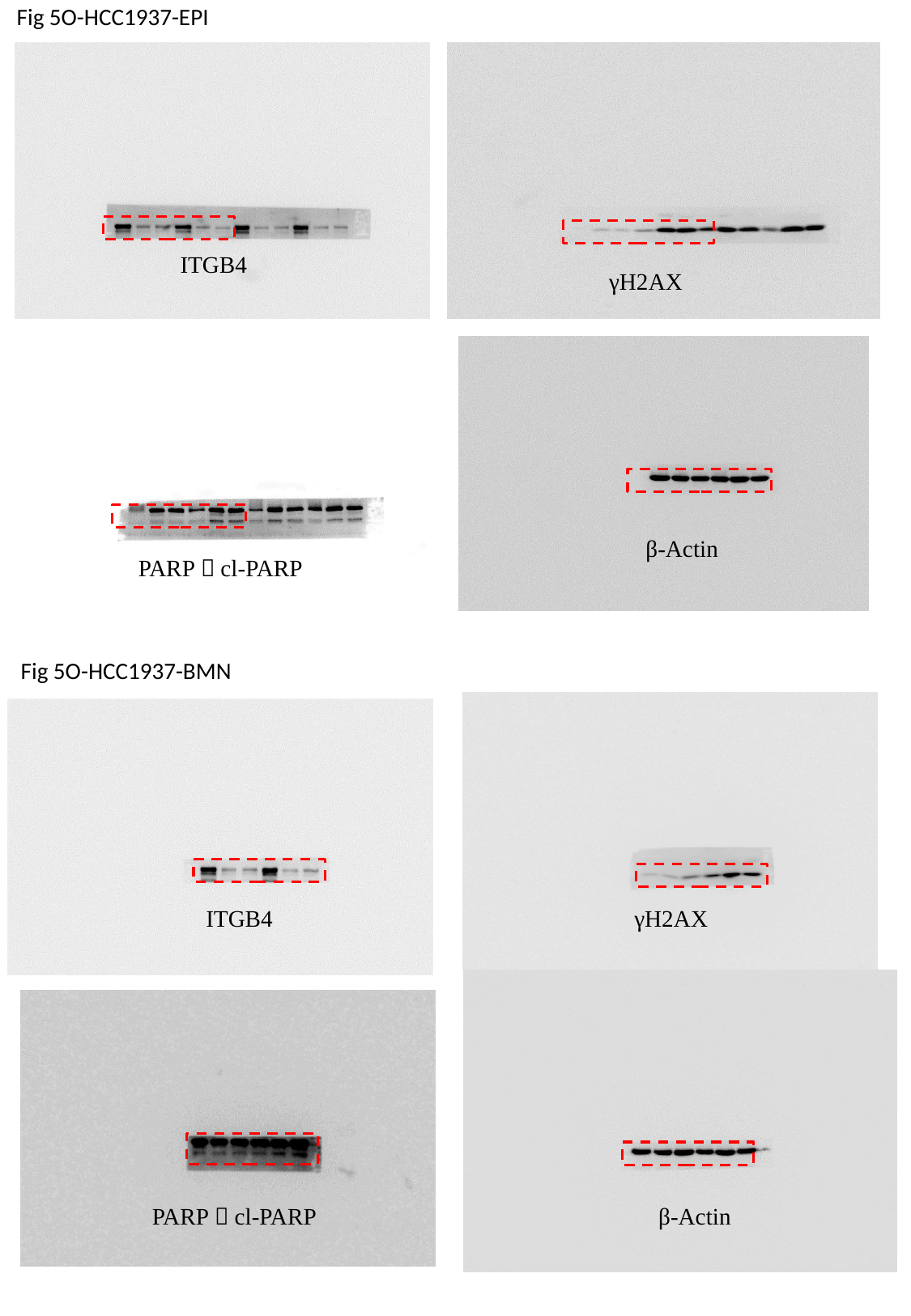

Fig 5O-HCC1937-EPI
ITGB4
γH2AX
β-Actin
PARP，cl-PARP
Fig 5O-HCC1937-BMN
ITGB4
γH2AX
PARP，cl-PARP
β-Actin
