# Supplementary material for: Integrin β4 promotes DNA damage-related drug resistance in triple-negative breast cancer via TNFAIP2/IQGAP1/RAC1
Source: eLife. 2023 Oct 3;12:RP88483. doi: 10.7554/eLife.88483 (PMC10547475; doi:10.7554/eLife.88483)
Supplement: Figure 5—figure supplement 1—source data 1. [file elife-88483-fig5-figsupp1-data1.pptx]

## Slide 1
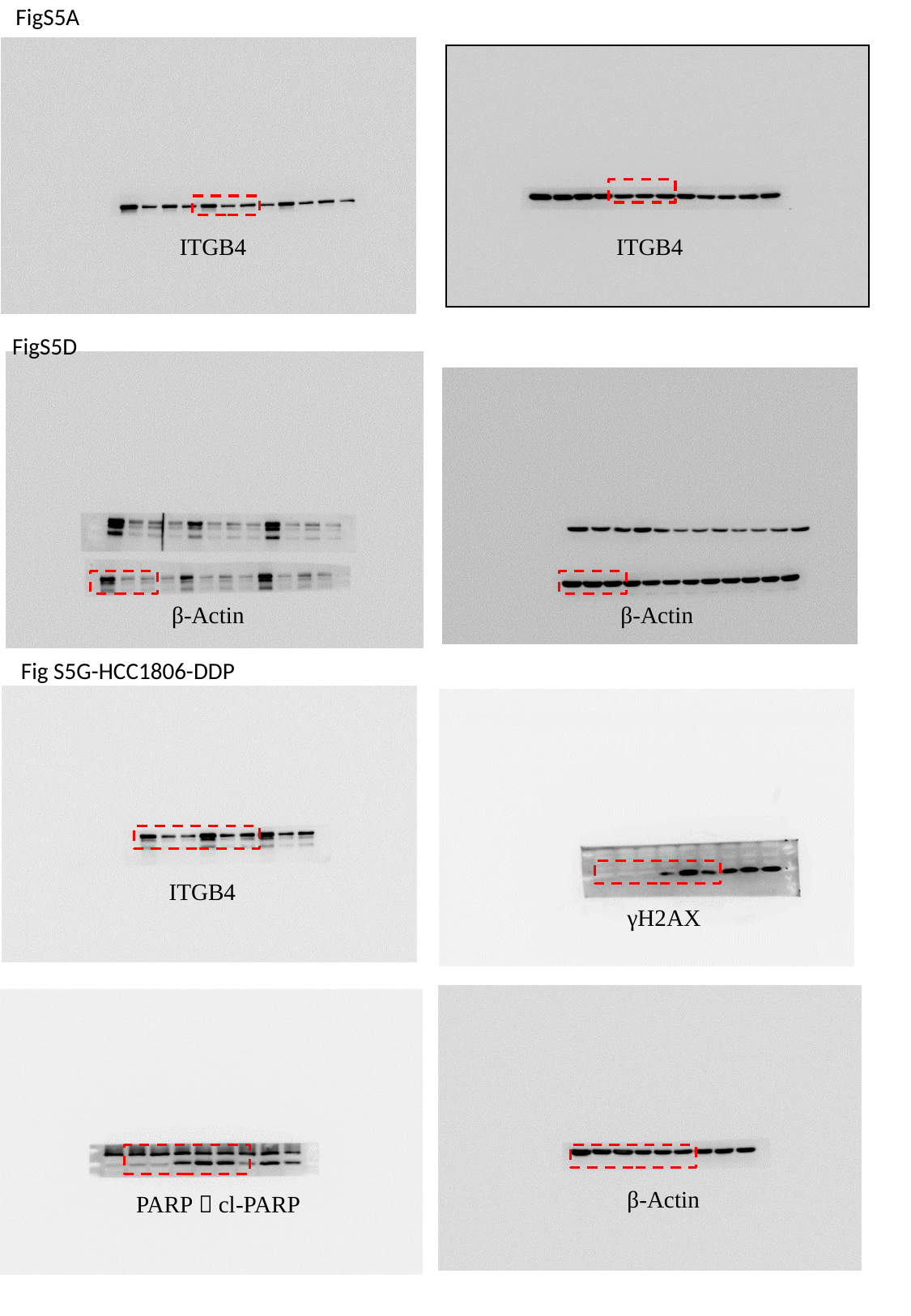

FigS5A
ITGB4
ITGB4
FigS5D
β-Actin
β-Actin
Fig S5G-HCC1806-DDP
ITGB4
γH2AX
β-Actin
PARP，cl-PARP

## Slide 2
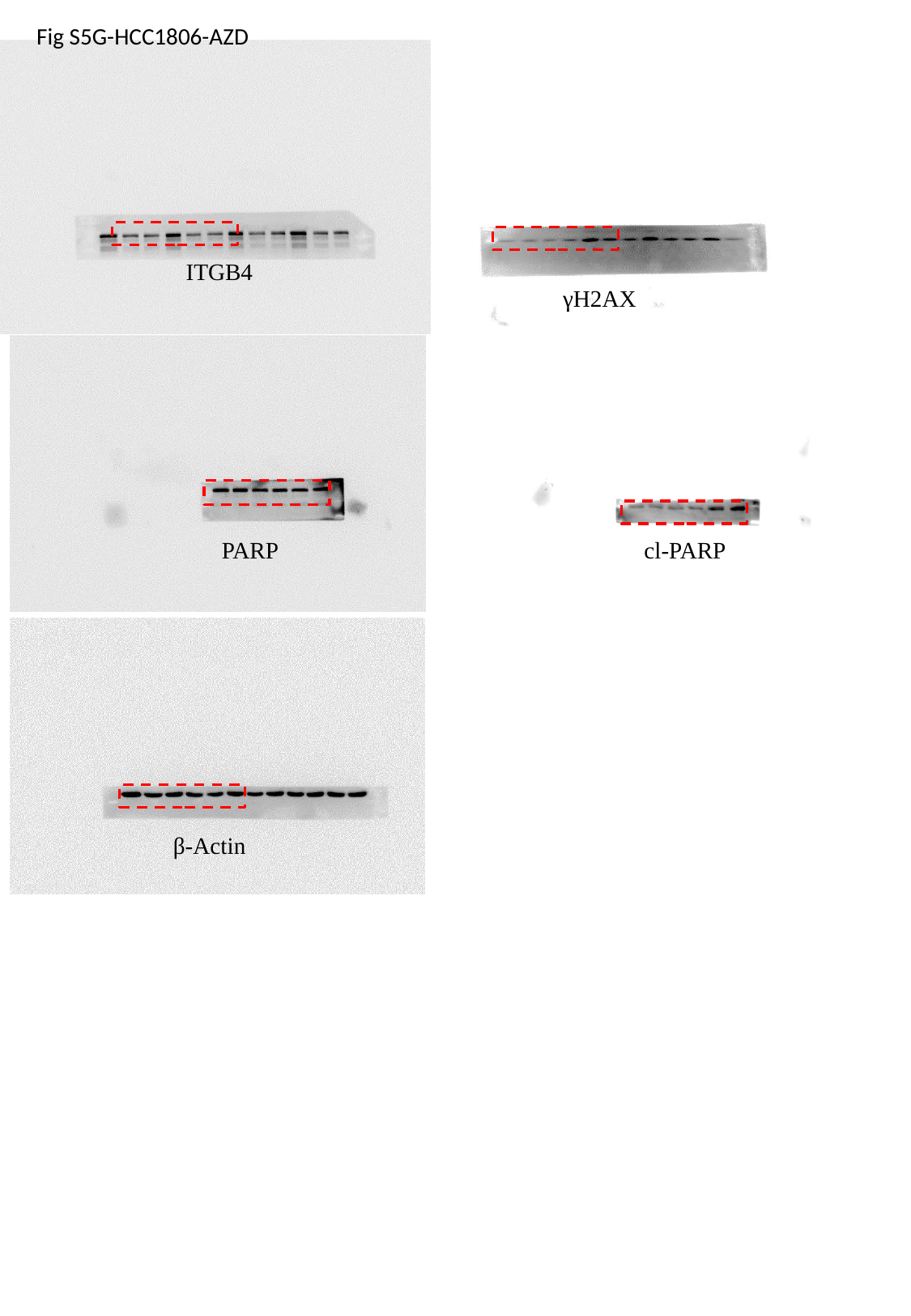

Fig S5G-HCC1806-AZD
ITGB4
γH2AX
PARP
cl-PARP
β-Actin

## Slide 3
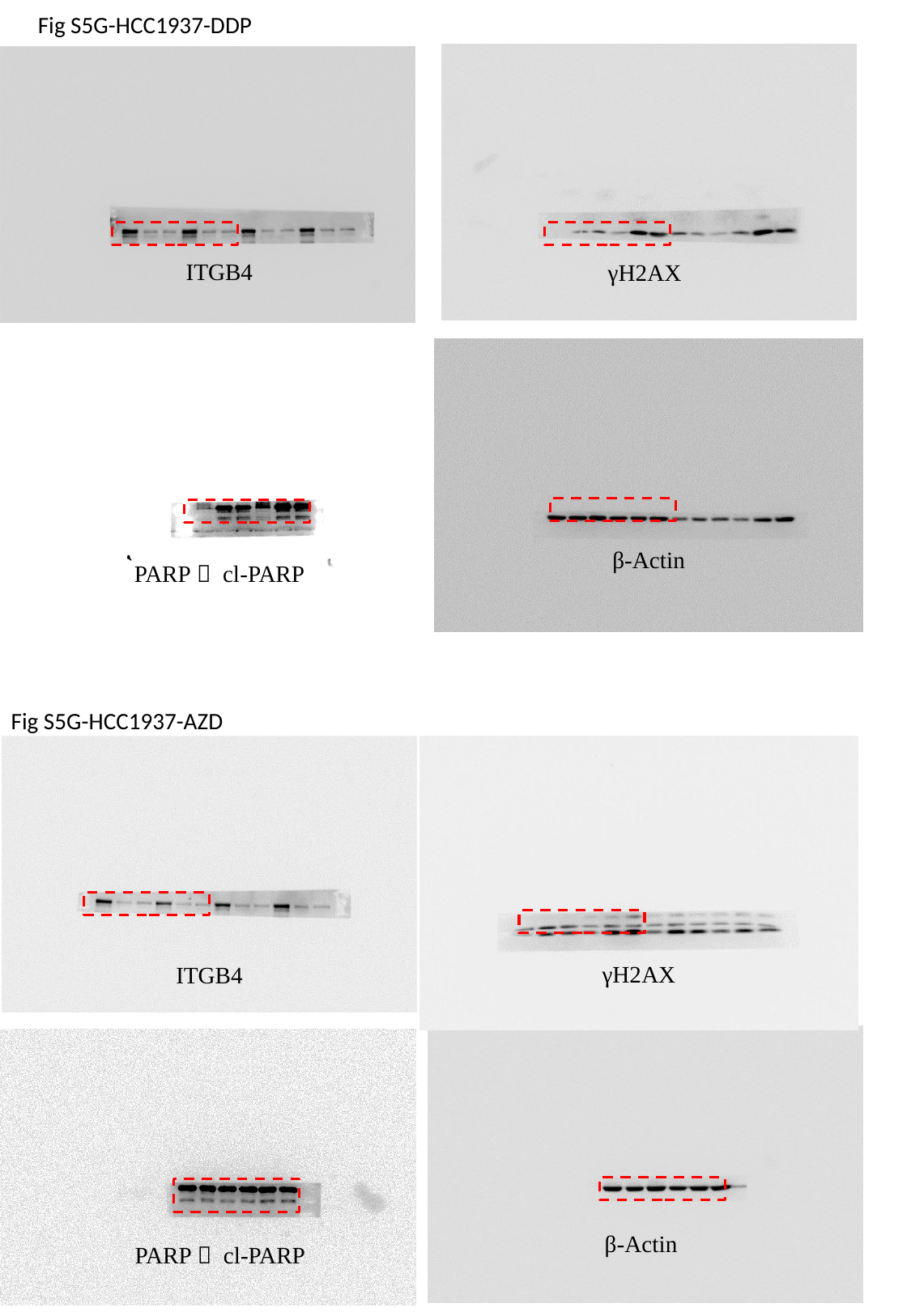

Fig S5G-HCC1937-DDP
ITGB4
γH2AX
β-Actin
PARP， cl-PARP
Fig S5G-HCC1937-AZD
γH2AX
ITGB4
β-Actin
PARP， cl-PARP
