# Supplementary material for: Integrin β4 promotes DNA damage-related drug resistance in triple-negative breast cancer via TNFAIP2/IQGAP1/RAC1
Source: eLife. 2023 Oct 3;12:RP88483. doi: 10.7554/eLife.88483 (PMC10547475; doi:10.7554/eLife.88483)
Supplement: Figure 5—figure supplement 2—source data 1. [file elife-88483-fig5-figsupp2-data1.pptx]

## Slide 1
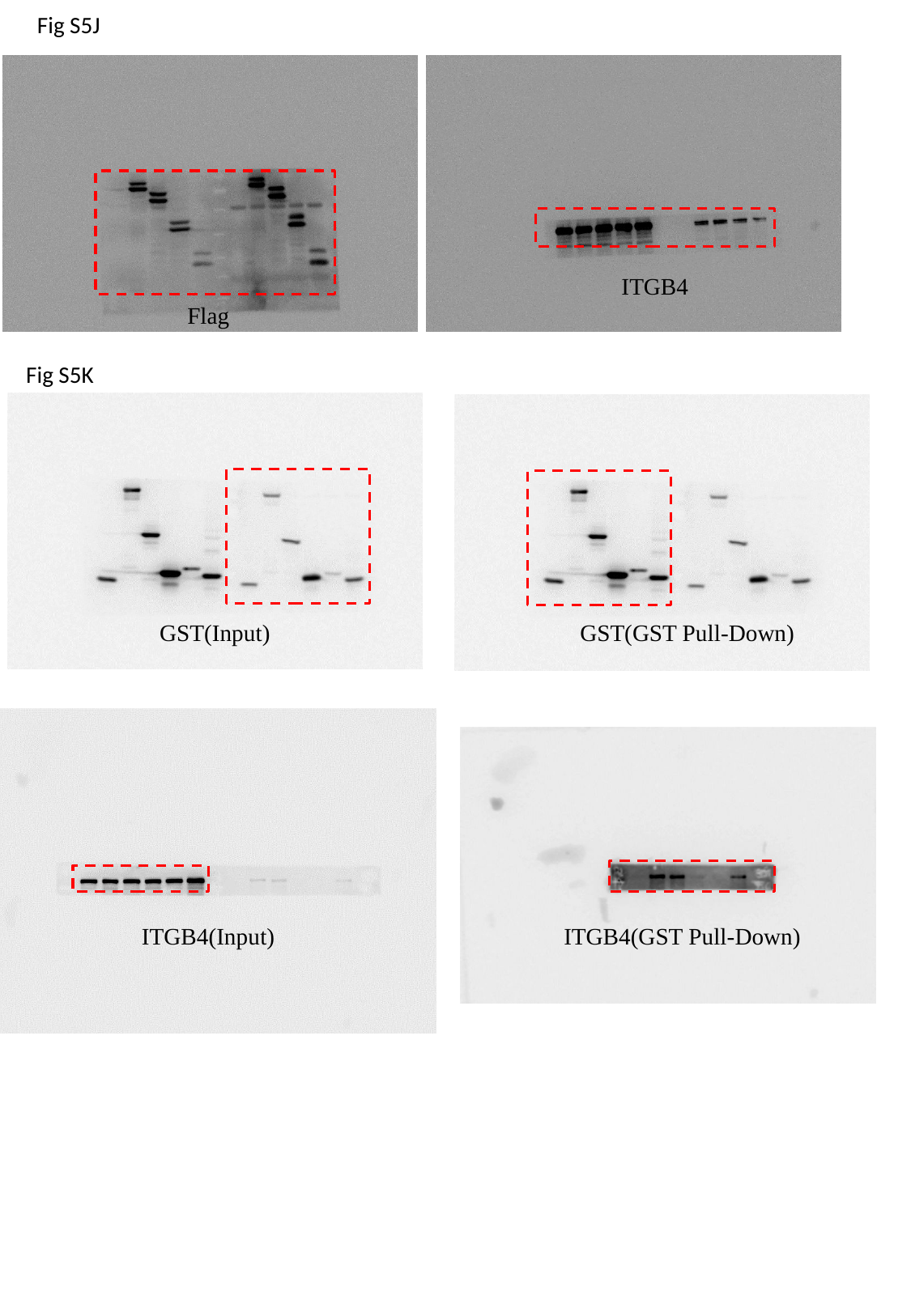

Fig S5J
ITGB4
Flag
Fig S5K
GST(Input)
GST(GST Pull-Down)
ITGB4(GST Pull-Down)
ITGB4(Input)

## Slide 2
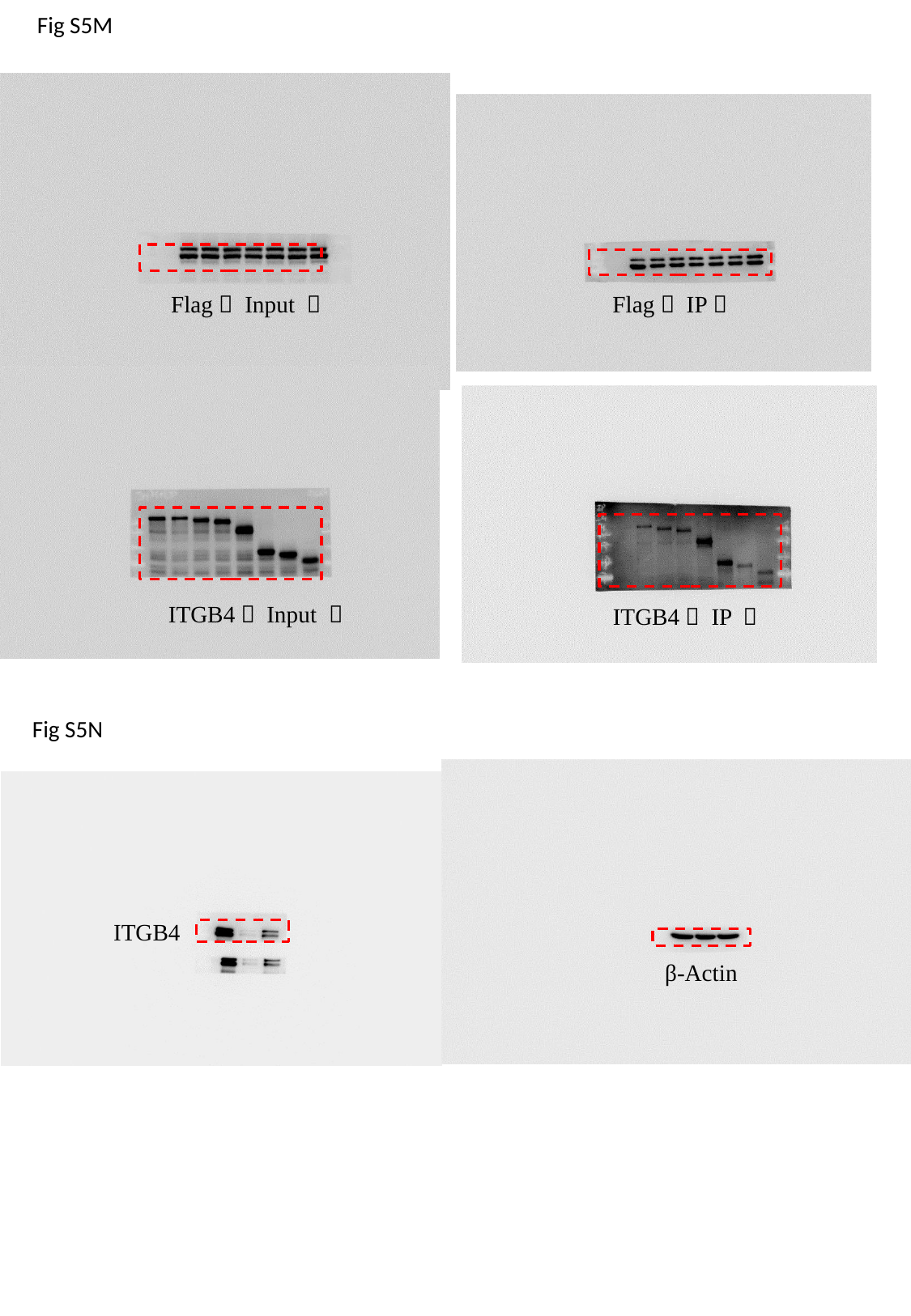

Fig S5M
Flag（ Input ）
Flag（ IP）
ITGB4（ Input ）
ITGB4（ IP ）
Fig S5N
ITGB4
β-Actin
