# Supplementary material for: Integrin β4 promotes DNA damage-related drug resistance in triple-negative breast cancer via TNFAIP2/IQGAP1/RAC1
Source: eLife. 2023 Oct 3;12:RP88483. doi: 10.7554/eLife.88483 (PMC10547475; doi:10.7554/eLife.88483)
Supplement: Figure 6—source data 1. [file elife-88483-fig6-data1.pptx]

## Slide 1
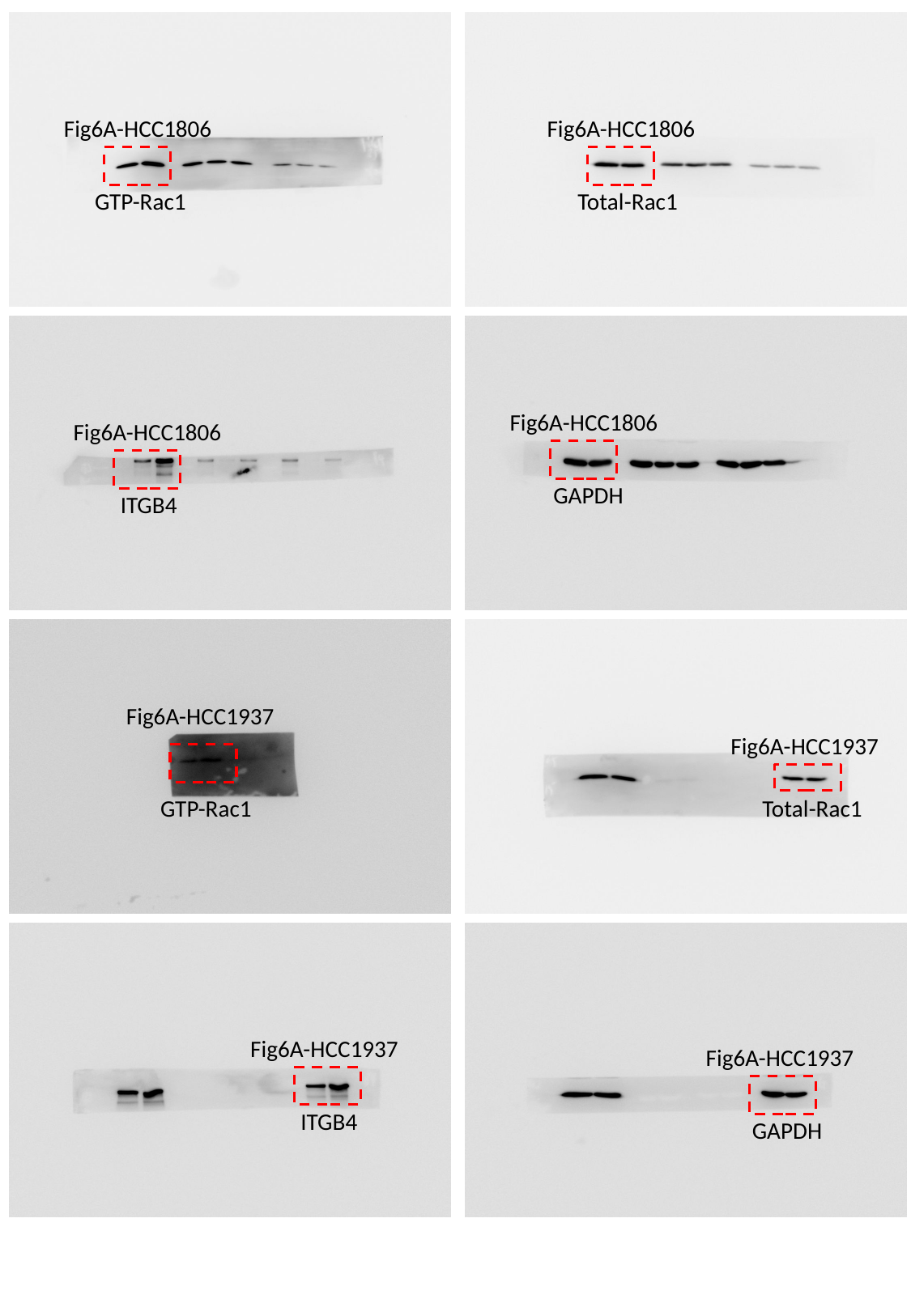

Fig6A-HCC1806
Fig6A-HCC1806
GTP-Rac1
Total-Rac1
Fig6A-HCC1806
Fig6A-HCC1806
GAPDH
ITGB4
Fig6A-HCC1937
Fig6A-HCC1937
GTP-Rac1
Total-Rac1
Fig6A-HCC1937
Fig6A-HCC1937
ITGB4
GAPDH

## Slide 2
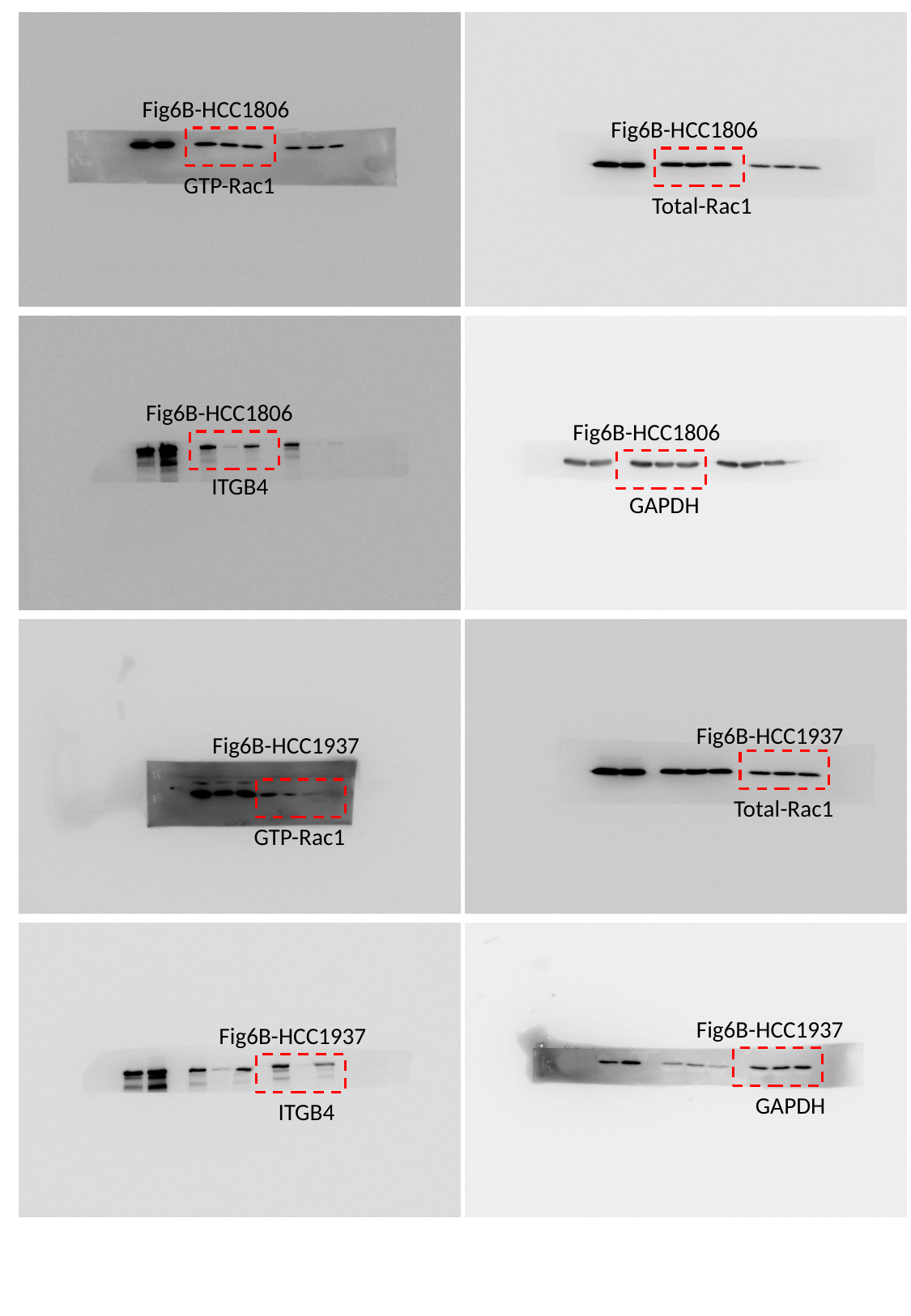

Fig6B-HCC1806
Fig6B-HCC1806
GTP-Rac1
Total-Rac1
Fig6B-HCC1806
Fig6B-HCC1806
ITGB4
GAPDH
Fig6B-HCC1937
Fig6B-HCC1937
Total-Rac1
GTP-Rac1
Fig6B-HCC1937
Fig6B-HCC1937
GAPDH
ITGB4

## Slide 3
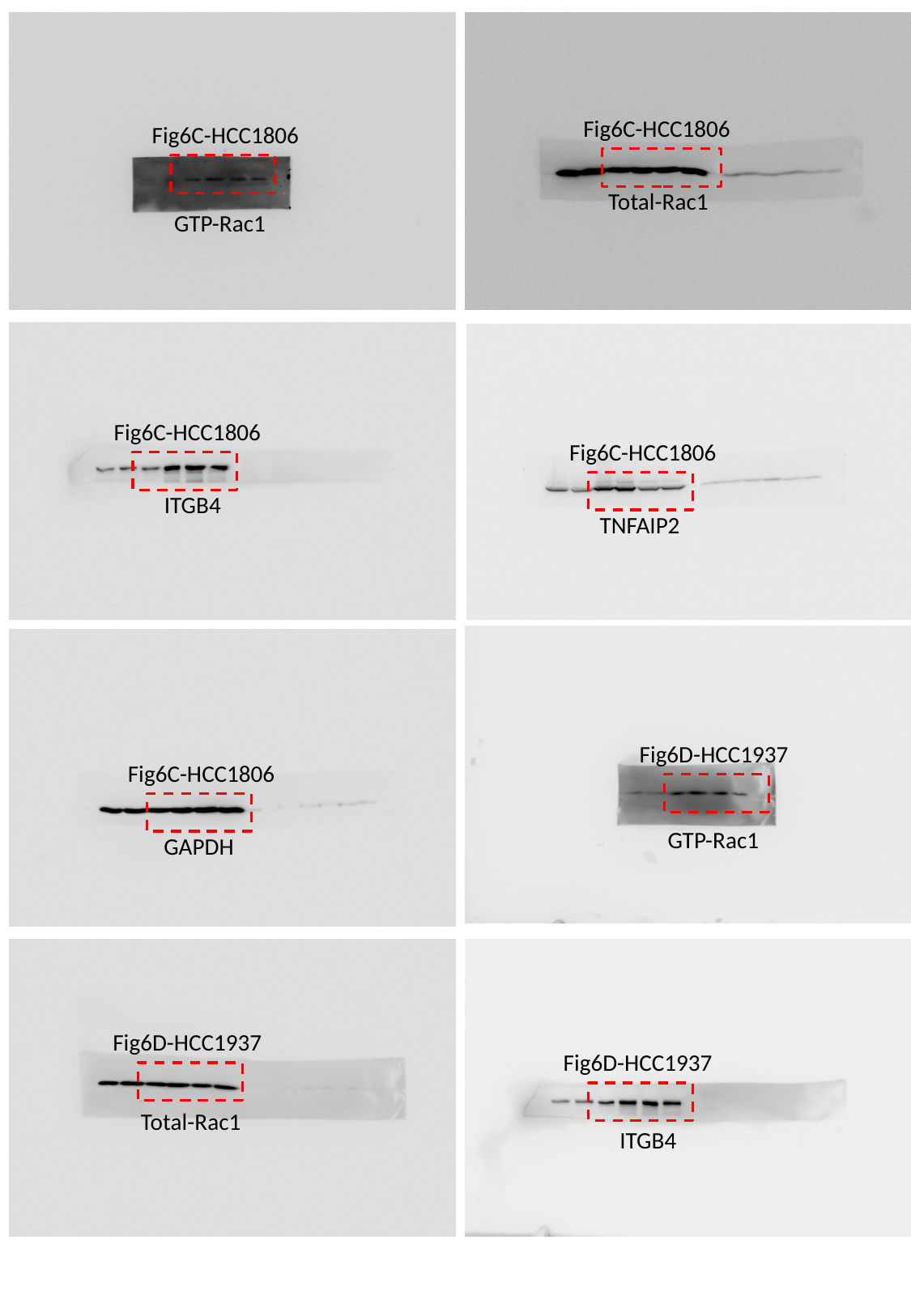

Fig6C-HCC1806
Fig6C-HCC1806
Total-Rac1
GTP-Rac1
Fig6C-HCC1806
Fig6C-HCC1806
ITGB4
TNFAIP2
Fig6D-HCC1937
Fig6C-HCC1806
GTP-Rac1
GAPDH
Fig6D-HCC1937
Fig6D-HCC1937
Total-Rac1
ITGB4

## Slide 4
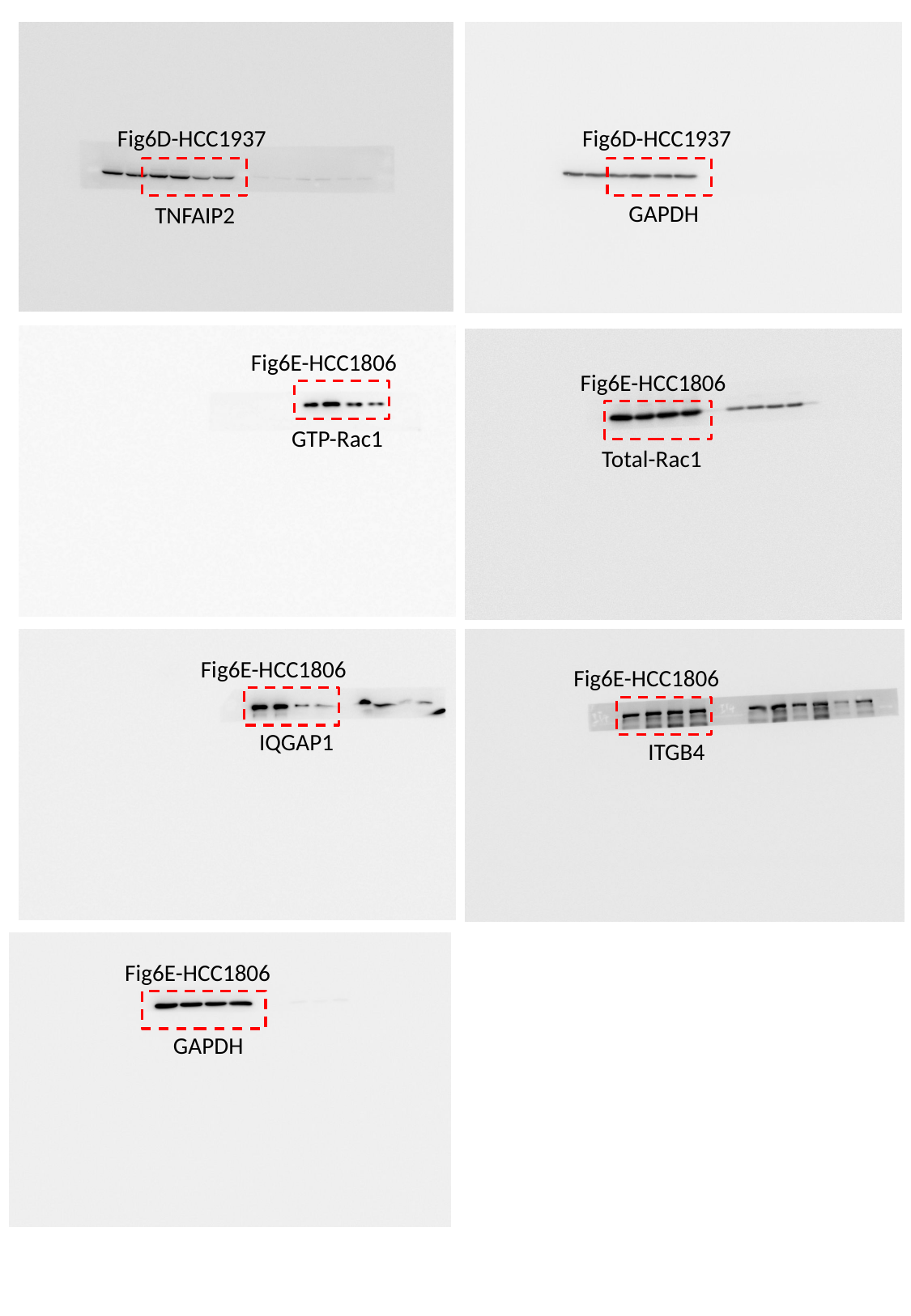

Fig6D-HCC1937
Fig6D-HCC1937
GAPDH
TNFAIP2
Fig6E-HCC1806
Fig6E-HCC1806
GTP-Rac1
Total-Rac1
Fig6E-HCC1806
Fig6E-HCC1806
IQGAP1
ITGB4
Fig6E-HCC1806
GAPDH

## Slide 5
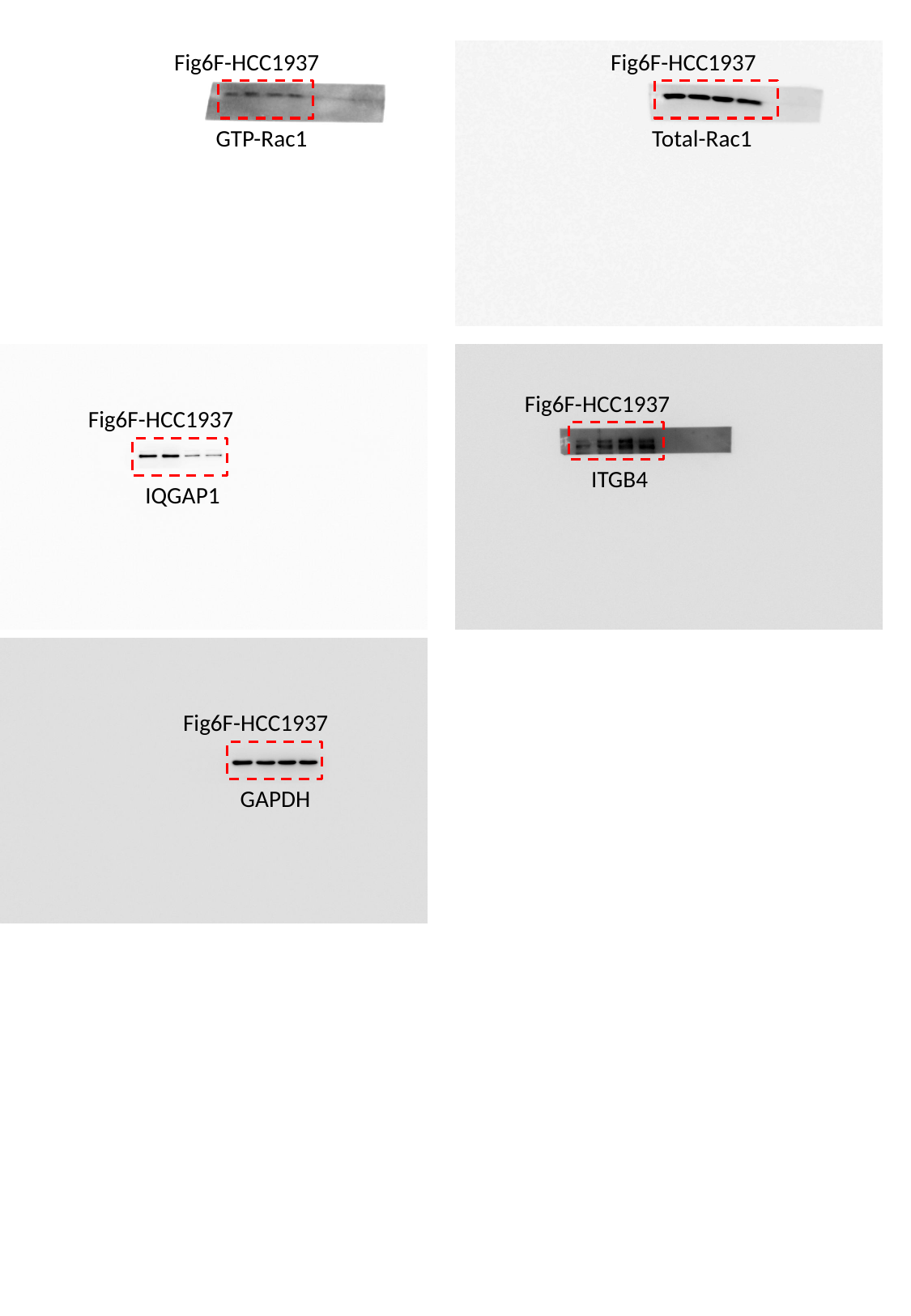

Fig6F-HCC1937
Fig6F-HCC1937
GTP-Rac1
Total-Rac1
Fig6F-HCC1937
Fig6F-HCC1937
ITGB4
IQGAP1
Fig6F-HCC1937
GAPDH
