# Supplementary material for: Integrin β4 promotes DNA damage-related drug resistance in triple-negative breast cancer via TNFAIP2/IQGAP1/RAC1
Source: eLife. 2023 Oct 3;12:RP88483. doi: 10.7554/eLife.88483 (PMC10547475; doi:10.7554/eLife.88483)
Supplement: Figure 7—source data 1. [file elife-88483-fig7-data1.pptx]

## Slide 1
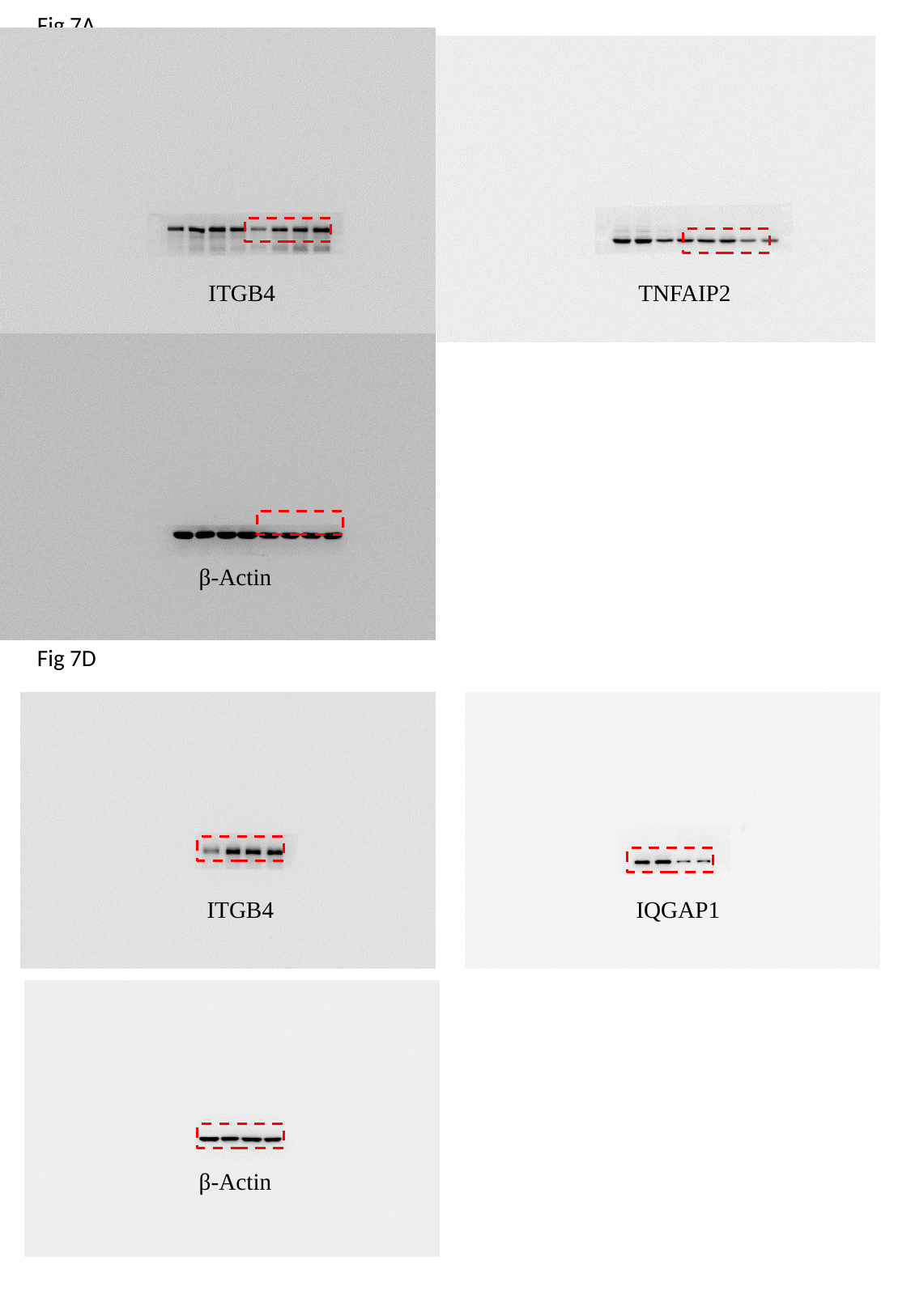

Fig 7A
ITGB4
TNFAIP2
β-Actin
Fig 7D
IQGAP1
ITGB4
β-Actin

## Slide 2
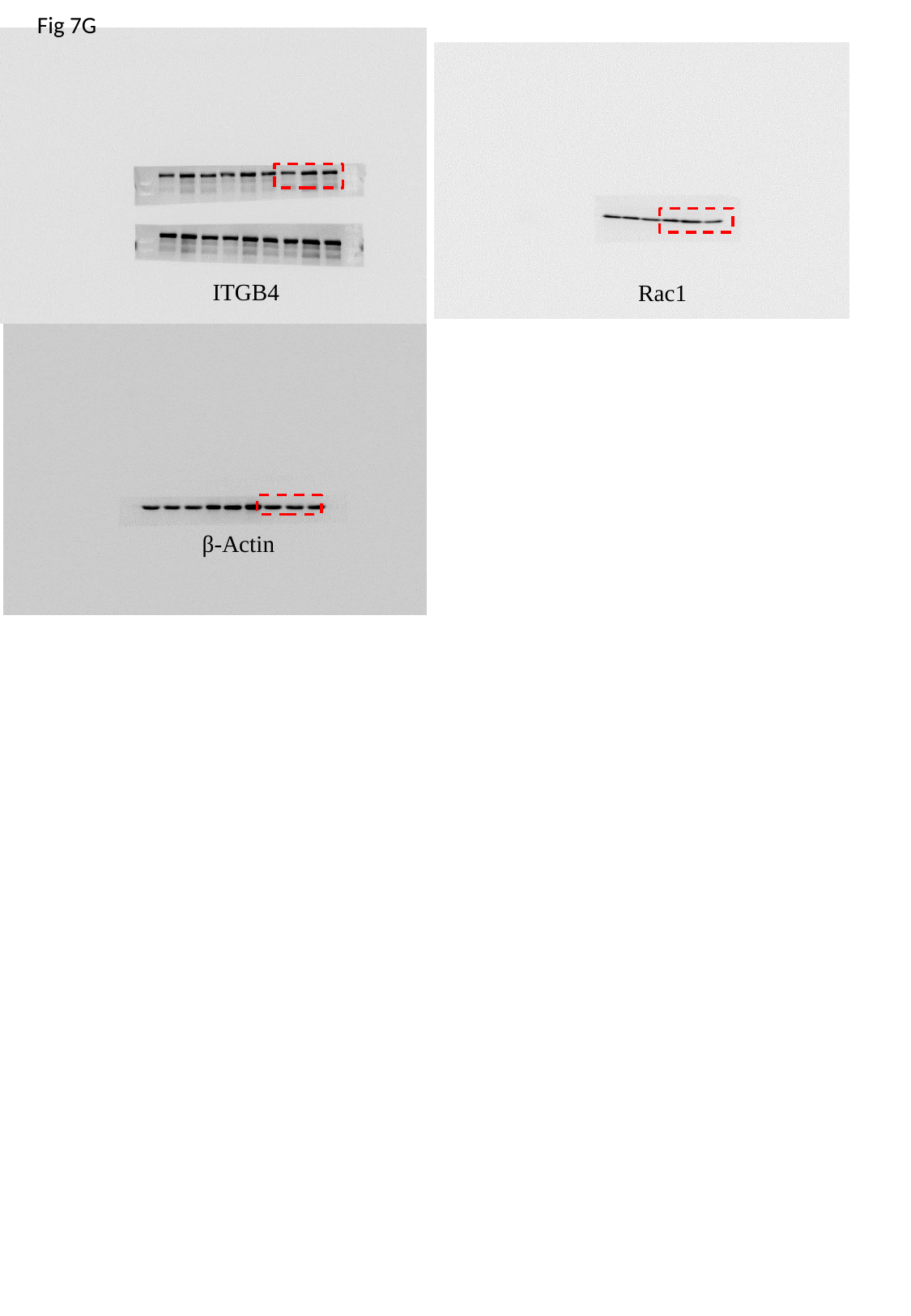

Fig 7G
ITGB4
Rac1
β-Actin

## Slide 3
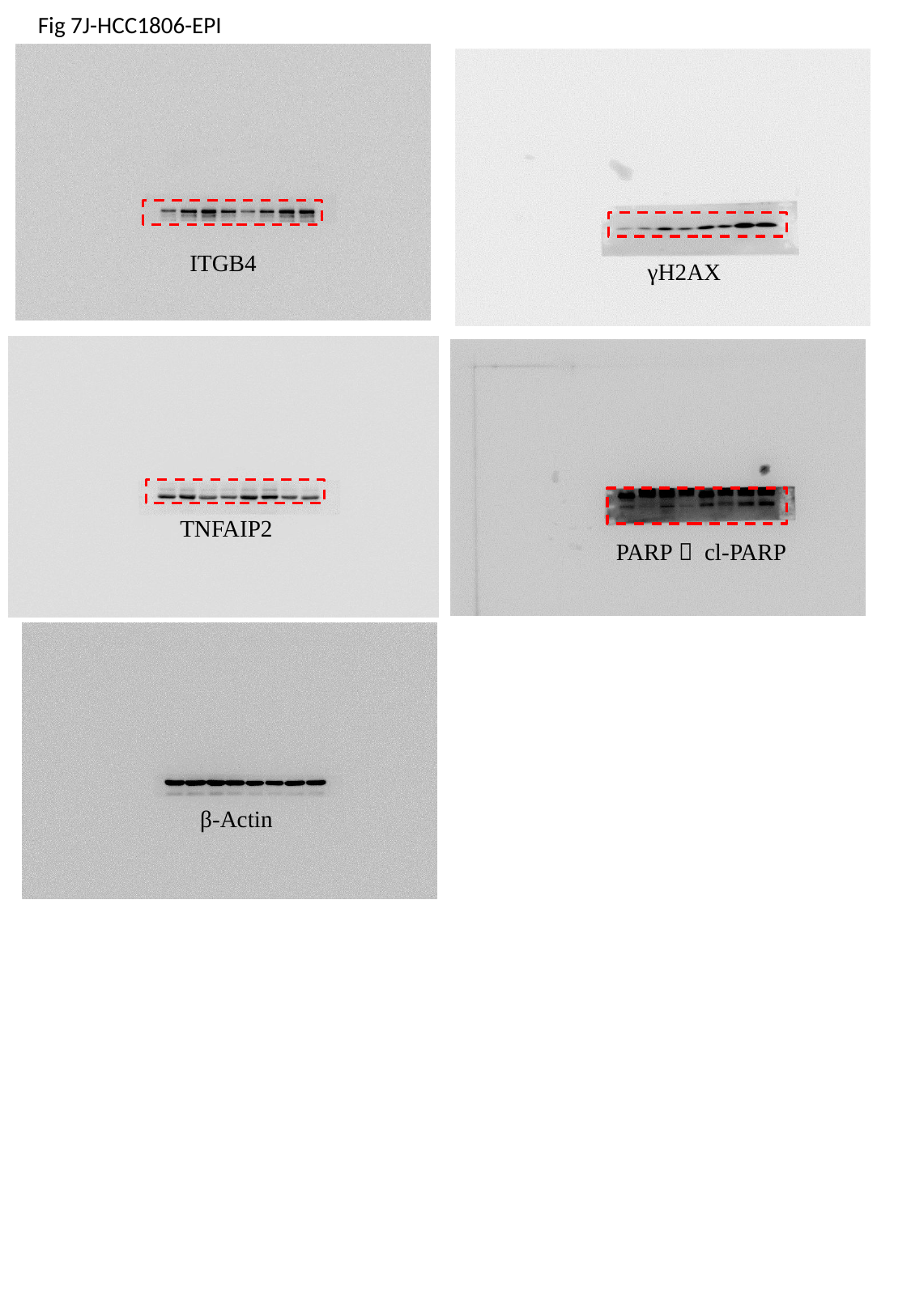

Fig 7J-HCC1806-EPI
ITGB4
γH2AX
TNFAIP2
PARP， cl-PARP
β-Actin

## Slide 4
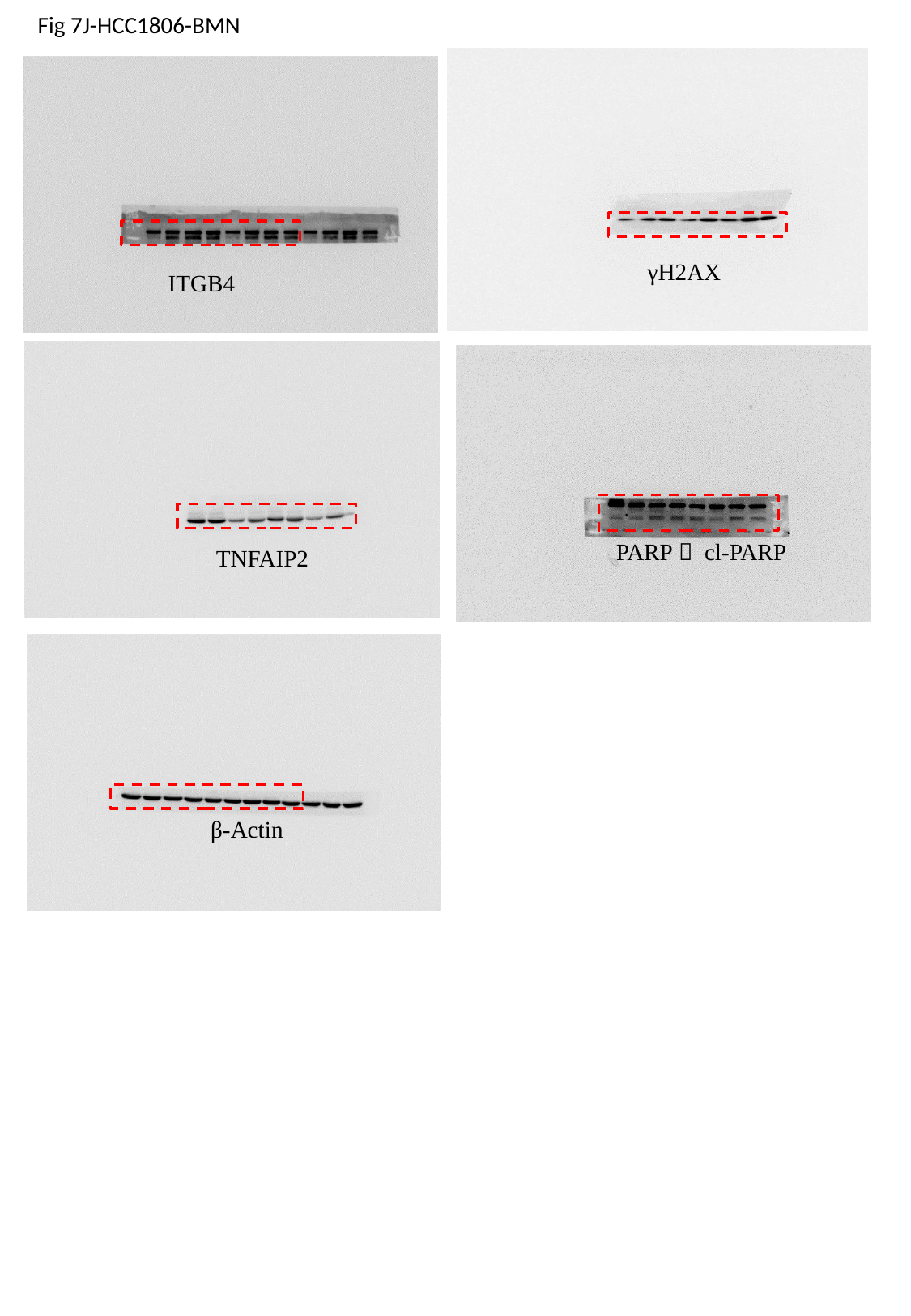

Fig 7J-HCC1806-BMN
γH2AX
ITGB4
PARP， cl-PARP
TNFAIP2
β-Actin

## Slide 5
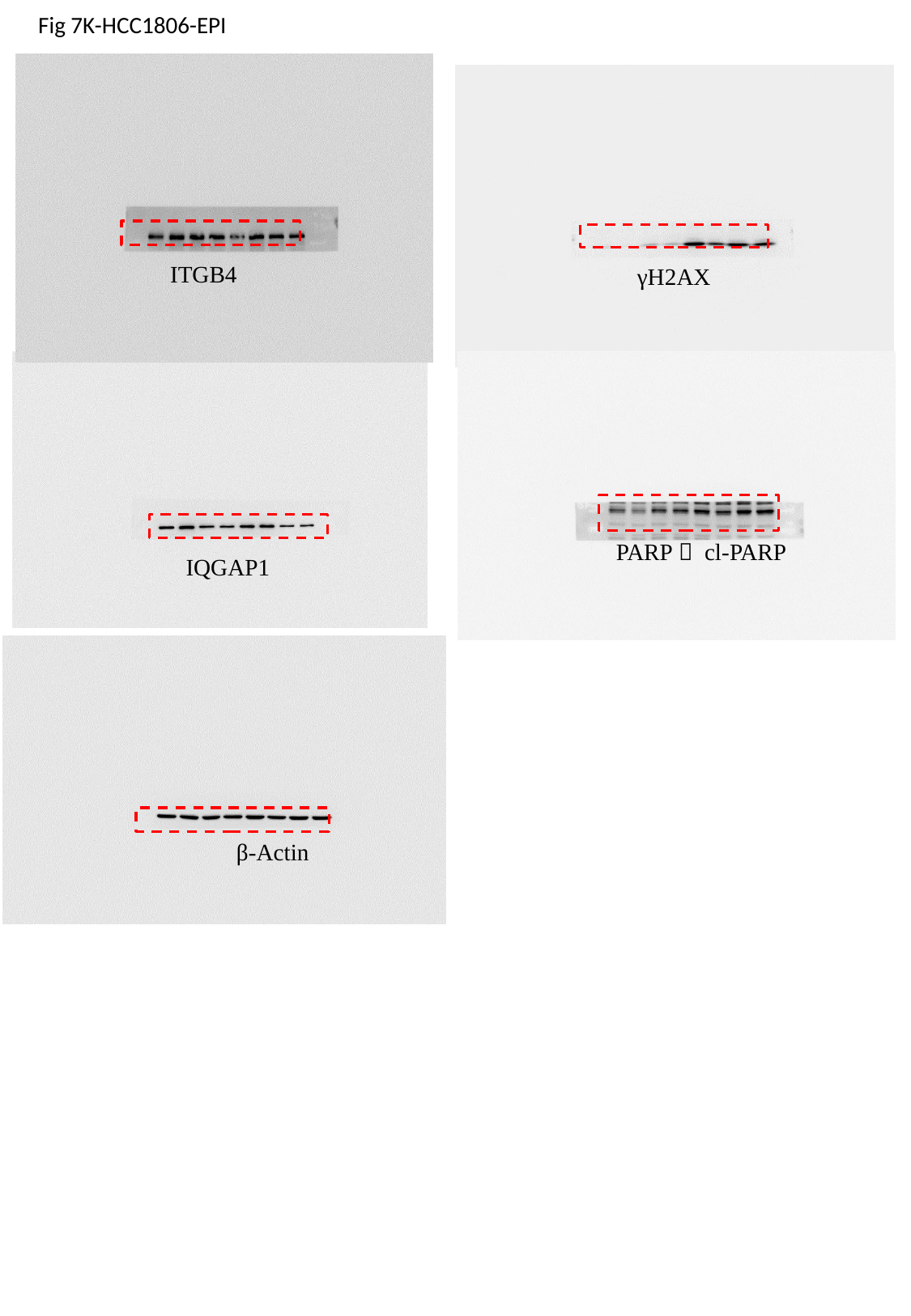

Fig 7K-HCC1806-EPI
ITGB4
γH2AX
PARP， cl-PARP
IQGAP1
β-Actin

## Slide 6
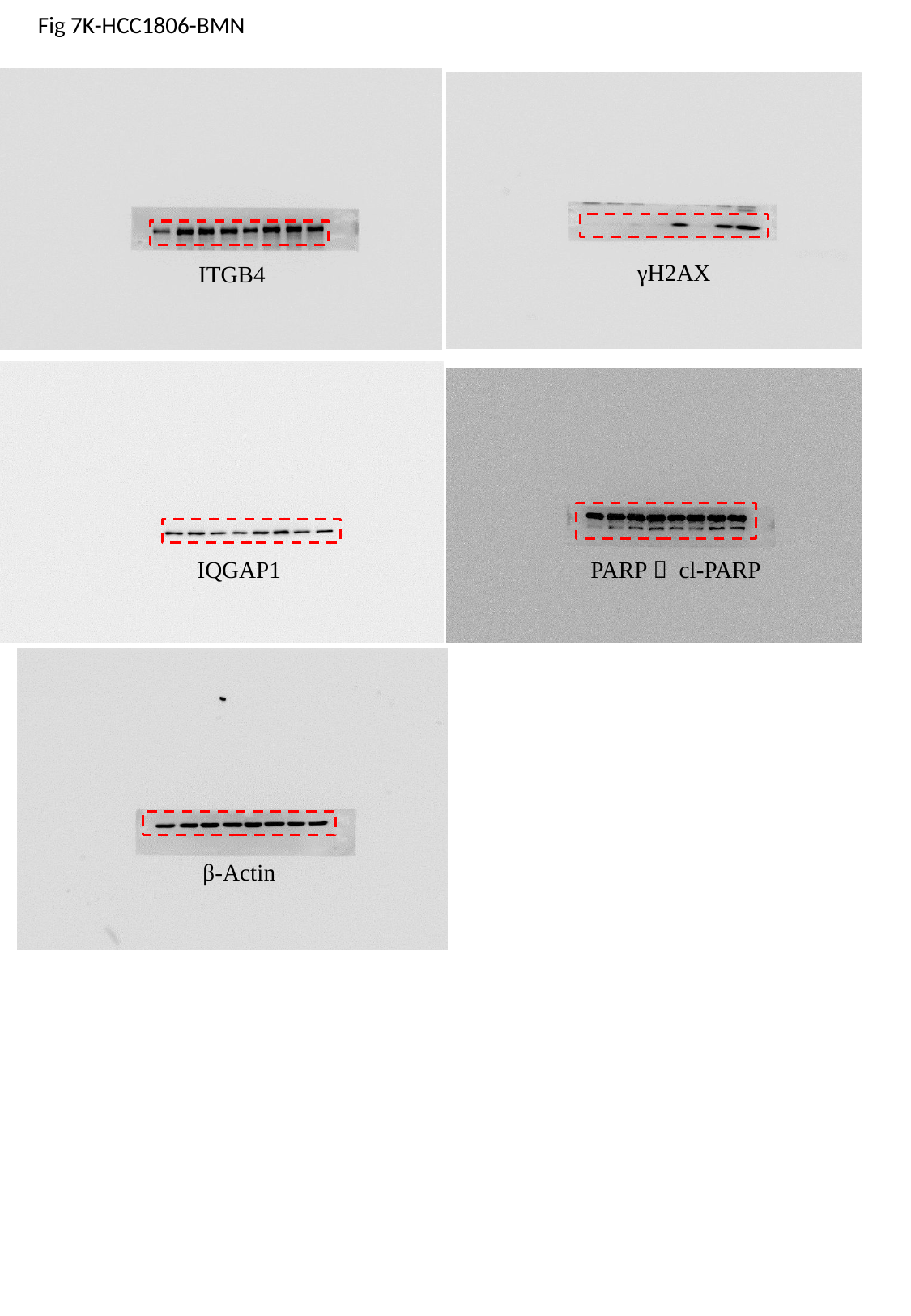

Fig 7K-HCC1806-BMN
γH2AX
ITGB4
IQGAP1
PARP， cl-PARP
β-Actin

## Slide 7
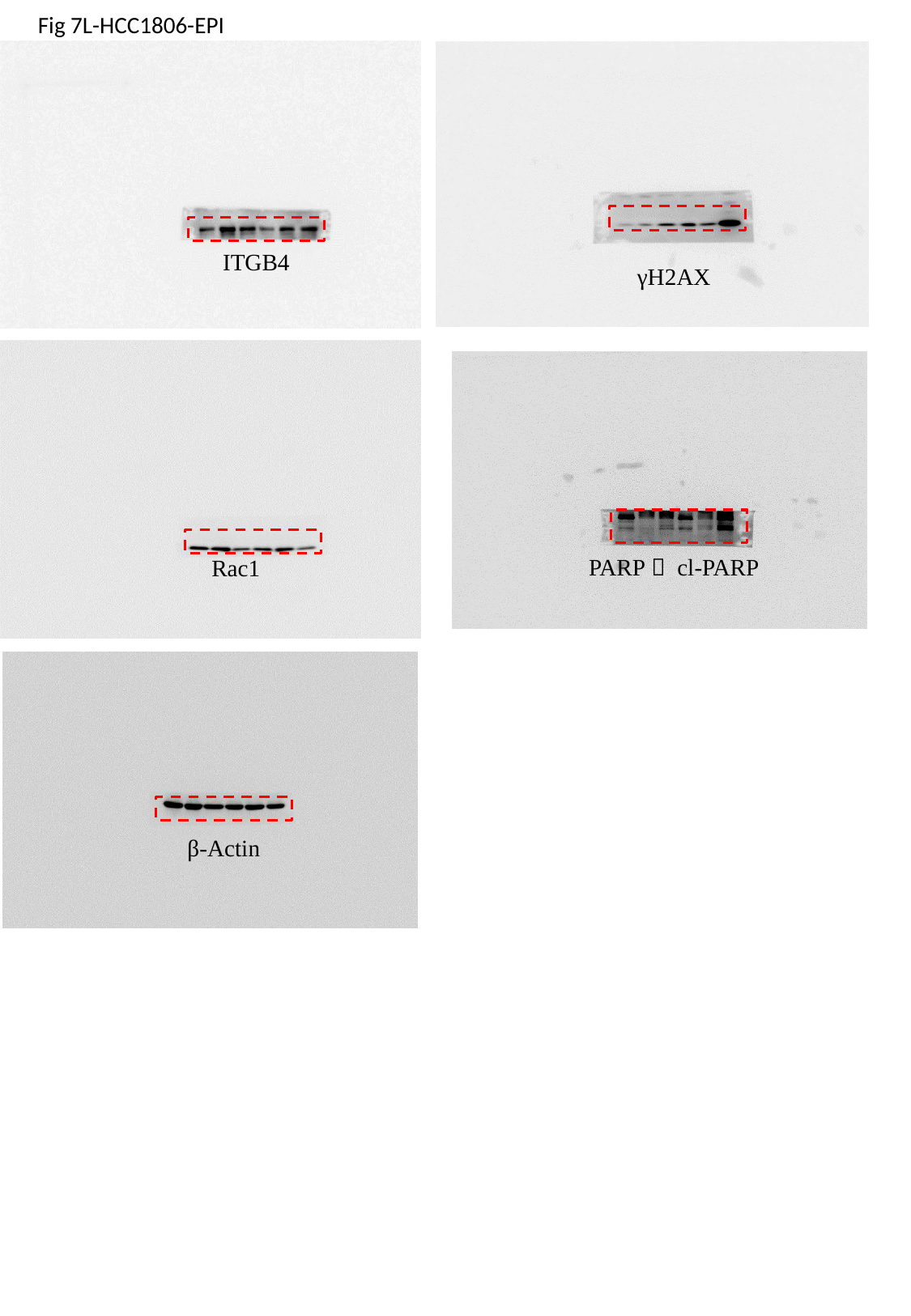

Fig 7L-HCC1806-EPI
ITGB4
γH2AX
PARP， cl-PARP
Rac1
β-Actin

## Slide 8
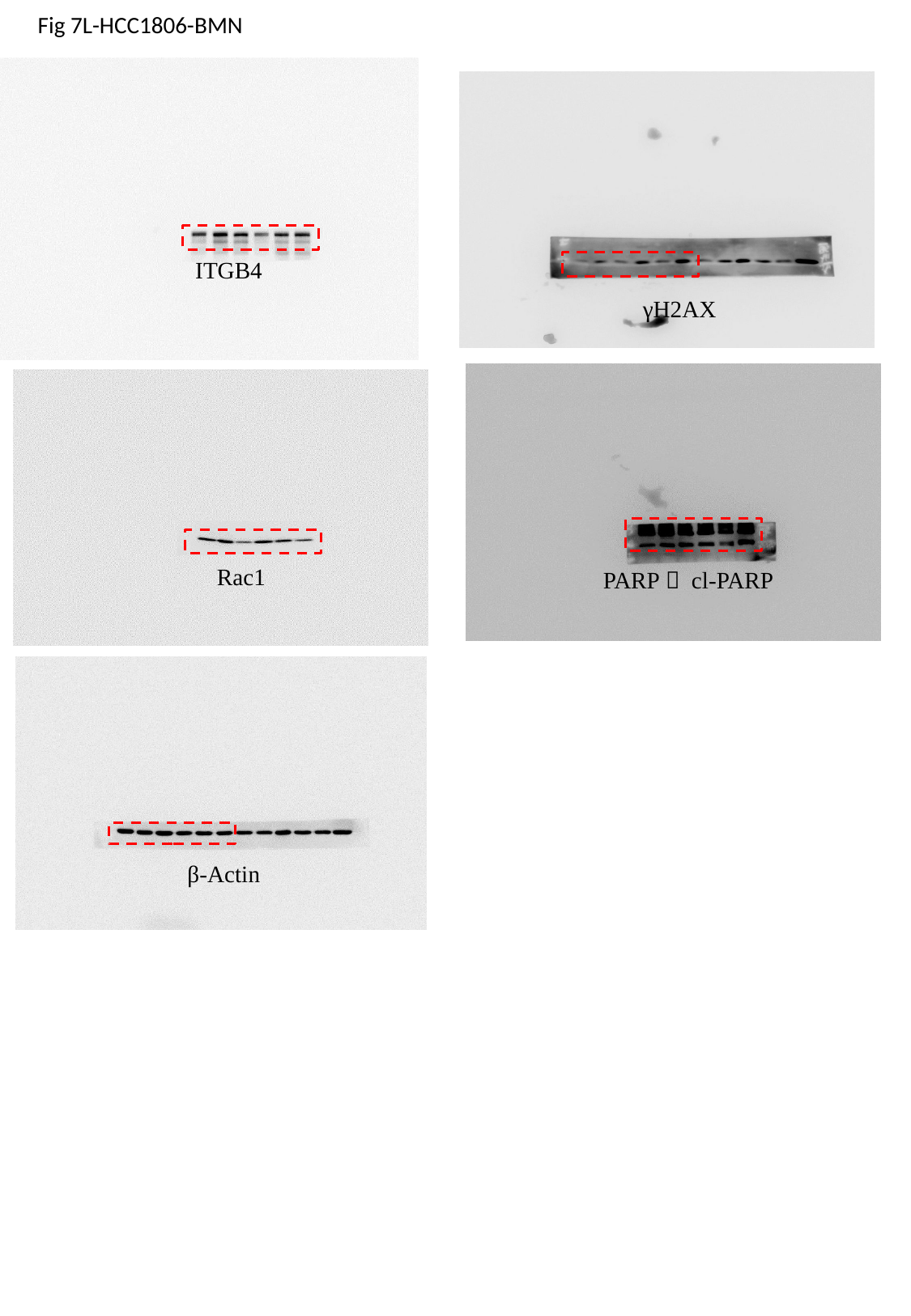

Fig 7L-HCC1806-BMN
ITGB4
γH2AX
Rac1
PARP， cl-PARP
β-Actin
