# Supplementary material for: Integrin β4 promotes DNA damage-related drug resistance in triple-negative breast cancer via TNFAIP2/IQGAP1/RAC1
Source: eLife. 2023 Oct 3;12:RP88483. doi: 10.7554/eLife.88483 (PMC10547475; doi:10.7554/eLife.88483)
Supplement: Figure 7—figure supplement 1—source data 1. [file elife-88483-fig7-figsupp1-data1.pptx]

## Slide 1
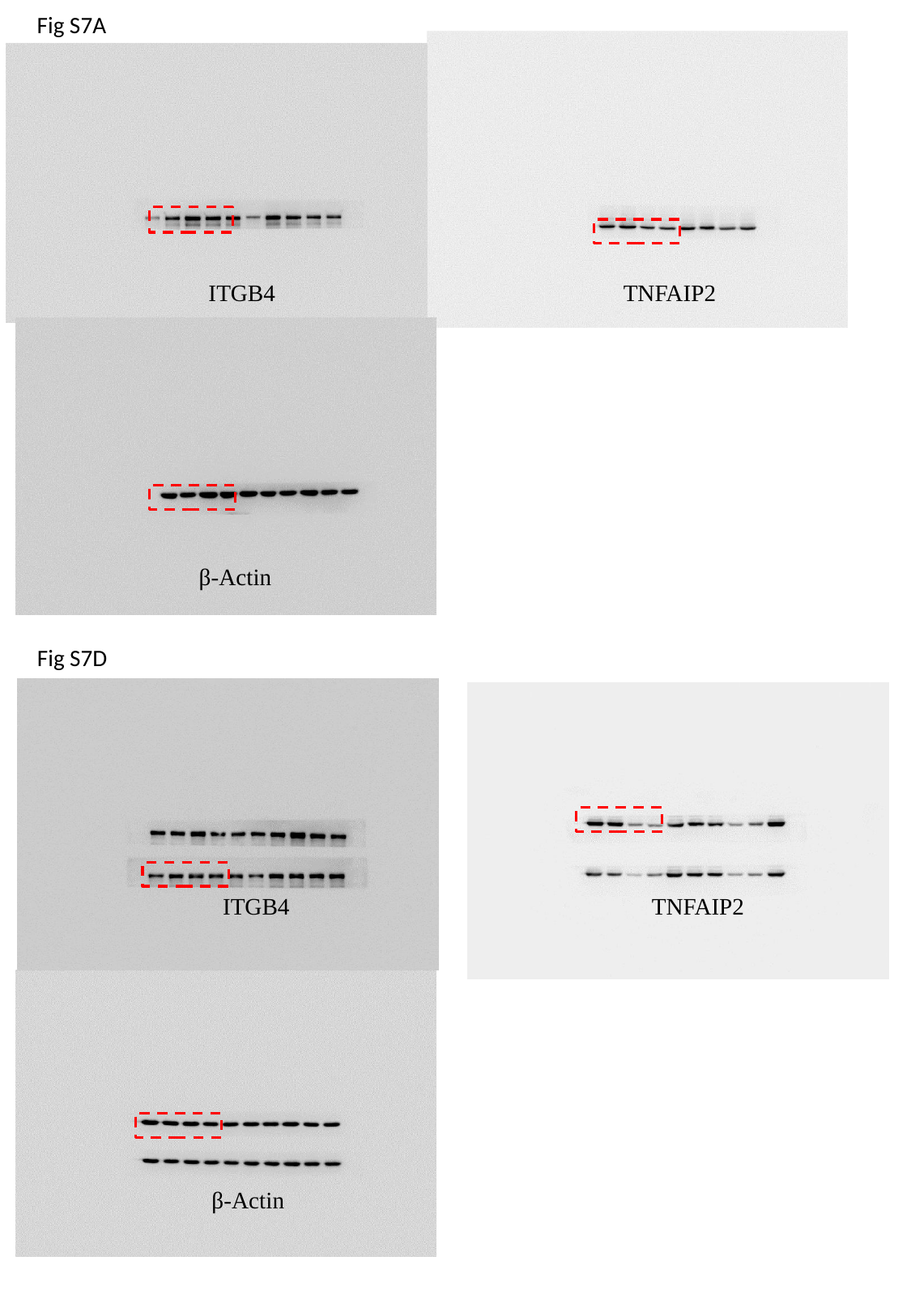

Fig S7A
TNFAIP2
ITGB4
β-Actin
Fig S7D
TNFAIP2
ITGB4
β-Actin

## Slide 2
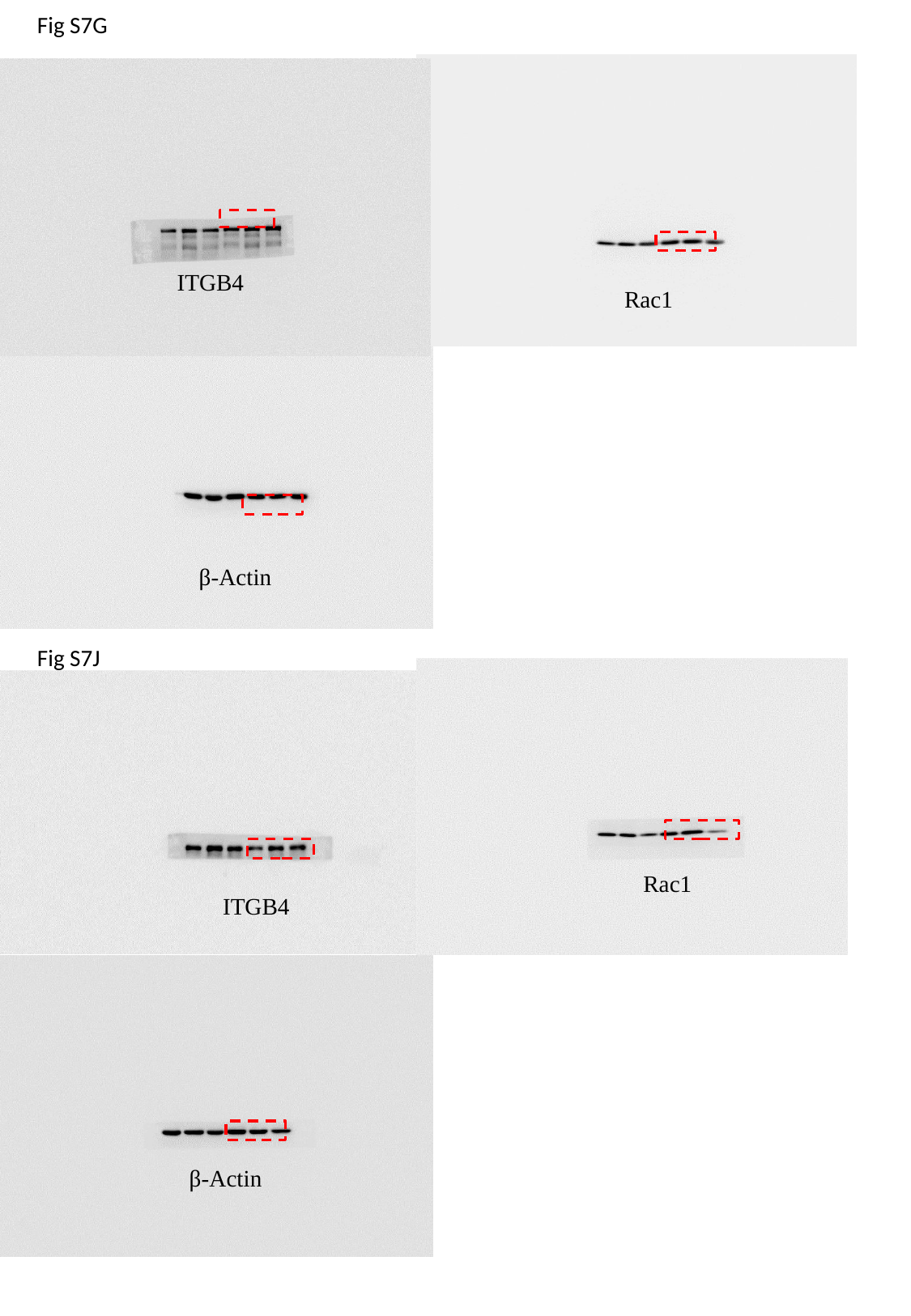

Fig S7G
ITGB4
Rac1
β-Actin
Fig S7J
Rac1
ITGB4
β-Actin

## Slide 3
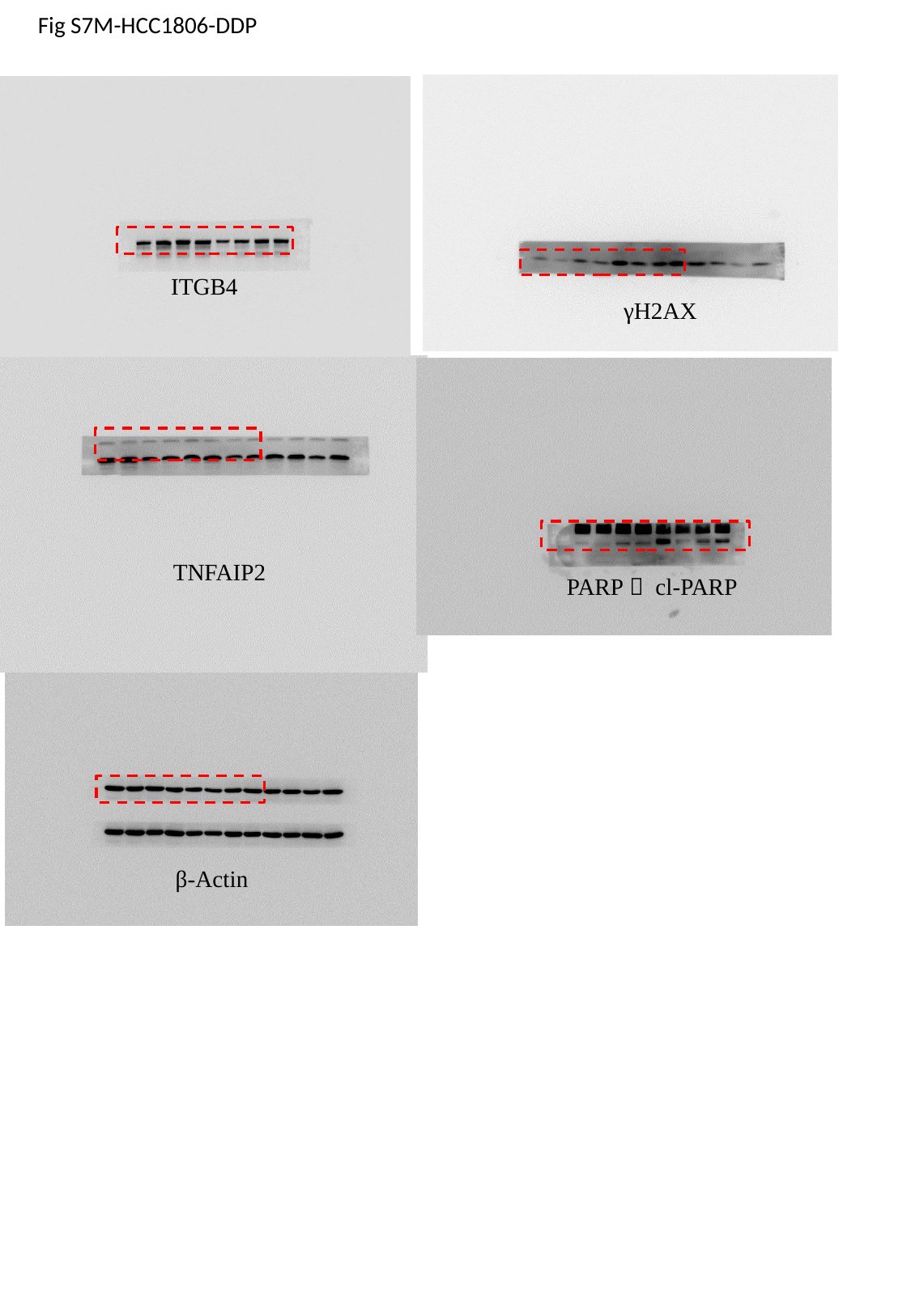

Fig S7M-HCC1806-DDP
ITGB4
γH2AX
TNFAIP2
PARP， cl-PARP
β-Actin

## Slide 4
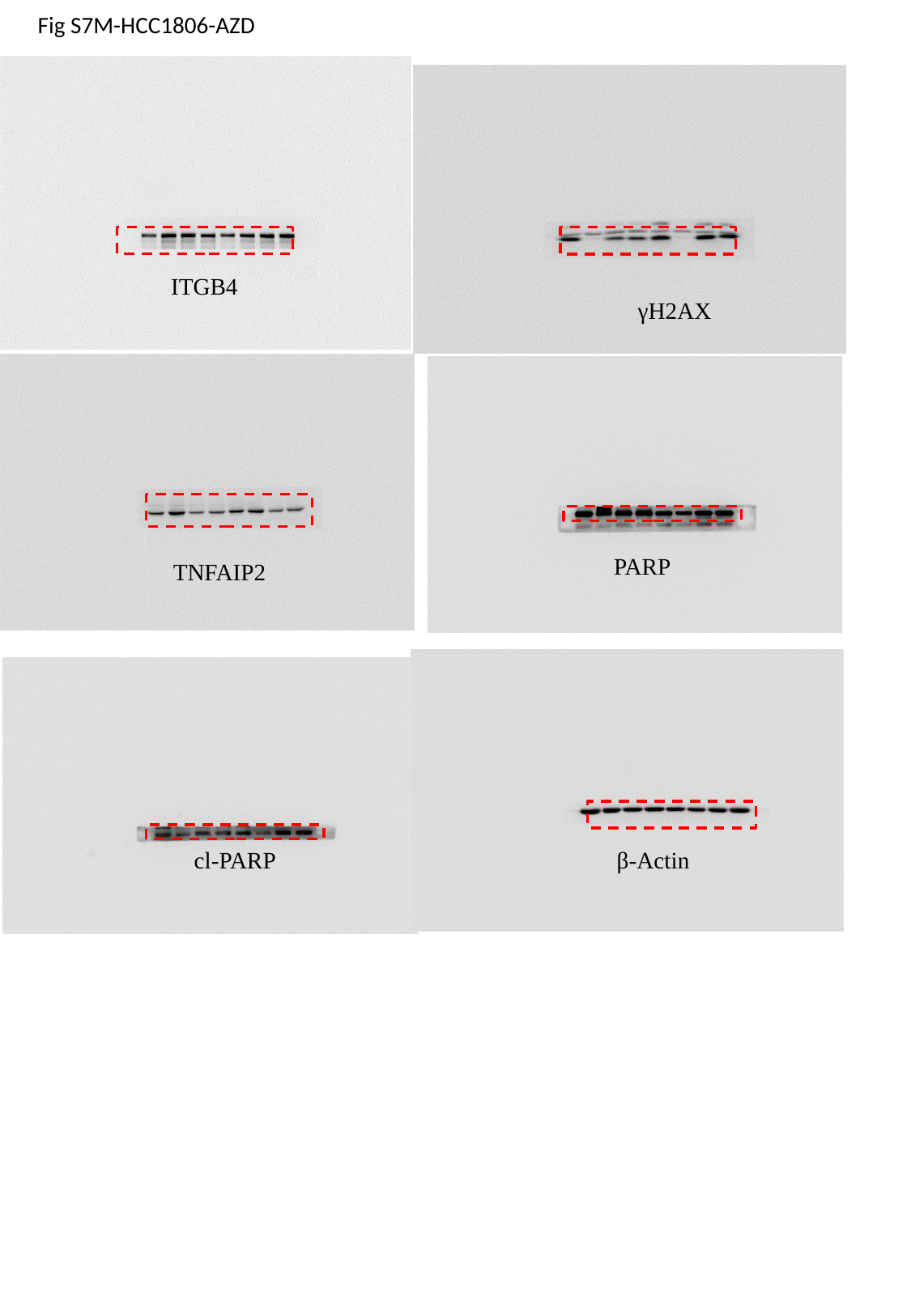

Fig S7M-HCC1806-AZD
ITGB4
γH2AX
PARP
TNFAIP2
cl-PARP
β-Actin

## Slide 5
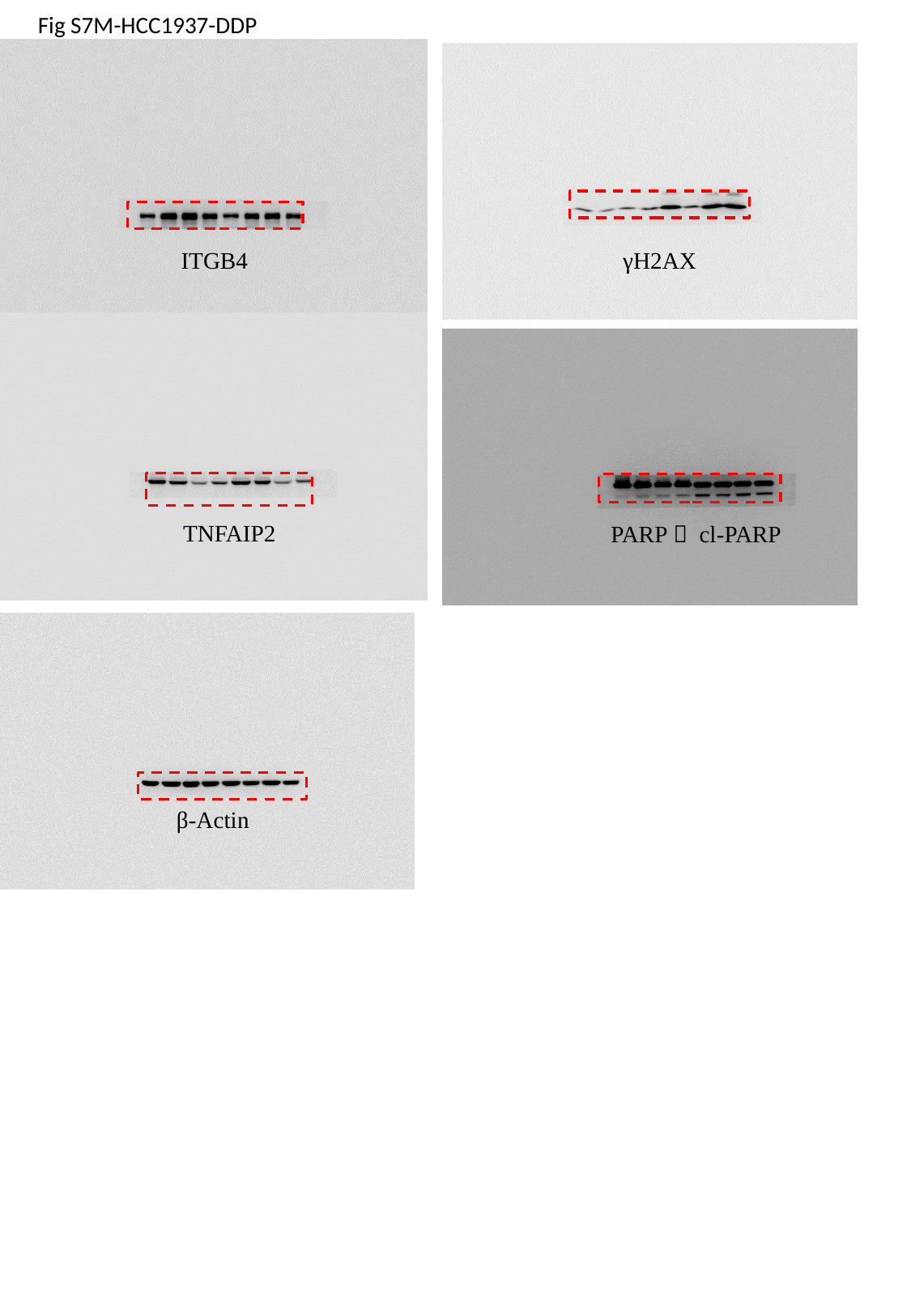

Fig S7M-HCC1937-DDP
γH2AX
ITGB4
TNFAIP2
PARP， cl-PARP
β-Actin

## Slide 6
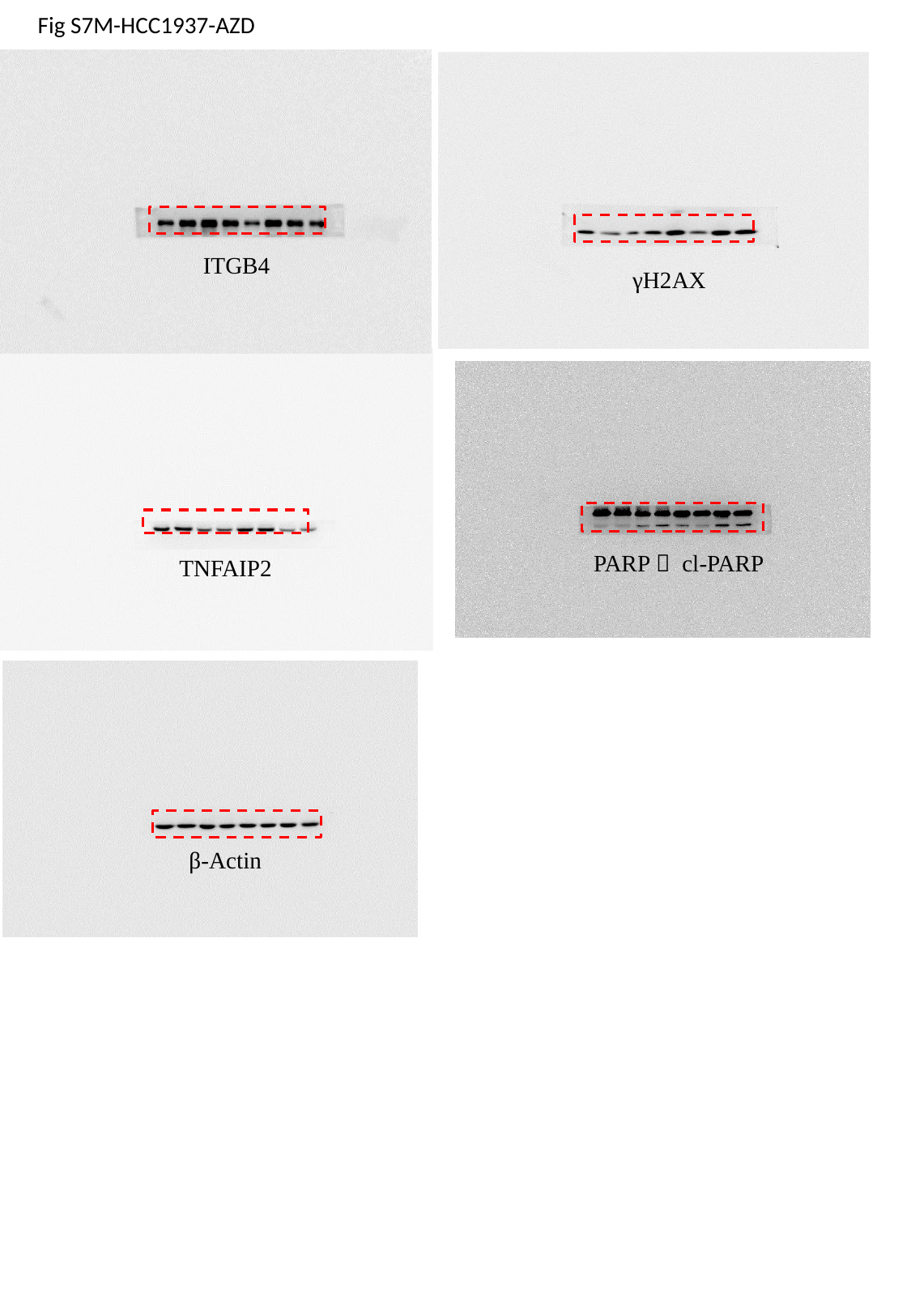

Fig S7M-HCC1937-AZD
ITGB4
γH2AX
PARP， cl-PARP
TNFAIP2
β-Actin

## Slide 7
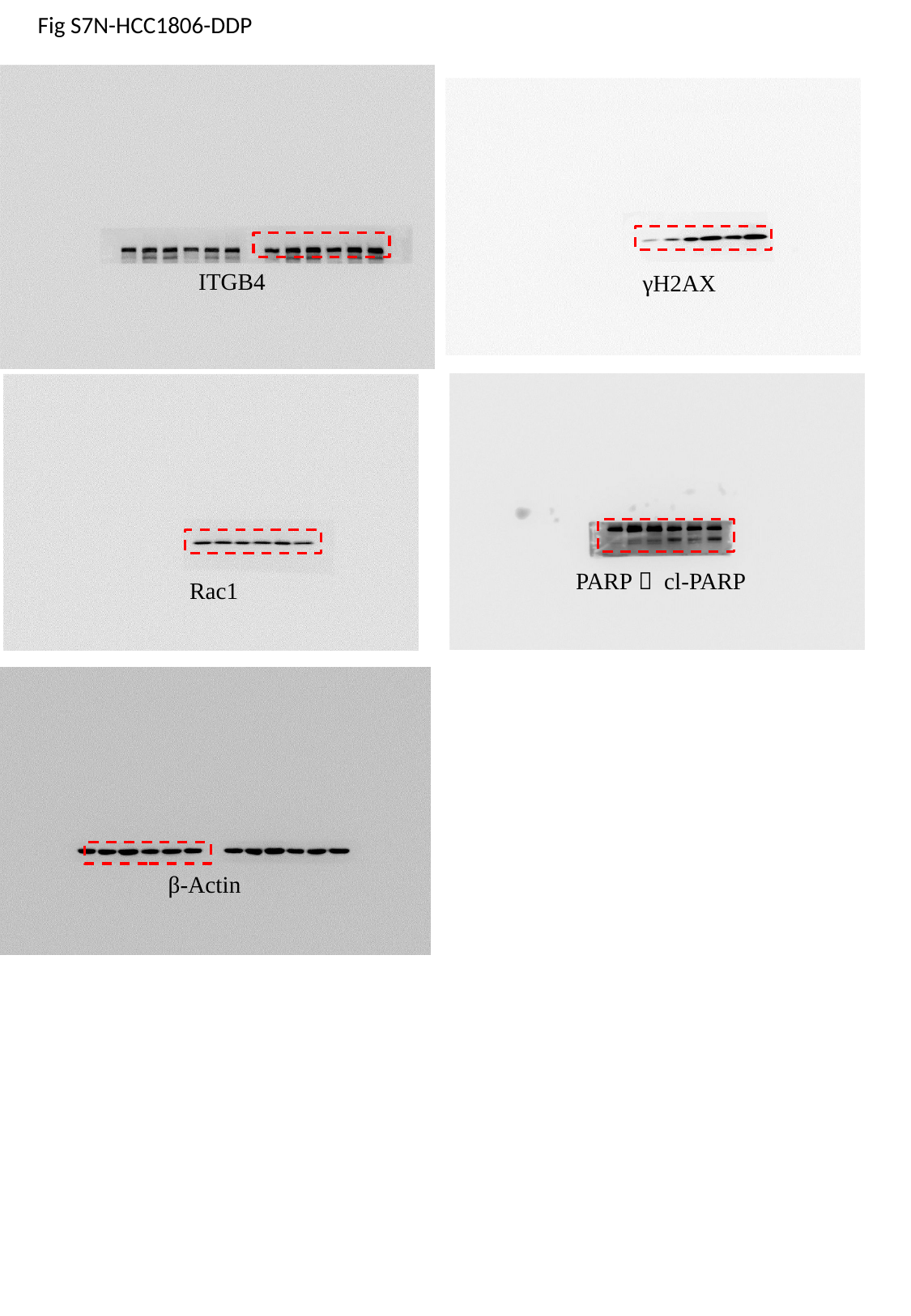

Fig S7N-HCC1806-DDP
ITGB4
γH2AX
PARP， cl-PARP
Rac1
β-Actin

## Slide 8
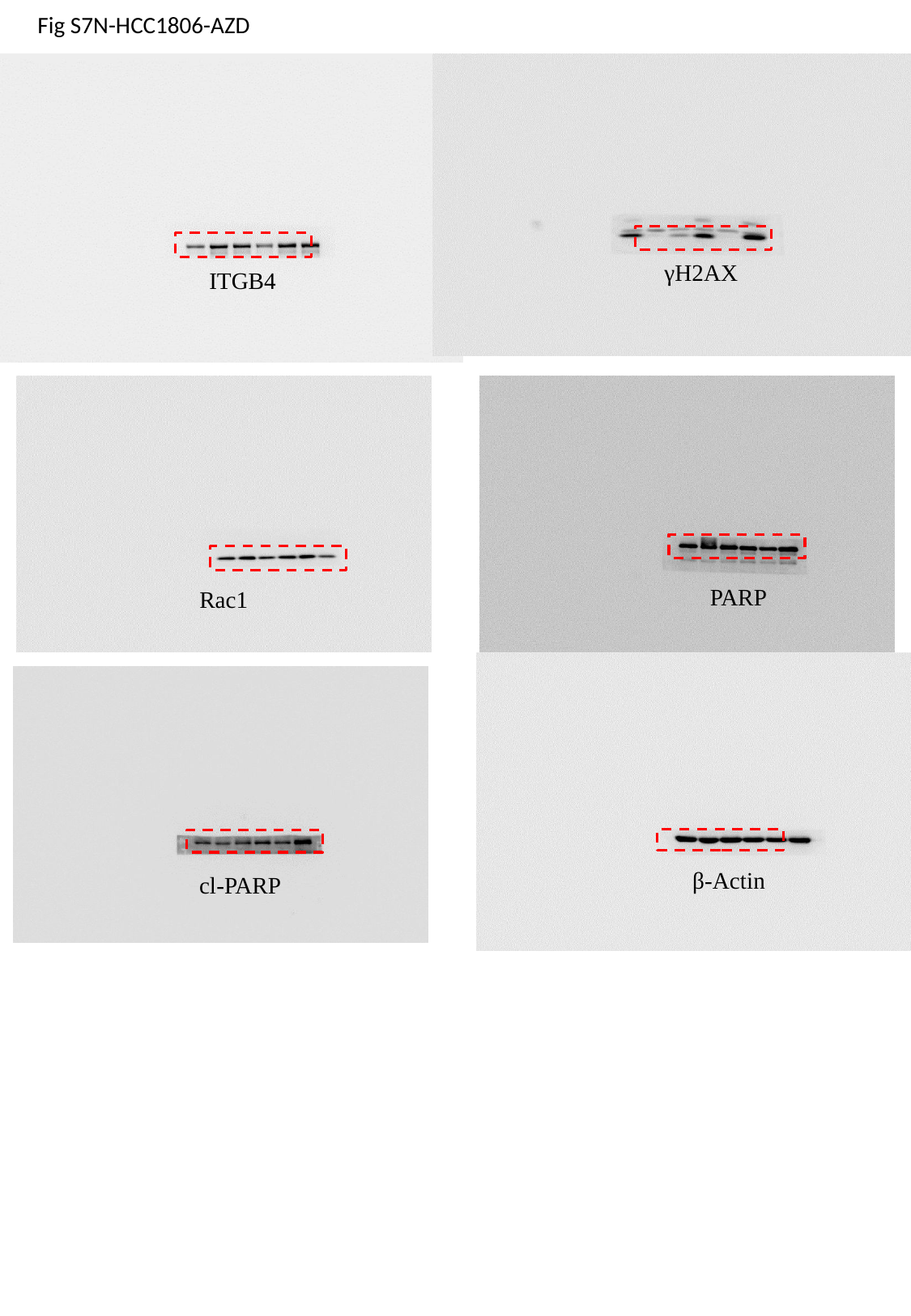

Fig S7N-HCC1806-AZD
γH2AX
ITGB4
PARP
Rac1
β-Actin
cl-PARP

## Slide 9
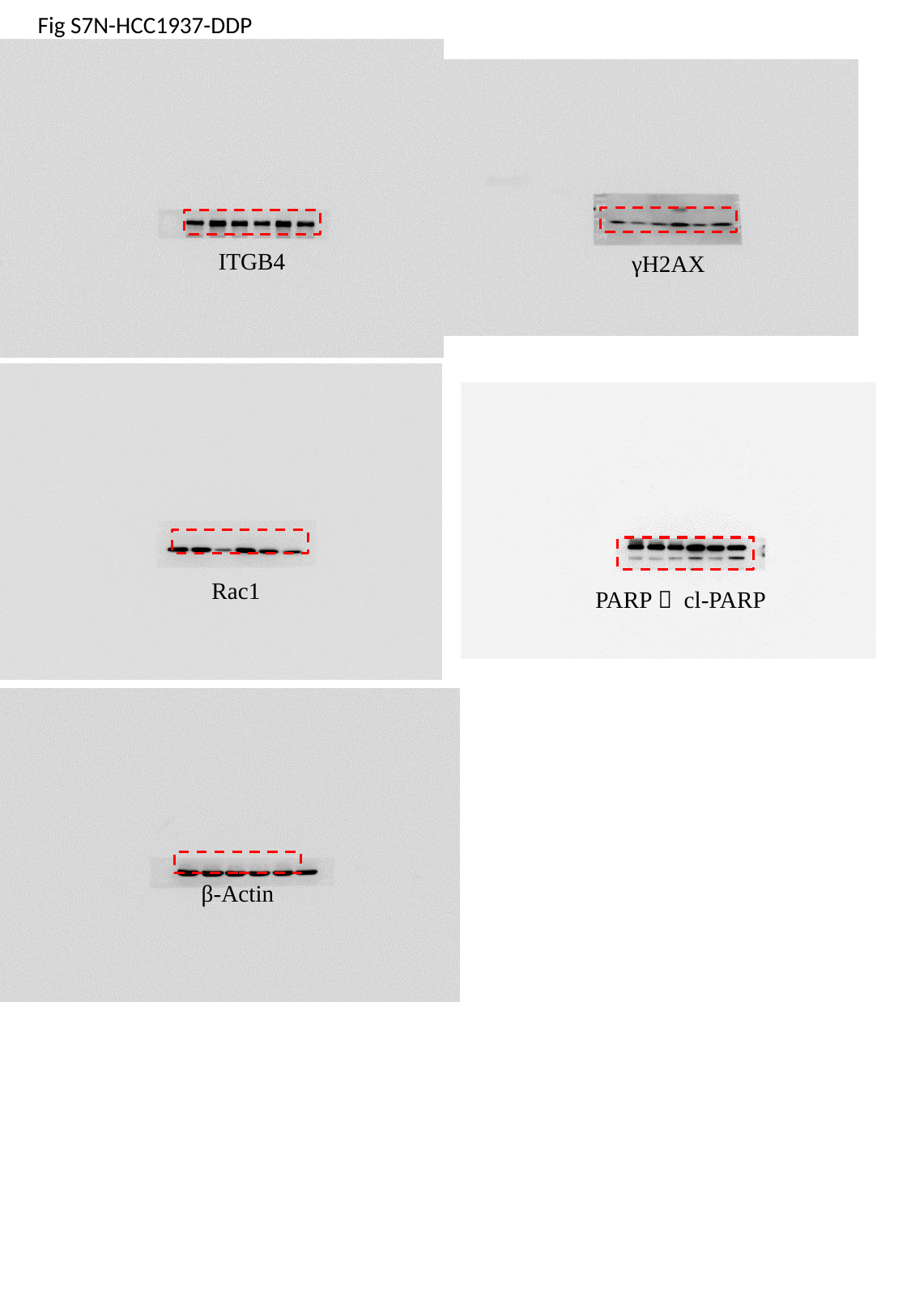

Fig S7N-HCC1937-DDP
ITGB4
γH2AX
Rac1
PARP， cl-PARP
β-Actin

## Slide 10
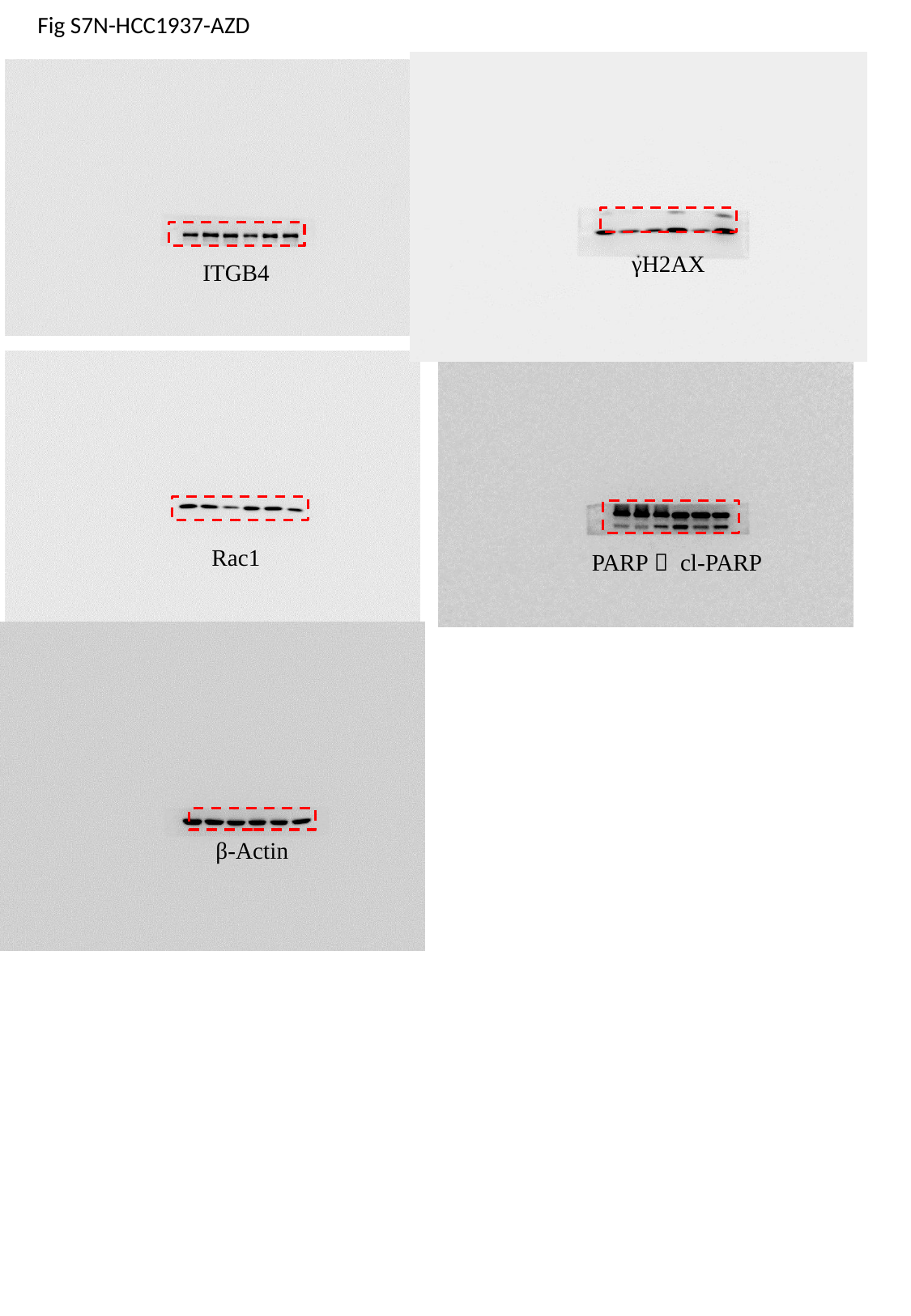

Fig S7N-HCC1937-AZD
γH2AX
ITGB4
Rac1
PARP， cl-PARP
β-Actin
